# The Oxime Ethers with Heterocyclic, Alicyclic and Aromatic Moiety as Potential Anti-Cancer Agents

**DOI:** 10.3390/molecules27041374

**Published:** 2022-02-17

**Authors:** Tomasz Kosmalski, Anna Hetmann, Renata Studzińska, Szymon Baumgart, Daria Kupczyk, Katarzyna Roszek

**Affiliations:** 1 Department of Organic Chemistry, Faculty of Pharmacy, Collegium Medicum in Bydgoszcz, Nicolaus Copernicus University in Toruń, Jurasza 2, 85-089 Bydgoszcz, Poland; sz.baumgart@cm.umk.pl; 2 Department of Biochemistry, Institute of Biology, Faculty of Biological and Veterinary Sciences, Nicolaus Copernicus University in Toruń, Lwowska 1, 87-100 Torun, Poland; ahetmann@umk.pl (A.H.); kroszek@umk.pl (K.R.); 3 Department of Medical Biology and Biochemistry, Faculty of Medicine, Collegium Medicum in Bydgoszcz, Nicolaus Copernicus University in Toruń, Karłowicza 24, 85-092 Bydgoszcz, Poland; dariak@cm.umk.pl

**Keywords:** benzofuran derivatives, thiophene derivatives, cytotoxicity, oxime ethers, human cancer cells, anti-cancer therapy

## Abstract

Chemotherapy is one of the most commonly used methods of cancer disease treatment. Due to the acquisition of drug resistance and the possibility of cancer recurrence, there is an urgent need to search for new molecules that would be more effective in destroying cancer cells. In this study, 1-(benzofuran-2-yl)ethan-1-one oxime and 26 oxime ethers containing heterocyclic, alicyclic or aromatic moiety were screened for their cytotoxicity against HeLa cancer cell line. The most promising derivatives with potential antitumor activity were 2-(cyclohexylideneaminoxy)acetic acid (**18**) and (*E*)-acetophenone *O*-2-morpholinoethyl oxime (**22**), which reduced the viability of HeLa cells below 20% of control at concentrations of 100–250 μg/mL. Some oxime ethers, namely thiazole and benzothiophene derivatives (**24**–**27**), also reduced HeLa cell viability at similar concentrations but with lower efficiency. Further cytotoxicity evaluation confirmed the specific toxicity of (*E*)-acetophenone *O*-2-morpholinoethyl oxime (**22**) against A-549, Caco-2, and HeLa cancer cells, with an EC50 around 7 μg/mL (30 μM). The most potent and specific compound was (*E*)-1-(benzothiophene-2-yl)ethanone *O*-4-methoxybenzyl oxime (**27**), which was selective for Caco-2 (with EC50 116 μg/mL) and HeLa (with EC50 28 μg/mL) cells. Considering the bioavailability parameters, the tested derivatives meet the criteria for good absorption and permeation. The presented results allow us to conclude that oxime ethers deserve more scientific attention and further research on their chemotherapeutic activity.

## 1. Introduction

The cancer diseases are a very serious health problem. Currently, about 18 million cases of various types of cancer are diagnosed each year. According to WHO predictions, this number is expected to reach 29.4 million in 2040 [1]. Chemotherapy is one of the most important methods of cancer treatment. Among the many compounds currently in use, new molecules are being developed that may be even more effective in destroying cancer cells. Success in treating cancer depends on the ability to detect it early, but also on the type of cancer. The problem in the treatment of neoplasms is the acquisition of drug resistance, and, unfortunately, the possibility of relapse of the neoplastic disease. The causes of cancer are difficult to estimate, but are certainly a very complex problem [2]. Therefore, there is a need to search for and investigate new structures that offer the potential to cure cancer.

The oxime ethers represent an interesting class of compounds with diverse biological activities. They exhibit antimicrobial [3], insecticidal [4], and anticonvulsant [5] properties and are often examined for their cytotoxic activity. Many articles related to the activities of oxime ethers concern their antitumor properties. Chakravati et al. reported the synthesis of thioarylnaphthylmethanone oxime ethers to treat breast cancer (MCF-7) cells [6]. The anti-tumor activities of the compounds against the cell lines tested were very high. Díaz et al. tested the cytotoxicity of oxime ethers of flavone and 6-hydroxy flavone oxime derivatives against breast (MDA-MB-231) and prostate (PC-3) adenocarcinomas as well as against human lung cells (A-549 and MRC-5 cell lines) [7]. Naringenin oxime derivative ethers showed very high antiproliferative activity in studies on promyelocytic leukemia (HL-60) and breast cancer cell lines (MCF-7, MDA-MB-231) [8]. Bis(4-hydroxy)benzophenone oxime ethers have been identified as novel estrogen receptor ligands [9]. These compounds also inhibit the growth of cancer cells through an estrogen receptor independent mechanism.

Recent years have indicated a growing research interest in benzofuran moiety, which significantly influences the biological activity of compounds. For example, benzofuran derivatives are being investigated for anticancer activity [10,11], as well as antibacterial, antiprotozoal and other activities [12,13]. Other heterocyclic moieties—thiazole and benzothiophene—may determine the biological activities of these compounds. Many of the thiazole derivatives show antitumor activity [14]. On the other hand, many interesting biological activities are exhibited by benzothiophene derivatives, including antitumor activity [15]. The benzofuran derivatives of the oxime ethers have also shown high antifungal activities against *Candida albicans* [16]. The thiophene derivative oxime ethers exhibit a number of biological activities, e.g., 2-benzoylthiophene derivatives show anti-aggregating activity against platelets [17] and antibacterial [18], anti-inflammatory, and anticonvulsant effects [19]. The derivatives *O*-uracil oxime of 2-acetylthiophene show antitumor effects [20], and in the case of many thiophene derivatives, it is also possible to present those structures that have shown antitumor activity [21]. Oxime ethers, for example, acetophenone derivatives, are also biologically active compounds and exhibit antidepressant activities [22].

Considering the above, oxime ethers constitute a potential class of compounds with possible and promising anti-cancer properties. Therefore, it seems justified to conduct research on the cytotoxic activity of various derivatives from this group of compounds.

In our previous studies, we obtained a series of new functionalized ethers of 2-acetylbenzofuran [23] and 2-acetylthiophene oxime [24]. The obtained products were tested microbiologically. In this report, we performed cytotoxicity studies of compounds with oxime ether moiety and benzofuran, benzothiophene, thiophene and thiazole substituents, as well as other cyclic substituents to assess their anti-proliferative potential. Thus, we have tested their influence on human dermal fibroblasts (HDF) used as healthy cell models and human cervical adenocarcinoma (HeLa) cells, lung epithelial carcinoma (A549) cells, and intestinal epithelial carcinoma (Caco-2) cell line representing different human cancer cells.

## 2. Results and Discussion

### 2.1. Chemistry

**Scheme 1:**
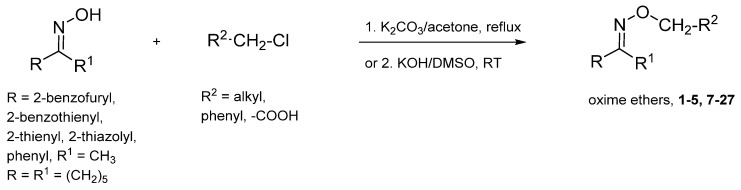
Synthesis of the tested compounds **1**–**5** and **7**–**27**.

Most of the presented compounds (**1**–**5**, **7**–**27**) were obtained by the reaction of oximes with the corresponding halides in the presence of K_2_CO_3_ or KOH base and were described in our earlier articles [23,24,25] (Scheme 1). The products were obtained with good yields and high purities. The following derivatives of thiophene (**13**–**15**), thiazole (**24**–**25**) and benzothiophene (**26**–**27**) are newly synthesized compounds. The compounds were obtained in the reaction of 2-acetylthiophene oxime (**13**–**15**), 2-acetylthiazole oxime (**24**–**25**) or 2-acetylbenzothiophene oxime (**26**–**27**) with corresponding chloroalkylamine in DMSO in KOH medium. The structures of all compounds together with their half-maximal effective concentrations (EC50) in HeLa cell culture are presented in Table 1.

### 2.2. Cytotoxicity Evaluation

Cytotoxic properties of all compounds were determined by 3-(4,5-dimethylthiazol-2-yl)-2,5-diphenyltetrazolium bromide (MTT) test reflecting cell viability. Cytotoxicity screening of all 27 compounds—2-acetylbenzofuran oxime (**6**) and its oxime ethers (**1**–**5**), as well as: 2-acetylthiophene (**7**–**17**), cyclohexanone (**18**–**20**), acetophenone (**21**–**23**), thiazole (**24**–**25**), benzothiazole (**26**–**27**) oxime ethers—was performed in vitro on human cervical adenocarcinoma (HeLa) cells as the representatives of cancerous cells.

**Figure 1:**
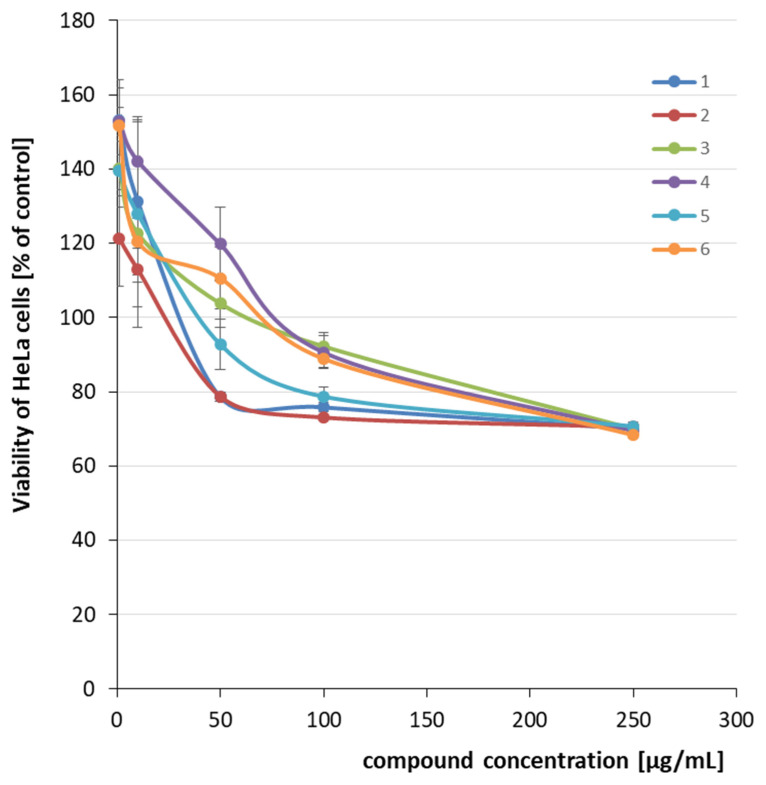
The dose-dependent curves for cytotoxicity screening of the benzofuran oxime ethers (**1**–**5**) and 2-acetylbenzofuran oxime (**6**) on HeLa cells. Each compound was added to growing cells at concentrations of 1, 10, 50, 100, and 250 µg/mL, and cell viability was assayed with the MTT test.

A majority of the derivatives are quite biocompatible in the HeLa cell culture, and in the concentration range of 1–50 μg/mL even increased the cell viability under study. This increase does not specifically mean that the number of cells increased; sometimes the stressor initiates higher metabolic activity (as we used the MTT assay reflecting activity of mitochondrial enzymes) that facilitates cell adaptation to stress conditions. All benzofuran derivatives (**1**–**6**) in the concentration range of 100–250 μg/mL reduced the survival of HeLa cells only to about 80–70% (Figure 1). The half-maximal effective concentrations (EC50) in HeLa cell culture were calculated and are summarized in Table 1.

**Figure 2:**
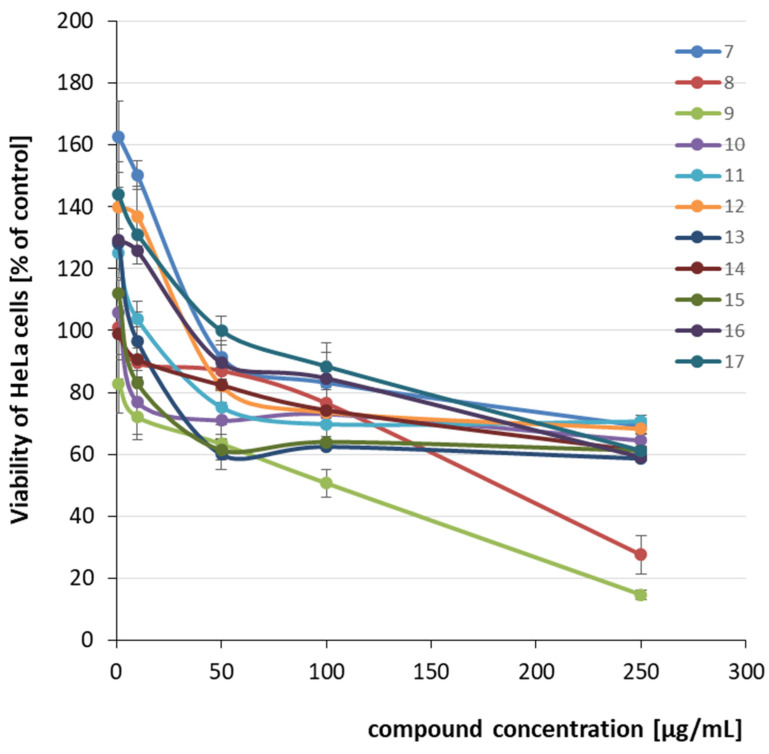
The dose-dependent curves for cytotoxicity screening of thiophene oxime ethers (**7**–**17**) on HeLa cells. Each compound was added to growing cells at concentrations of 1, 10, 50, 100, and 250 µg/mL, and cell viability was assayed with the MTT test.

Thiophene derivatives in the concentration range of 1–10 μg/mL were in general characterized by a lack of cytotoxicity against HeLa cells. On the other hand, compounds **7**, **12**, **16** and **17** in the concentration range of 1–10 μg/mL increased cell viability, and in the concentration range of 50–250 μg/mL slightly reduced the cell viability to about 70–60% (Figure 2). The most promising compounds in this group were **8** and **9** with an EC50 value of approximately 100 μg/mL (Table 1).

**Figure 3:**
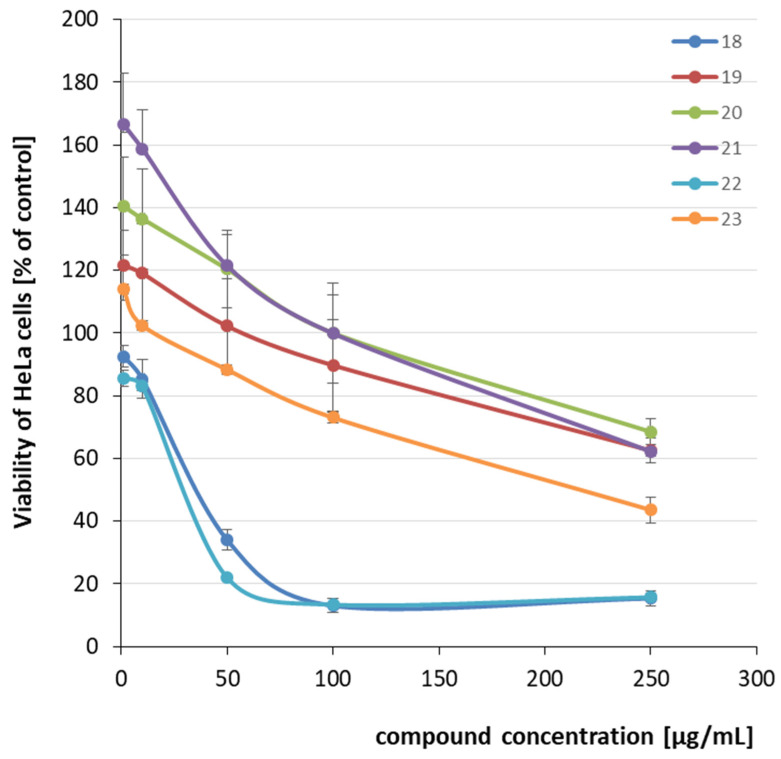
The dose-dependent curves for cytotoxicity of the cyclohexanone and acetophenone oxime ethers (**18**–**23**) on HeLa cells. Each compound was added to the growing cells at concentrations of 1, 10, 50, 100, and 250 µg/mL, and cell viability was assayed with the MTT test.

The results of studies using cyclohexanone and acetophenone derivatives on HeLa cell culture indicated no cytotoxicity in the concentrations of 1 and 10 μg/mL. The most promising derivatives were **18** and **22**, as they reduced the viability of HeLa cells to below 20% at a concentration range of 50–250 μg/mL, and EC50 was achieved after 24 h culture at the concentrations of 32.26 and 23.15 μg/mL for (**18**) and (**22**), respectively (Figure 3, Table 1).

The last group of oxime ethers were the thiazole and benzothiophene derivatives (*E*)-1-(thiazol-2-yl)ethanone *O*-4-methoxybenzyl oxime (**24**) and (*E*)-1-(thiazol-2-yl)ethanone *O*-4-bromobenzyl oxime (**25**), and the benzothiophene derivatives (*E*)-1-(benzothiophene-2-yl)ethanone *O*-2,4-dichlorobenzyl oxime (**26**) and (*E*)-1-(benzothiophene-2-yl)ethanone *O*-4-methoxybenzyl oxime (**27**).

**Figure 4:**
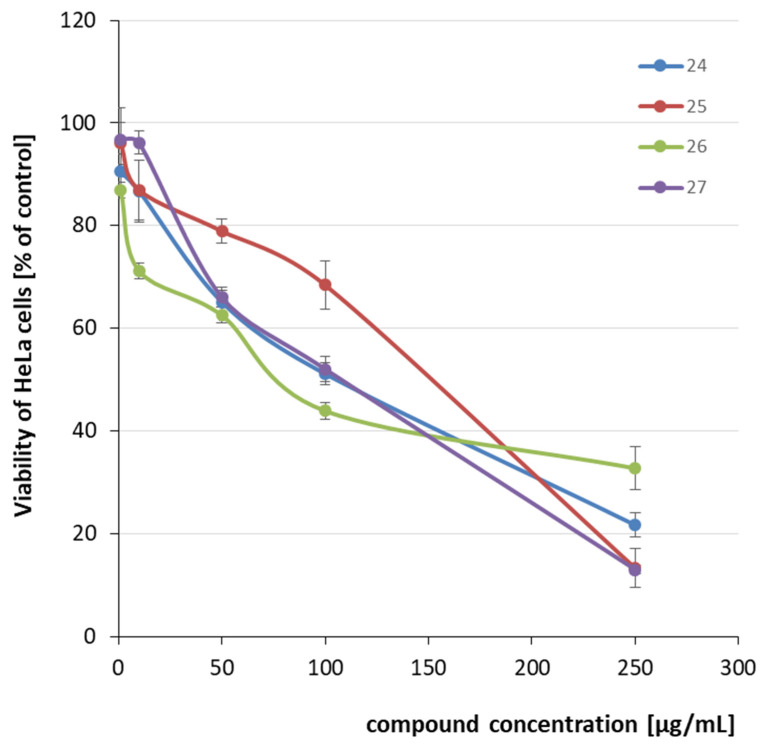
The dose-dependent curves for cytotoxicity of the thiazole and benzothiophene oxime ethers (**24**–**27**) on HeLa cells. Each compound was added to the growing cells at concentrations of 1, 10, 50, 100, and 250 µg/mL, and cell viability was assayed with the MTT test.

Based on the obtained outcomes, we can conclude that all derivatives from this group have cytotoxic activity against HeLa cells only at the highest concentration of 250 μg/mL, but reduce their viability even to below 20% (Figure 4, Table 1).

**Figure 5:**
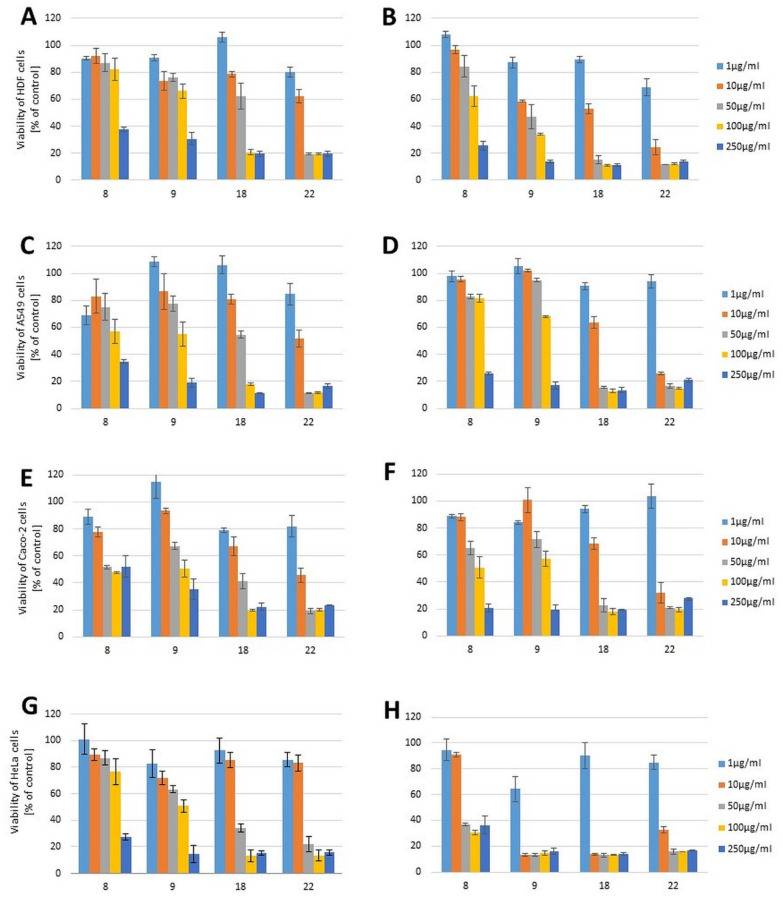
The cytotoxicity of selected oxime ether derivatives (**8**, **9**, **18**, and **22**) towards: (**A**)—HDF after 24 h, (**B**)—HDF after 72 h, (**C**)—A549 after 24 h, (**D**)—A549 after 72 h, (**E**)—Caco-2 cells after 24 h, (**F**)—Caco-2 cells after 72 h, (**G**)—HeLa cells after 24 h, and (**H**)—HeLa cells after 72 h in culture.

**Figure 6:**
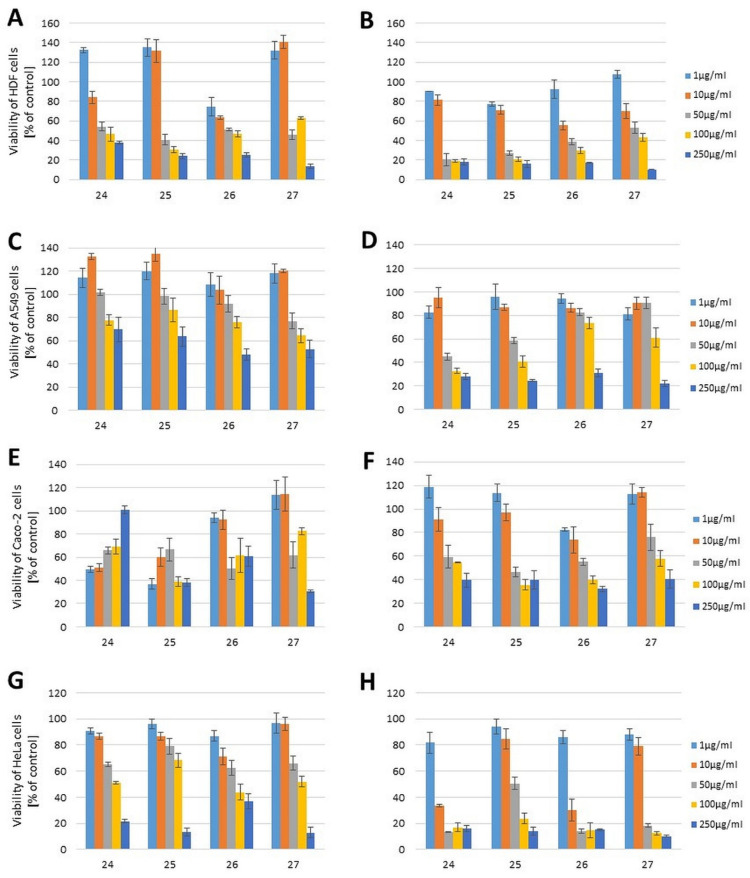
The cytotoxicity of the oxime ethers **24**–**27** towards: (**A**)—HDF after 24 h, (**B**)—HDF after 72 h, (**C**)—A549 after 24 h, (**D**)—A549 after 72 h, (**E**)—Caco-2 cells after 24 h, (**F**)—Caco-2 cells after 72 h, (**G**)—HeLa cells after 24 h and (**H**)—HeLa cells after 72 h in culture.

The screening results indicate that some of the compounds have the potential to act as anti-cancer agents and deserve more detailed assessment. Therefore, compounds showing the highest cytotoxicity (**8**, **9**, **18**, **22**, **24**–**27**) were further tested on normal human dermal fibroblasts (HDF), A549, Caco-2, and HeLa cells to compare their activity after 24 h and 72 h culture (Figure 5 and Figure 6).

The most potent derivatives were compounds **18** and **22**, as they reduced the viability of HDF, A549, Caco-2, and HeLa cells to approximately 20% at a concentration of 100 μg/mL for **18** and 50 μg/mL for **22** after 24 h exposure (Figure 5). Unfortunately, there was no clear specificity towards cancer cell lines, mainly for compound **18**, as its EC50 value after 72 h culture was similar for all types of cells, excluding HeLa, and calculated as 90–100 μM. The low sensitivity of cancer cells is their specific and undesired feature, one of the hallmarks of cancer, or a result of the presence of highly resistant cancer stem cells [26,27]. A small difference in EC50 for compound **22** observed between normal and cancer cells allowed us to assume that this type of modification could be promising for the design of new anti-cancer agents, summarized in Table 2.

Compounds **24**–**27** showed time- and dose-dependent cytotoxicity in relation to the tested cell lines. As predicted, their influence on normal and cancer cells was comparable (Figure 6). On the other hand, compound **27** exhibited a specific cytotoxic activity against Caco-2 and HeLa cells, while exhibiting relatively low toxicity against HDF cells. A decrease in HDF viability by 50% after 72 h exposure required a 0.45 mM concentration of this agent, whereas concentrations of 0.37 mM and 0.09 mM were sufficient against Caco-2 cells and HeLa cells, respectively (Table 2).

**Figure 7:**
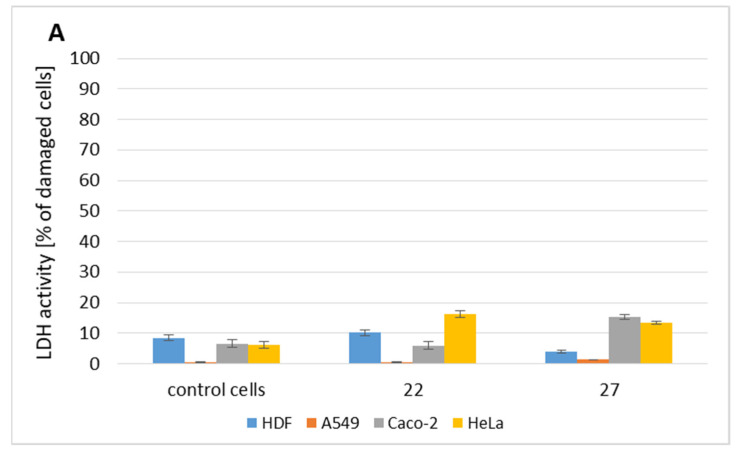

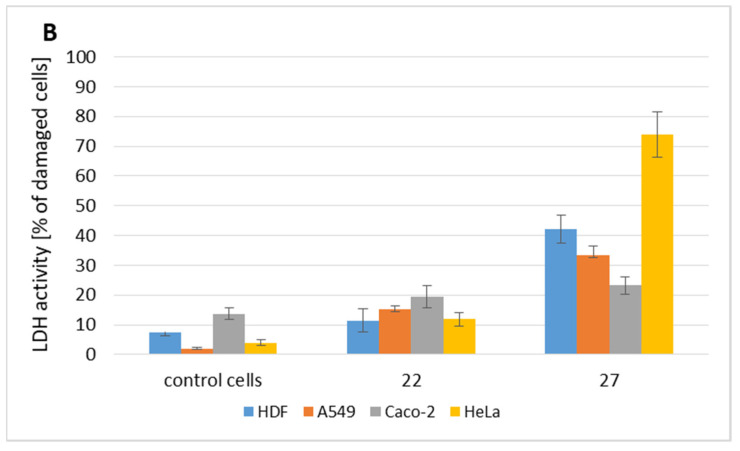
Comparison of LDH activity (relative to positive control where appropriate cell lysates were assumed as 100% damaged cells) in cell media after 24 h (**A**) and 72 h (**B**) of exposure to concentrations equal to EC50 value of selected compounds. The cells not treated with tested compounds were treated as negative controls.

Summing up, only a few of the 27 tested derivatives could be promising as potential anti-cancer agents. Two of them—compound **22** and **27**—would be a good starting point for future research. Therefore, we sought to compare their ability to induce necrotic cell death. Since necrotic cell death relies on the disruption of cell membrane, it results in the extracellular release of cytoplasmic enzymes such as lactate dehydrogenase (LDH). Thus, we compared LDH activity in the extracellular environment (culture media). The results are presented in Figure 7.

Surprisingly, compound **22**, although toxic to cells, induced only a slight increase in LDH activity outside the cells, not exceeding 20% that of positive control. It can be assumed that its mode of action is underpinned by a decrease in proliferative rate and/or apoptotic cell death. Conversely, compound **27** induced extreme membrane damage and release of LDH, primarily in HeLa cells but not in Caco-2 cells, which also suffer from this compound’s toxicity. The higher LDH activity after 72 h of cell exposure to toxic compounds confirmed that initially the cells decreased their proliferation and subsequently necrosis occurred. It can be concluded that antitumor activity of various tested compounds depends on their chemistry, concentration and, on the other hand, also on the specific features of the cells tested.

### 2.3. Bioavailability of Tested Compounds

Lipinski’s rule and Veber’s rule are the main principles considered in medicinal chemistry when evaluating the bioavailability of drugs after oral administration. According to Lipinski’s rule, a compound is characterized by good absorption and permeation after oral administration when it does not exceed more than one of the following descriptors: molecular mass ≤ 500 Da, log *p* ≤ 5, number of hydrogen donors (nOHNH) ≤ 5 and number of hydrogen acceptors ≤ 10 [28]. On the other hand, by Veber’s rule, a compound is characterized by good bioavailability after *per os* administration when the topological polar surface of the molecule ≤ 140 Å^2^ and the number of rotatable bonds (Nrotb) < 10 [29]. The above parameters were calculated for compounds **1**–**27** using Molinspiration software [30], and the results are presented in Table 3.

Analysis of the calculated parameters showed that all the tested ethers satisfy Veber’s rule, while the rule of five without any violations was satisfied by compounds **4**–**25**. For the ethers **1**–**3** and **26**–**27,** there was one violation of the rule of five (log *p* > 5).

The topological polar surface area (TPSA) of a molecule is an important descriptor considered during drug design, due to the fact that it allows predicting the permeation of a compound across biological membranes, including but not limited to penetration of the blood–brain barrier [31]. Molecules having topological polar surface area values of less than 60–70 Å^2^ show a high probability of penetrating the blood–brain barrier [32]. Among the studied ethers, derivatives **4** and **10** had TPSA >60–70 Å^2^, while the remaining compounds did not exceed 70 Å^2^, which suggests that they would be characterized by high penetration into the central nervous system. Considering the bioavailability parameters, the tested derivatives met the criteria for good absorption and permeation.

Regarding their potential anti-cancer activity, the selected oxime ether derivatives can be described as promising therapeutic compounds for cancer disease. The presented outcomes created a basis for further research on the oxime ethers with cytotoxic activity. We hope that it will pave the way for further structural modifications of these compounds by addition of different substituents in order to increase their activity, and to evaluate their modes of action in detail.

## 3. Materials and Methods

### 3.1. General Information

^1^H- and ^13^C-NMR spectra were recorded on the Bruker Avance 400 and 700 apparatus (TMS as an internal standard). High-resolution mass spectrometry (HRMS) measurements were made using a Synapt G2 Si mass spectrometer (Waters) equipped with an ESI source and a quadrupole time-of-flight mass analyzer. In order to achieve the highest accuracy of mass measurement, data were collected in center of gravity mode and the mass was corrected during acquisition using enkephalin leucine solution as external reference (Lock-SprayTM), which generated reference ion at *m*/*z* 556.2771 Da ([M + H]^+^) in positive ESI mode. The measurement results were processed with MassLynx 4.1 software (Waters).

### 3.2. Reagents and Solvents

*Solvents:* chloroform, dimethylsulfoxide, ethyl acetate, ethyl alcohol, hexane, triethylamine—POCh, Poland (Avantor Performance Materials Poland S.A., Gliwice Poland).

*Reagents for synthesis:* 2-acetylhiophene, 2,4-dichlorobenzyl chloride, 4-methoxybenzyl chloride, 2-chloroethylamine hydrochloride 99%, 3-chloropropylamine hydrochloride 98%, 2-chloro-*N,N*-diethylethylamine hydrochloride 99%, Sigma Aldrich Poznań Poland. 2-Acetylbenzothiophene 98% and 2-acetylthiazole 98% AmBeed (Chemat, Poland).

*Auxiliary reagents:* hydroxylamine hydrochloride, magnesium sulfate, potassium iodide, potassium hydroxide—POCh Poland (Avantor Performance Materials Poland S.A., Gliwice Poland).

*TLC:* 5 cm × 10 cm TLC plates coated with silica gel with F-254 (Merck, Darmstadt Germany).

### 3.3. Synthesis of Compounds **13**–**15**, **24**–**25** and **26**–**27**—General Procedure 

In a flask (25 mL) equipped with a plug, to a solution of 2-acetylthiophene oxime (5.0 mmol) for the synthesis oxime ethers **13**–**15**, (2-acetylthiazole oxime to the synthesis **24**–**25**, 2-acetylbenzothiophene oxime to the synthesis **26**–**27**) and DMSO (6 mL), an appropriate chloride (5.0 mmol), KI (0.10 g), and strongly pulverized KOH (20 mmol) were added [25]. The mixture was intensively stirred for 1 h at room temperature (monitored by TLC). Water (30 mL) and chloroform (30 mL) were added. The water phase was extracted with chloroform (30 mL). The combined organic layers were washed with water (4 × 25 mL) and dried (MgSO_4_). Evaporation yielded the oxime ethers.

For the preparation oxime ethers **24**–**25** and **26**–**27** the same procedure was used, with the exception of the amount of KOH—10 mmol. 

NMR and MS spectra of the tested compounds are available in Supplementary Materials. (*E*)-1-(thiophen-2-yl)ethanone *O*-2-aminoethyl oxime (**13**)—Yield: 71%, pale yellow oil. ^1^H-NMR (500 MHz, CDCl_3_), δ ppm, J Hz): 7.27 (d, 5.0, 1H, CH), 7.22 (dd, 1.0 3.5, 1H, CH), 7.02 (dd, 1.0 5.0, 1H, CH), 4.20 (t, 5.0, 2H, CH_2_), 3.02 (t, 5.0, 2H, CH_2_), 2.27 (s, 3H, CH_3_), 1.6–1.8 (s, 2H, NH_2_). ^13^C-NMR (125 MHz, CDCl_3_, δ ppm): 150.83 (C_C=N_), 140.41 (C), 126.97 (CH), 126.77 (CH), 126.14 (CH), 76.63 (OCH_2_), 41.69 (NCH_2_), 12.92 (CH_3_). HR-MS *m*/*z* 185.0752 [M + H]^+^ (calcd for C_8_H_13_N_2_OS: 185.0743). R_f_ (silica gel, AcOEt:hexane:triethylamine 1:1:0.02): 0.05.

(*E*)-1-(thiophen-2-yl)ethanone *O*-2-(diethylamino)ethyl oxime (**14**)—Yield: 78%, orange oil. ^1^H-NMR (400 MHz, CDCl_3_), δ ppm, J Hz): 7.26 (dd, 1.2 4.0, 1H, CH), 7.21 (dd, 1.2 4.0, 1H, CH), 7.02 (dd, 1.2 4.0, 1H, CH), 4.27 (t, 2H, OCH_2_), 2.85 (t, 2H, NCH_2_), 2.65 (q, 4H, 2 × CH_2_), 2.26 (s, 3H, CH_3_), 1.08 (t, 7.2, 6H, 2 × CH_3_). ^13^C-NMR (100 MHz, CDCl_3_, δ ppm): 150.41 (C_C=N_), 140.62 (C), 126.89 (CH), 125.62 (CH), 125,91 (CH), 72.70 (OCH_2_), 51.31 (CH_2_), 47.86 (NCH_2_, 2 × CH_2_), 12.98 (CH_3_), 11.87 (2 × CH_3_). HR-MS *m*/*z* 241.1373 [M + H]^+^ (calcd for C_12_H_21_N_2_OS: 241.1369). R_f_ (silica gel, AcOEt:hexane:triethylamine 1:1:0.02): 0.2.

(*E*)-1-(thiophen-2-yl)ethanone *O*-3-aminopropyl oxime (**15**)—Yield: 76%, pale yellow oil. ^1^H-NMR (500 MHz, CDCl_3_), δ ppm, J Hz): 7.27 (d, 5.0 1H, CH), 7.22 (d, 3.5, 1H, CH), 7.02 (dd, 1.0 5.0, 1H, CH), 4.26 (t, 6.0 Hz, 2H, CH_2_), 2.87 (dd, 6.5 7.0, 2H, CH_2_), 2.26 (s, 3H, CH_3_), 1.90–2.10 (s, 2H, NH_2_), 1.90 (m, 2H, CH_2_). ^13^C-NMR (125 MHz, CDCl_3_, δ ppm): 150.33 (C_C=N_), 140.63 (C), 126.89 (CH), 126.59 (CH), 125.90 (CH), 72.03 (OCH_2_), 39.20 (NCH_2_), 33.15, 12.85 (CH_3_). HR-MS *m*/*z* 199.0909 [M + H]^+^ (calcd for C_9_H_15_N_2_OS: 199.0900). R_f_ (silica gel, AcOEt:hexane:triethylamine 1:1:0.02): 0.05.

(*E*)-1-(thiazol-2-yl)ethanone *O*-4-methoxybenzyl oxime (**24**)—Yield: 84%, yellow oil. ^1^H NMR: (500 MHz, CDCl_3_): *δ* (ppm) 7.84 (d, 3.0, 1H, CH), 7.38 (d, 8.5, 2H, 2 × CH), 7.30 (d, 3.0, 1H, CH), 6.93 (d, 8.5, 2H, 2 × CH), 5,21 (s, 2H, OCH_2_), 3.83 (s, 3H, OCH_3_), 2.41 (s, 3H, CH_3_). ^13^C NMR: (125 MHz, CDCl_3_): *δ* (ppm) 165.44 (C=N), 159.58 (C), 151.88 (C), 142.79 (CH), 130.21 (2 × CH), 129.35 (C), 120.07 (CH), 113.84 (2 × CH), 76.79 (CH_2_), 55.28 (OCH_3_), 12.14 (CH_3_). HR-MS *m*/*z* 263.0848 [M + H]^+^ (calcd for C_13_H_15_N_2_O_2_S: 263.0849). R_f_ (silica gel, AcOEt:hexane 1:2): 0.85.

(*E*)-1-(thiazol-2-yl)ethanone *O*-4-bromobenzyl oxime (**25**)—Yield: 69%, white solid, mp. 67–70 °C (ethanol). ^1^H NMR: (500 MHz, CDCl_3_): *δ* (ppm) 7.85 (d, 3.5, 1H, CH), 7.52 (d, 8.5, 2H, 2 × CH), 7.29–7.32 (m, 3H, 3×CH), 5.22 (s, 2H, OCH_2_), 2.41 (s, 3H, CH_3_). ^13^C NMR: (125 MHz, CDCl_3_): *δ* (ppm) 165.05 (C=N), 152.39 (C), 142.87 (CH), 136.36 (C), 131.59 (2 × CH), 130.03 (2 × CH), 122.10 (C), 120.24 (CH), 76.12 (CH_2_), 12.18 (CH_3_). HR-MS *m*/*z* 310.9856 [M + H]^+^ (calcd for C_12_H_12_N_2_OS^79^Br: 310.9848). R_f_ (silica gel, AcOEt:hexane 1:1): 0.85.

(*E*)-1-(benzothiophene-2-yl)ethanone *O*-2,4-dichlorobenzyl oxime (**26**)—Yield: 51%, pale yellow solid, mp. 113–117 °C (ethanol). ^1^H-NMR: (500 MHz, CDCl_3_): δ (ppm) 7.79 (dd, 2.0 6.5 1H, CH), 7.77 (dd, 2.0 6.5 1H, CH), 7.48 (s, 1H, CH), 7.44 (dd, 2.0 8.0, 1H, CH), 7.35 (ddd, 2.0 5.5 6.0, 2H, 2 × CH), 7.29 (dd, 2.0 8.0, 1H, CH), 5.34 (s, 2H, OCH_2_), 2.38 (s, 3H, CH_3_). ^13^C NMR: (125 MHz, CDCl_3_): *δ* (ppm) 151.90 (C=N), 140.39 (C), 140.06 (C), 139.33 (C), 134.18 (CH), 134.11 (C), 130.82 (CH), 129.25 (CH), 127.05 (CH), 125.56 (CH), 124.39 (C), 123.92 (CH), 123.52 (CH), 122.26 (CH), 72.96 (CH_2_), 12.65 (CH_3_). HR-MS *m*/*z* 350.0172 [M + H]^+^ (calcd for C_17_H_14_NOSCl_2_: 350.0168). R_f_ (silica gel, AcOEt:hexane 1:2): 0.8.

(*E*)-1-(benzothiophene-2-yl)ethanone *O*-4-methoxybenzyl oxime (**27**)—Yield: 51%, white solid, mp. 132–134 °C (ethanol). ^1^H NMR: (500 MHz, CDCl_3_): *δ* (ppm) 7.79 (dd, 2.5 6.0, 1H, CH), 7.74 (dd, 2.5 6.0, 1H, CH), 7.44 (s, 1H, CH), 7.41 (d, 8.5, 2H, 2 × CH), 7.35 (dd, 2.0 5.5, 2H, 2 × CH), 6.94 (d, 8.5, 2H, 2 × CH), 5.20 (s, 2H, OCH_2_)_,_ 3.84 (s, 3H, OCH_3_), 2.32 (s, 3H, CH_3_). ^13^C NMR: (125 MHz, CDCl_3_): *δ* (ppm) 159.41 (C=N), 150.99 (C), 140.97 (C), 140.01 (C), 139.44 (C), 130.27 (2 × CH), 129.66 (C), 125.38 (CH), 124.32 (CH), 123.83 (CH), 123.06 (CH), 122.23 (CH), 113.80 (2 × CH), 76.40 (CH_2_), 55.28 (OCH_3_), 12.61 (CH_3_). HR-MS *m*/*z* 312.1054 [M + H]^+^ (calcd for C_18_H_18_NO_2_S: 312.1053). R_f_ (silica gel, AcOEt:hexane 1:2): 0.7.

### 3.4. Cytotoxicity Evaluation of Tested Compounds

2-Acetylbenzofuran, 2-acetylbenzothiophene, 2-acetylthiophene, 2-acetylthiazole, cyclohexanone and acetophenone derivatives were used for in vitro cytotoxicity assays with cervical adenocarcinoma (HeLa), human dermal fibroblasts (HDF), lung epithelial carcinoma (A549), and intestinal epithelial carcinoma (Caco-2) cell lines. A549, Caco-2, and HeLa cells were obtained from Sigma-Aldrich (Germany), and HDF from Biokom (Poland). All cell lines were cultured according to the manufacturer’s protocol under sterile conditions in the presence of 4.9% CO_2_ at 37 °C. A549 cells were grown in F-12 medium containing 10% fetal bovine serum (FBS) and 1% penicillin/streptomycin. HDF cells were grown in DMEM (low glucose, with 2 mM glutamine) medium containing 10% fetal bovine serum (FBS) and 1% penicillin/streptomycin. Caco-2 cells were grown in EMEM medium containing 10% fetal bovine serum (FBS), 1% non-essential amino acids (NEAA) and 1% penicillin/streptomycin. HeLa cells were grown in DMEM (low glucose, with 2 mM glutamine) medium containing 10% fetal bovine serum (FBS) and 1% gentamycin. After thawing, cells were cultured until they reached sub-confluent state. The cells were then detached using 0.25% trypsin solution and seeded into 24-well or 96-well plates (3 × 10^3^ cells per well) for further experiments. After 24 h growth, the tested compounds were added to the cell cultures in concentrations of 1, 10, 50, 100 and 250 μg mL^−1^, respectively, and incubated for the next 24 h or 72 h. The MTT test, based on the ability to reduce 3-(4,5-dimethylthiazol 2-yl)-2,5-diphenyltetrazolium bromide (MTT) by mitochondrial dehydrogenases, was performed in triplicate to assess cell metabolic activity and viability. The plates were then read spectrophotometrically at a wavelength of 570 nm. The dose-dependence curves were charted directly on x-/y-axes, with the drug dosages on the *x*-axis and the cell viability (percentage of viable cells) on the *y*-axis.

Additionally, the lactate dehydrogenase (LDH) activity test reflecting cell-membrane damage was performed for cells exposed to selected tested compounds. LDH activity was determined in the culture medium by measuring the decrease in NADH (nicotinamide adenine dinucleotide, reduced disodium salt). The decrease in the amount of NADH was directly correlated to the increase in the number of damaged cells. To measure LDH activity, the culture medium was collected from the wells with cells exposed to concentrations equal to the EC50 values of tested compounds after short- (24 h), and long-time (72 h) exposure. The LDH activity test was performed as follows: 25 μL of NADH (2.5 mg/mL) and 25 μL of sodium pyruvate (2.5 mg/mL) were added to 150 μL of the collected culture medium. Absorbance at 340 nm was spectrophotometrically measured. The number of damaged cells was compared to the untreated control sample, also considering the positive control treated with 1% Triton X-100 as 100% damaged cells.

## 4. Conclusions

In conclusion, 1-(benzofuran-2-yl)ethan-1-one oxime and 26 oxime ethers containing benzofuran, benzothiophene, thiophene, thiazole, cyclohexyl or phenyl moiety were screened for cytotoxicity against the human cervical adenocarcinoma (HeLa cell line) representative cancer cell model. The most promising derivatives were 2-(cyclohexylideneaminoxy)acetic acid (**18**) and (*E*)-acetophenone *O*-2-morpholinoethyl oxime (**22**), which reduced the viability of HeLa cells to below 20% of control at concentrations over 100 μg/mL. However, in further evaluations of cytotoxic influence, compound (**18**) showed higher toxicity against normal cells than cancerous cells, a common feature of many molecules that results from the lower sensitivity of cancer cells. We confirmed the specific toxicity of acetophenone derivative (**22**) against the cancer cell lines (EC50 ≈ 30 µM for Caco-2, HeLa, and A549 cells after 72 h treatment). From another group of oxime ether derivatives, compound **27** exhibited specific toxicity against Caco-2 and HeLa cells with EC50 values of 370 µM and 90 µM, respectively. Comparison of **22** and **27** modes of action revealed that they initiate different processes, leading to reduced proliferation and cell death. Considering the bioavailability parameters, the tested derivatives met the criteria for good absorption and permeation, and can be further investigated.

## Figures and Tables

**Table 1:** The structures of oxime ethers (**1**–**5**, **7**–**27**) and benzofuran oxime (**6**) and their summarized EC50 values in 24 h culture of HeLa cells.

No.	R	R^1^	R^2^	EC_50_ for HeLa Cells *	
				μg/mL	mM
1	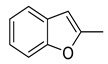	CH_3_	C_6_H_3_-2.4-diCl	>250	>0.75
2			C_6_H_4_-4-CF_3_	>250	>0.75
3			C_6_H_4_-4-Br	>250	>0.73
4			C_6_H_4_-4-NO_2_	>250	>0.81
5			C_6_H_3_-2.6-diF	>250	>0.83
6			H (oxime)	>250	>1.43
7	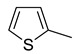	CH_3_	C_6_H_3_-2.4-diCl	>250	>0.83
8			C_6_H_4_-4-Br	188.12	0.61
9			C_6_H_4_-4-NO_2_	126.30	0.46
10			C_6_H_3_-5-MeO-2-NO_2_	>250	>0.82
11			CH_2_-NMe_2_	>250	>1.18
12			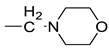	>250	>0.98
13			CH_2_-NH_2_	>250	>1.36
14			CH_2_-NEt_2_	>250	>1.04
15			CH_2_CH_2_-NH_2_	>250	>1.10
16			CH_2_CH_2_-NMe_2_	>250	>1.10
17			COOH	>250	>1.25
18	(CH_2_)_5_		COOH	32.26	0.19
19			CH_2_-NMe_2_	>250	>1.36
20			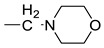	>250	>1.10
21	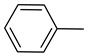	CH_3_	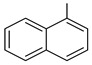	>250	>0.67
22			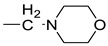	23.15	0.09
23			CH_2_-NMe_2_	233.14	1.13
24	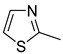	CH_3_	C_6_H_4_-4-OMe	110.30	0.42
25			C_6_H_4_-4-Br	130.10	0.42
26	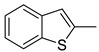	CH_3_	C_6_H_3_-2.4-diCl	121.23	0.35
27			C_6_H_4_-4-OMe	93.80	0.30

* The EC50 values were calculated using Quest Graph™ ED50 Calculator retrieved from https://www.aatbio.com/tools/ed50-calculator (accessed on 10 January 2022).

**Table 2:** Summarized EC50 values of compounds **8**, **9**, **18, 22** and **24**–**27** in 24 h and 72 h cultures of HDF, A549, Caco-2, and HeLa cells.

	EC50 Value * in μg/mL (mM)							
Cells	HDF		A549		Caco-2		HeLa	
Time No.	24 h	72 h	24 h	72 h	24 h	72 h	24 h	72 h
8	126.70 (0.41)	118.21 (0.38)	193.59 (0.62)	228.30 (0.74)	229.79 (0.74)	118.43 (0.38)	188.12 (0.61)	40.00 (0.13)
9	171.57 (0.62)	44.74 (0.16)	106.70 (0.39)	130.75 (0.47)	77.96 (0.28)	104.88 (0.38)	126.30 (0.46)	6.90 (0.02)
18	43.66 (0.26)	14.69 (0.09)	47.79 (0.28)	17.88 (0.10)	44.36 (0.26)	17.46 (0.10)	32.26 (0.19)	4.90 (0.03)
22	27.31 (0.11)	8.04 (0.03)	10.19 (0.04)	6.72 (0.03)	9.45 (0.04)	6.79 (0.03)	23.15 (0.09)	7.00 (0.03)
24	35.26 (0.13)	30.65 (0.12)	>250 (>0.95)	35.74 (0.14)	>250 (>0.95)	69.10 (0.26)	110.30 (0.42)	8.70 (0.03)
25	37.88 (0.12)	25.66 (0.08)	>250 (>0.80)	79.86 (0.26)	70.85 (0.23)	50.47 (0.16)	130.10 (0.42)	47.33 (0.15)
26	134.78 (0.38)	25.66 (0.07)	199.12 (0.57)	176.95 (0.51)	>250 (>0.71)	50.08 (0.14)	121.23 (0.35)	8.18 (0.02)
27	149.64 (0.48)	139.57 (0.45)	>250 (>0.80)	142.64 (0.46)	122.00 (0.39)	116.47 (0.37)	93.80 (0.30)	27.9 (0.09)

* The EC50 values were calculated using Quest Graph™ ED50 Calculator retrieved from https://www.aatbio.com/tools/ed50-calculator (accessed on 10 January 2022).

**Table 3:** Physicochemical properties of oxime ethers **1**–**5**, **7**–**27** and oxime **6**.

No.	miLog P ^a^	tPSA [Å^2^] ^a^	MW ^a^	nOH ^a^	nOHNH ^a^	Nrotb ^a^	Lipinski Violations ^a^	Veber Violations ^a^
1	5.62	34.74	334.20	3	0	4	1	0
2	5.23	37.74	333.31	3	0	5	1	0
3	5.15	34.74	344.21	3	0	4	1	0
4	4.30	80.56	310.31	6	0	5	0	0
5	4.57	34.74	301.29	3	0	4	0	0
6	2.47	45.73	175.19	3	1	1	0	0
7	4.96	21.60	300.21	2	0	4	0	0
8	4.48	21.60	310.22	2	0	4	0	0
9	3.63	67.42	276.32	5	0	5	0	0
10	3.62	76.66	306.34	6	0	6	0	0
11	2.11	24.84	212.32	3	0	5	0	0
12	1.95	34.07	254.35	4	0	5	0	0
13	0.88	47.62	184.26	3	2	4	0	0
14	2.86	24.84	240.37	3	0	7	0	0
15	1.15	47.62	198.29	3	2	5	0	0
16	2.38	24.84	226.34	3	0	6	0	0
17	1.32	58.90	199.23	4	1	4	0	0
18	1.37	58.90	171.20	4	1	3	0	0
19	2.16	24.84	184.28	3	0	4	0	0
20	2.00	34.07	226.32	4	0	4	0	0
21	4.93	21.60	375.35	2	0	4	0	0
22	2.05	34.07	248.33	4	0	5	0	0
23	2.21	24.84	206.27	3	0	5	0	0
24	2.49	43.72	262.33	4	0	5	0	0
25	3.25	34.49	311.20	3	0	4	0	0
26	6.26	21.60	350.27	2	0	4	1	0
27	5.04	30.83	311.41	3	0	5	1	0

^a^ Molinspiration; abbreviations: MW, molecular mass; tPSA, topological polar surface area; Nrotb, number of rotatable bonds.

## Supplementary Materials

The following supporting information can be downloaded at. Figure S1: The cytotoxicity evaluation on HeLa cell culture after 24 h of exposure to the compounds **1**–**27**. Figure S2: Spectral data of the new synthesized compounds **13**–**15** and **24**–**27**. Figure S3: NMR Spectra of the other compounds.

## Sample Availability

Samples of the compounds **1**–**27** are available from the authors.
