# Supplementary material for: The Oxime Ethers with Heterocyclic, Alicyclic and Aromatic Moiety as Potential Anti-Cancer Agents
Source: Molecules. 2022 Feb 17;27(4):1374. doi: 10.3390/molecules27041374 (PMC8878717; doi:10.3390/molecules27041374)
Supplement: Supplementary file 1 [file molecules-27-01374-s001.zip › molecules-1508509-supplementary.pdf]

Supplementary Information to:

The oxime ethers with heterocyclic, alicyclic and aromatic moiety as potential anti-cancer agents

Tomasz Kosmalski [1,*], Anna Hetmann [2], Renata Studzińska [1,*], Szymon Baumgart [1], Daria Kupczyk [3] and Katarzyna Roszek [2]

[1] Nicolaus Copernicus University in Toruń, Collegium Medicum in Bydgoszcz, Faculty of Pharmacy, Department of Organic Chemistry, Jurasza 2, 85–089 Bydgoszcz, Poland; tkosm@cm.umk.pl
[2] Faculty of Biological and Veterinary Sciences, Institute of Biology, Department of Biochemistry, Lwowska 1, 87-100 Torun, Poland;
[3] Nicolaus Copernicus University in Toruń, Collegium Medicum in Bydgoszcz, Faculty of Medicine, Department of Medical Biology and Biochemistry, Karłowicza 24, 85–092 Bydgoszcz, Poland; dariak@cm.umk.pl

S1. The cytotoxicity evaluation on HeLa cell culture after 24h of exposure to the compounds **1-27**.

A/

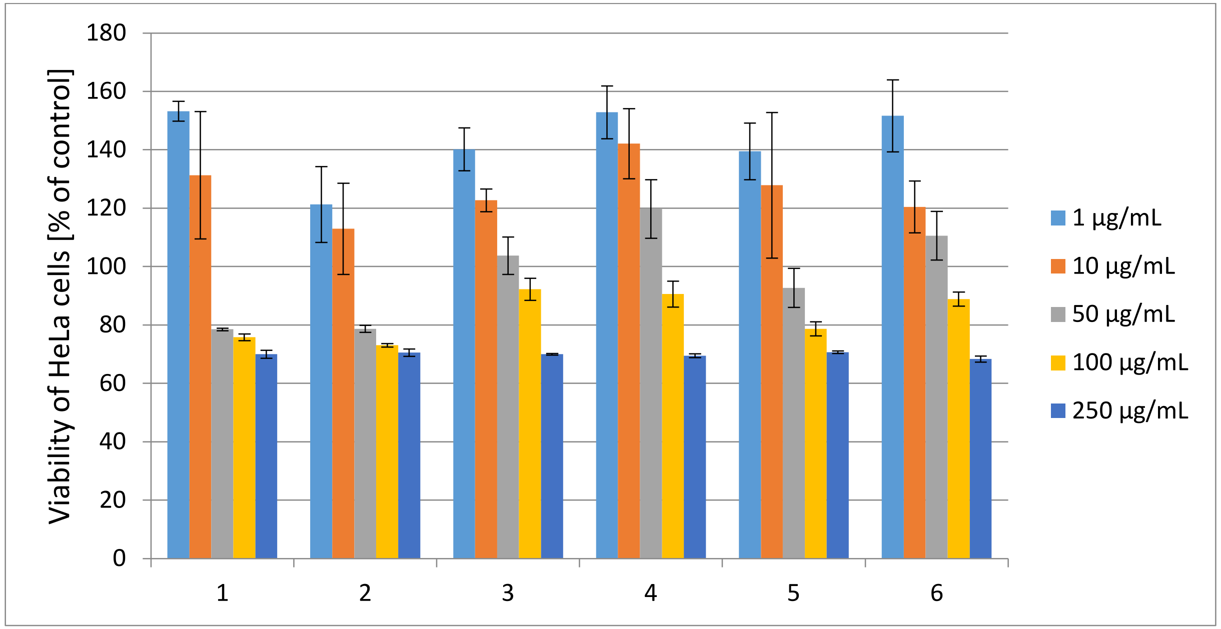

B/

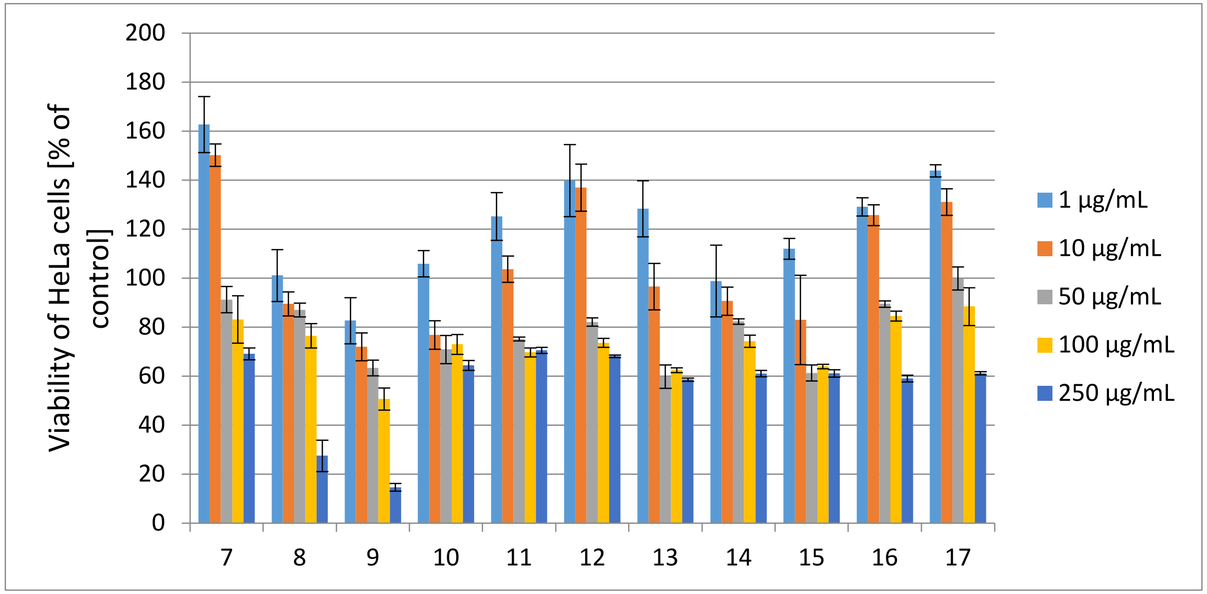

Viability of HeLa cells [% of control]
200
180
160
140
120
100
80
60
40
20
0
7
8
9
10
11
12
13
14
15
16
17
1 µg/mL
10 µg/mL
50 µg/mL
100 µg/mL
250 µg/mL

c/

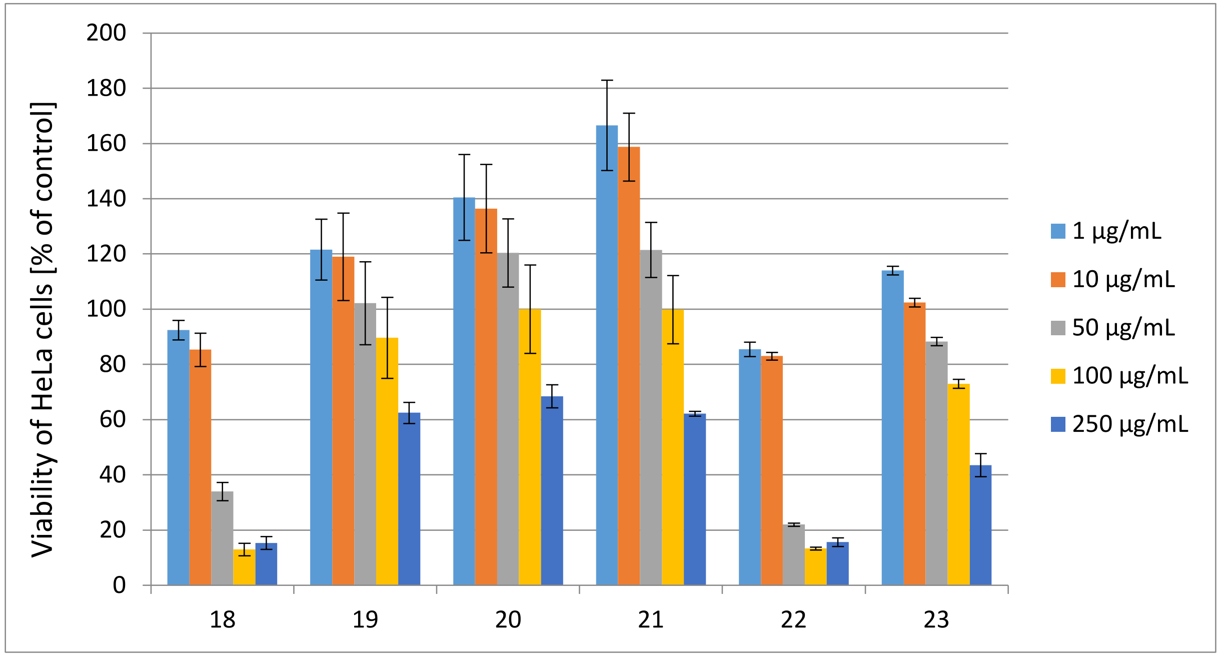

Viability of HeLa cells [% of control]
200
180
160
140
120
100
80
60
40
20
0
18
19
20
21
22
23
1 µg/mL
10 µg/mL
50 µg/mL
100 µg/mL
250 µg/mL

D/

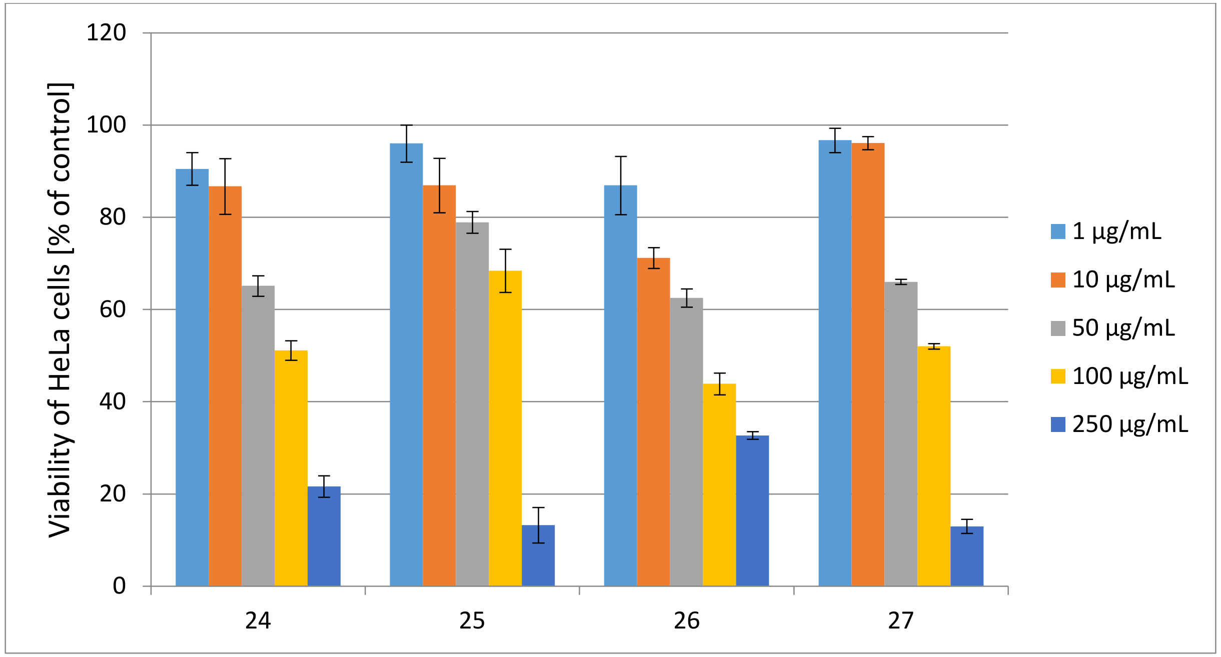

Figure S1. The cytotoxicity evaluation on HeLa cell culture after 24h of exposure to: A/ benzofuran oxime ethers (**1-5**) and 2-acetylbenzofuran oxime (**6**), B/ thiophene oxime ethers (**7-17**), C/ cyclohexanone and acetophenone oxime ethers (**18-23**), D/ thiazole and benzothiophene oxime ethers (**24-27**).

S2. Spectral data of the new synthesized compounds **13-15** and **24-27**.

Spectral data of the compound **13.**

^{1}H NMR spectrum of the compound **13**

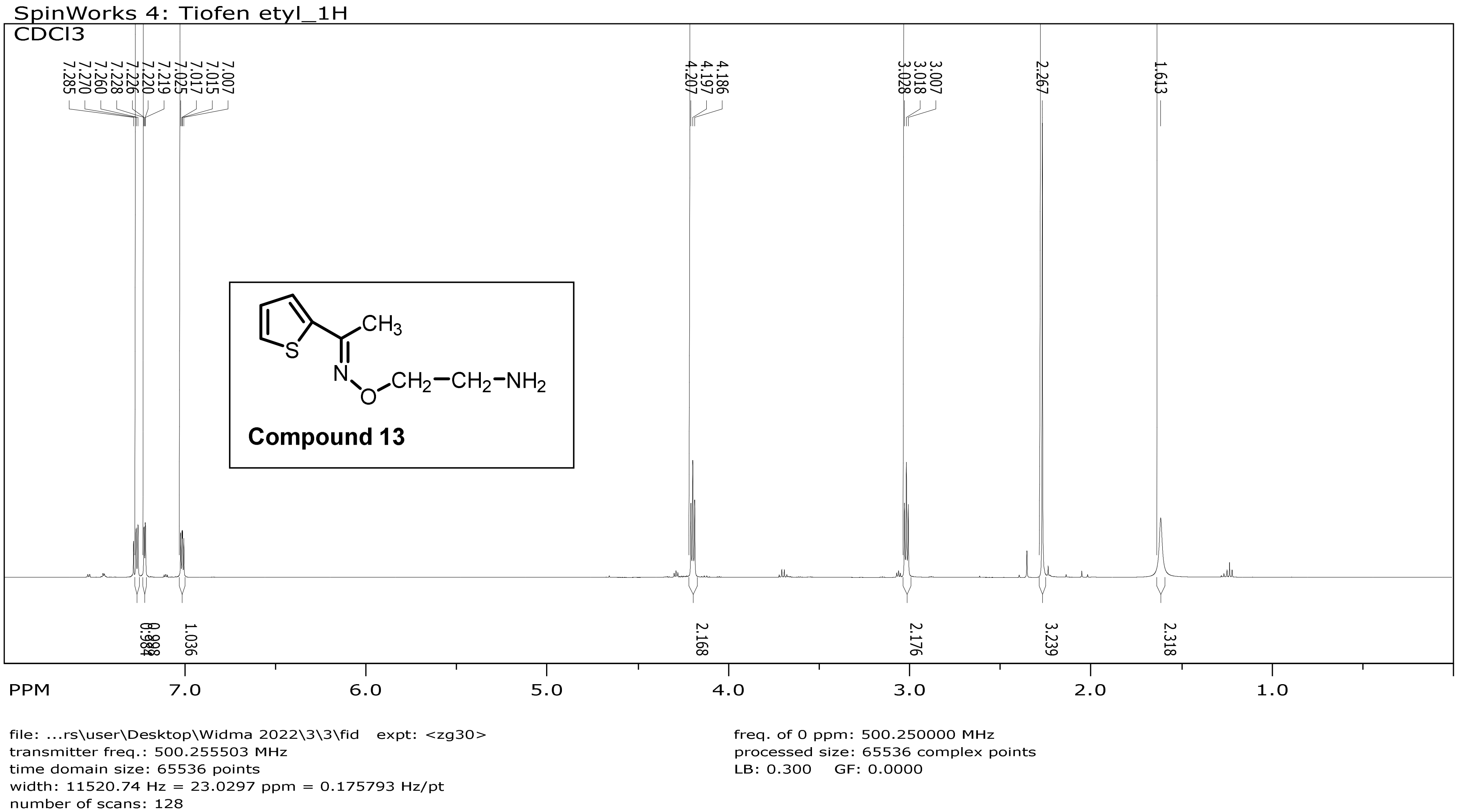

^{13}C NMR spectrum of the compound **13**

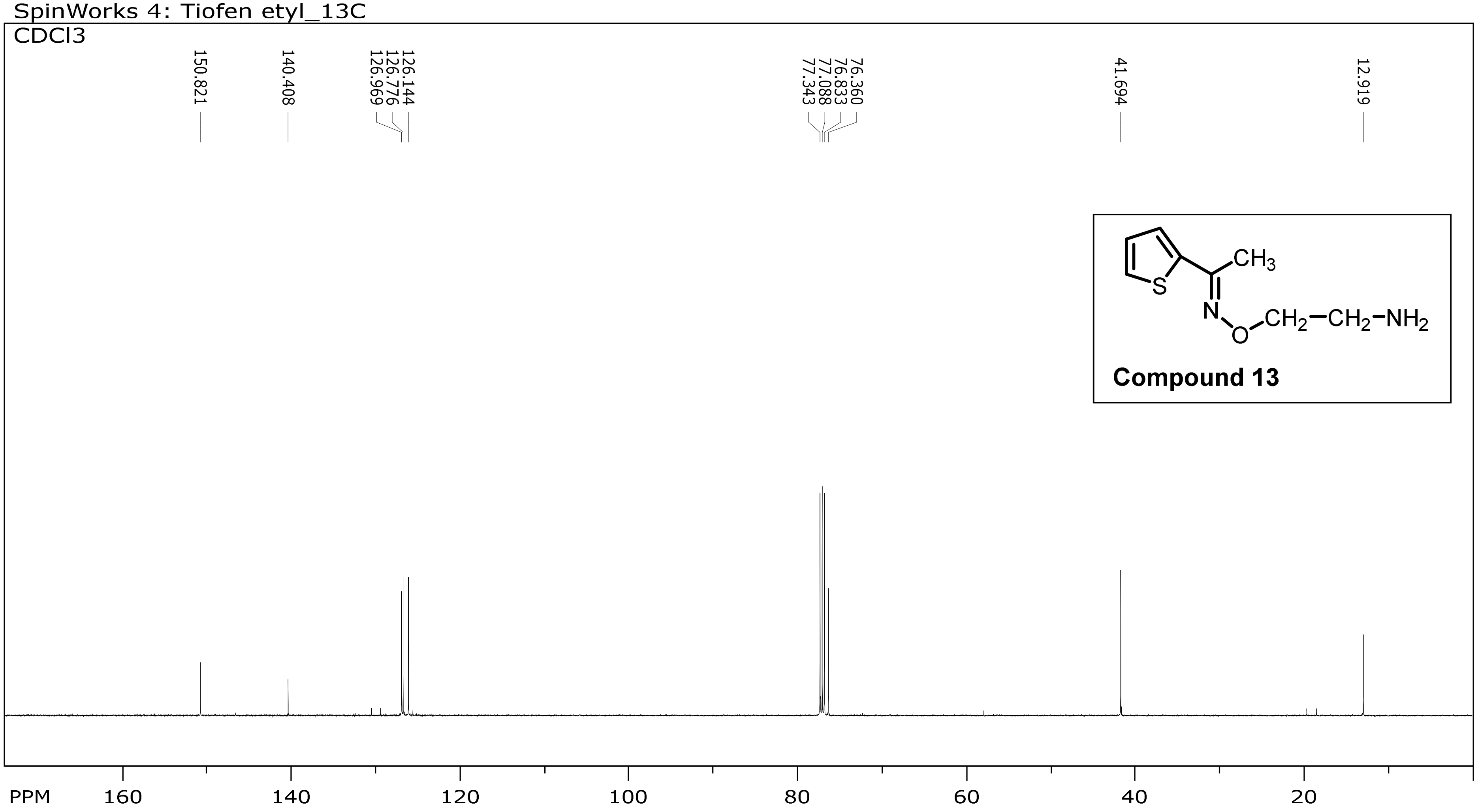

file: ...rs\user\Desktop\Widma 2022\4\4\fid expt: <zgpg30>
transmitter freq.: 125.803057 MHz
time domain size: 65536 points
width: 32894.74 Hz = 261.4780 ppm = 0.501934 Hz/pt
number of scans: 1024

freq. of 0 ppm: 125.787963 MHz
processed size: 32768 complex points
LB: 1.000 GF: 0.0000

Mass spectrum of the compound **13**

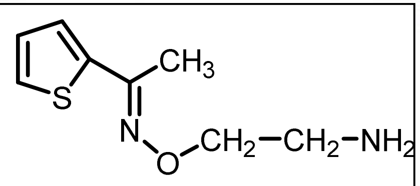

Compound 13

Elemental Composition Report

Single Mass Analysis
Tolerance = 5.0 PPM / DBE: min = -1.5, max = 70.0
Element prediction: Off
Number of isotope peaks used for i-FIT = 9

Monoisotopic Mass, Even Electron Ions
302 formula(e) evaluated with 1 results within limits (all results (up to 1000) for each mass)
Elements Used:
C: 3-15 H: 0-25 N: 0-10 O: 0-10 S: 0-2
201009_Tiofen_etyl_NH2_A 42 (0.445) Cm (42:45)

TOF MS ES+
1.39e+006

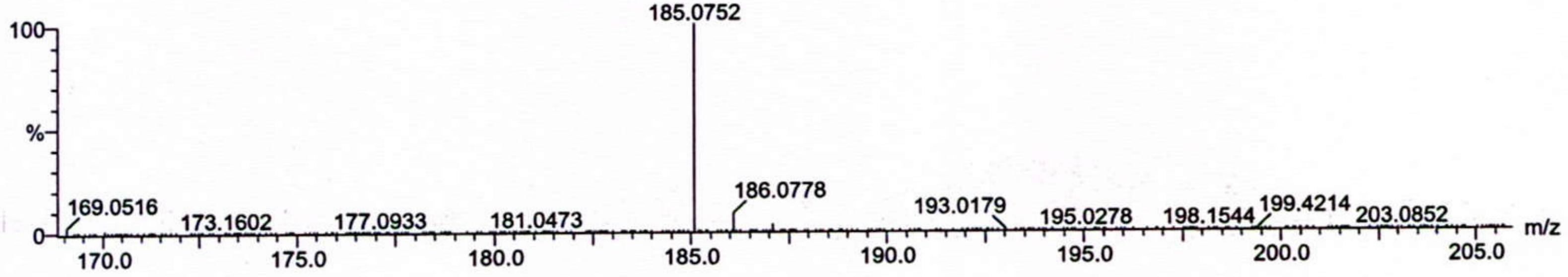

Mass	Calc. Mass	mDa	PPM	DBE	i-FIT	Norm	Conf(%)	Formula
Minimum:				-1.5				
Maximum:		5.0	5.0	70.0				
185.0752	185.0749	0.3	1.6	3.5	618.4	n/a	n/a	C8 H13 N2 O S

Spectral data of the compound **14.**

^{1}H NMR spectrum of the compound **14**

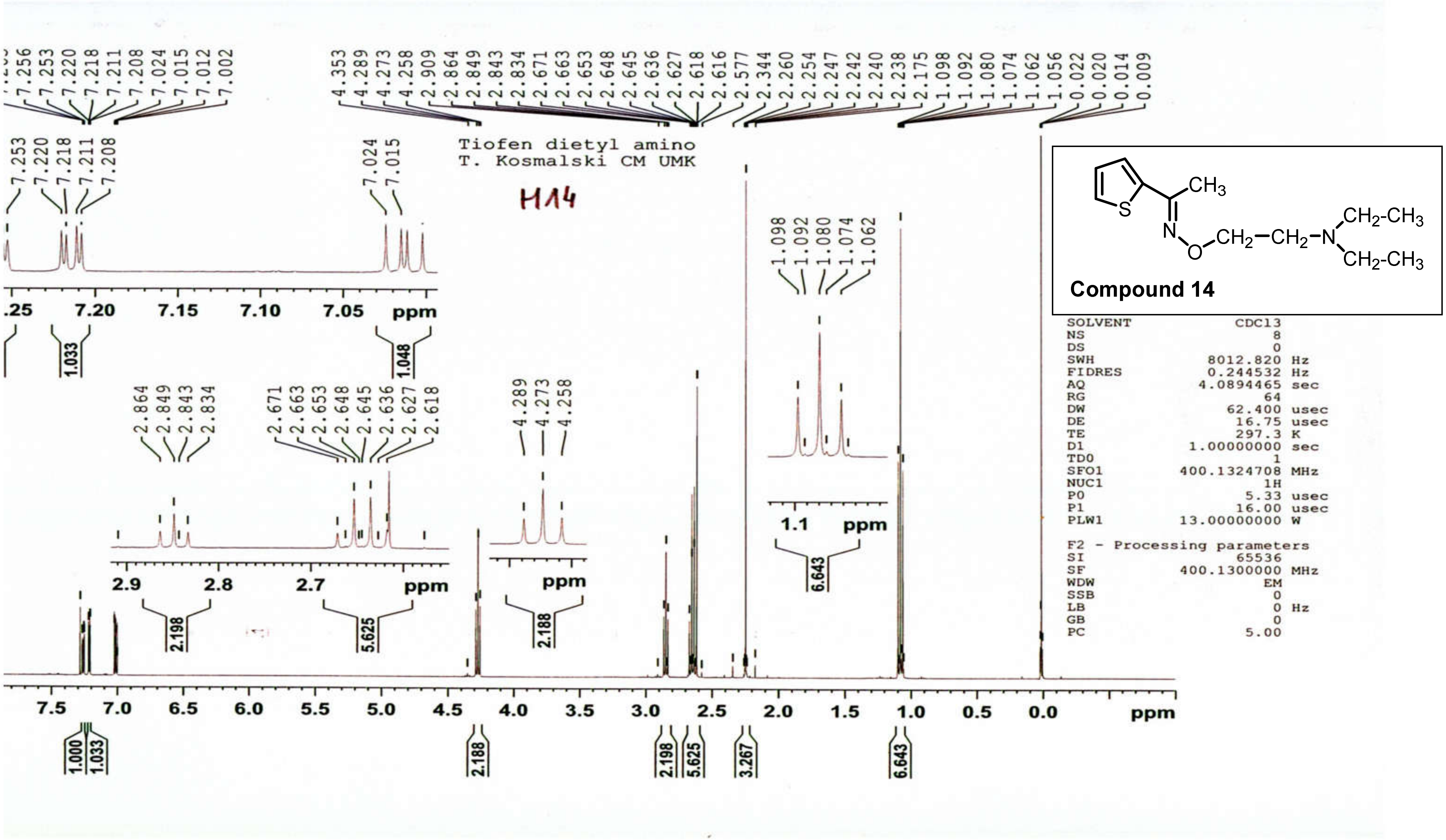

^{13}C NMR spectrum of the compound **14**

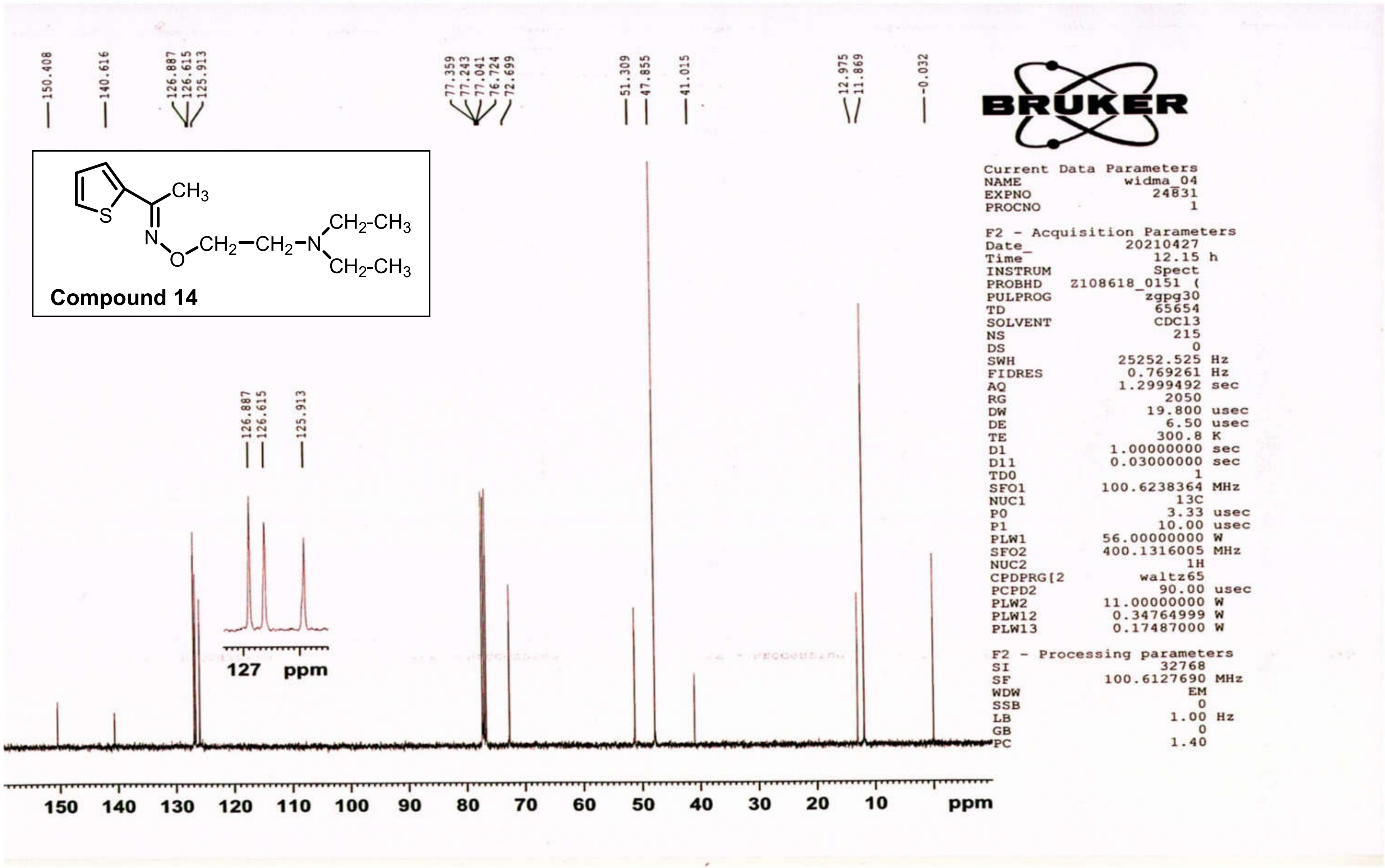

MS spectrum of the compound **14**

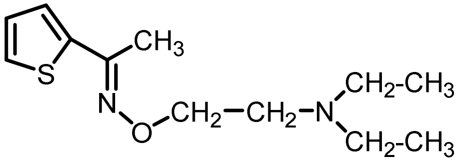

Compound 14

Elemental Composition Report

Single Mass Analysis

Tolerance = 5.0 PPM / DBE: min = -1.5, max = 80.0
Element prediction: Off
Number of isotope peaks used for i-FIT = 9

Monoisotopic Mass, Even Electron Ions
316 formula(e) evaluated with 1 results within limits (all results (up to 1000) for each mass)
Elements Used:
C: 0-30 H: 0-50 N: 0-3 O: 0-10 S: 0-2
210427_Tiofen_dietylA 27 (0.294) Cm (27:30)

TOF MS ES+
9.07e+006

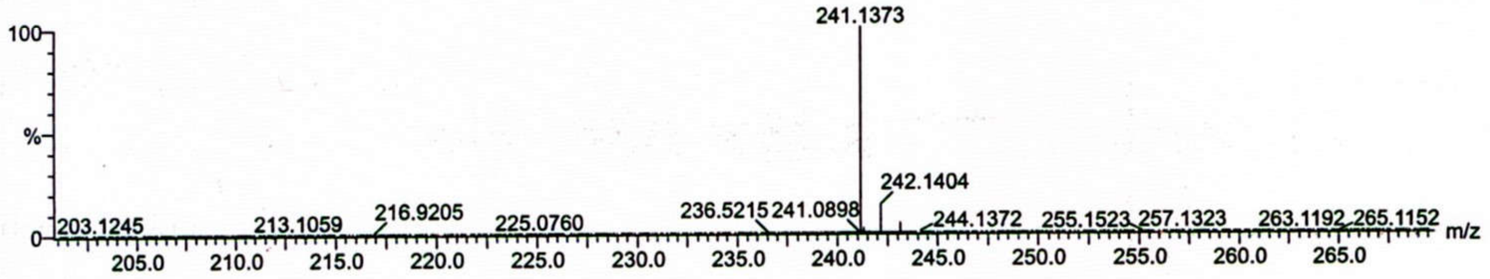

	Mass	Calc. Mass	mDa	PPM	DBE	i-FIT	Norm	Conf(%)	Formula
Minimum:					-1.5				
Maximum:			5.0	5.0	80.0				
	241.1373	241.1375	-0.2	-0.8	3.5	1254.5	n/a	n/a	C12 H21 N2 O S

Spectral data of the compound **15.**

^{1}H NMR spectrum of the compound **15**

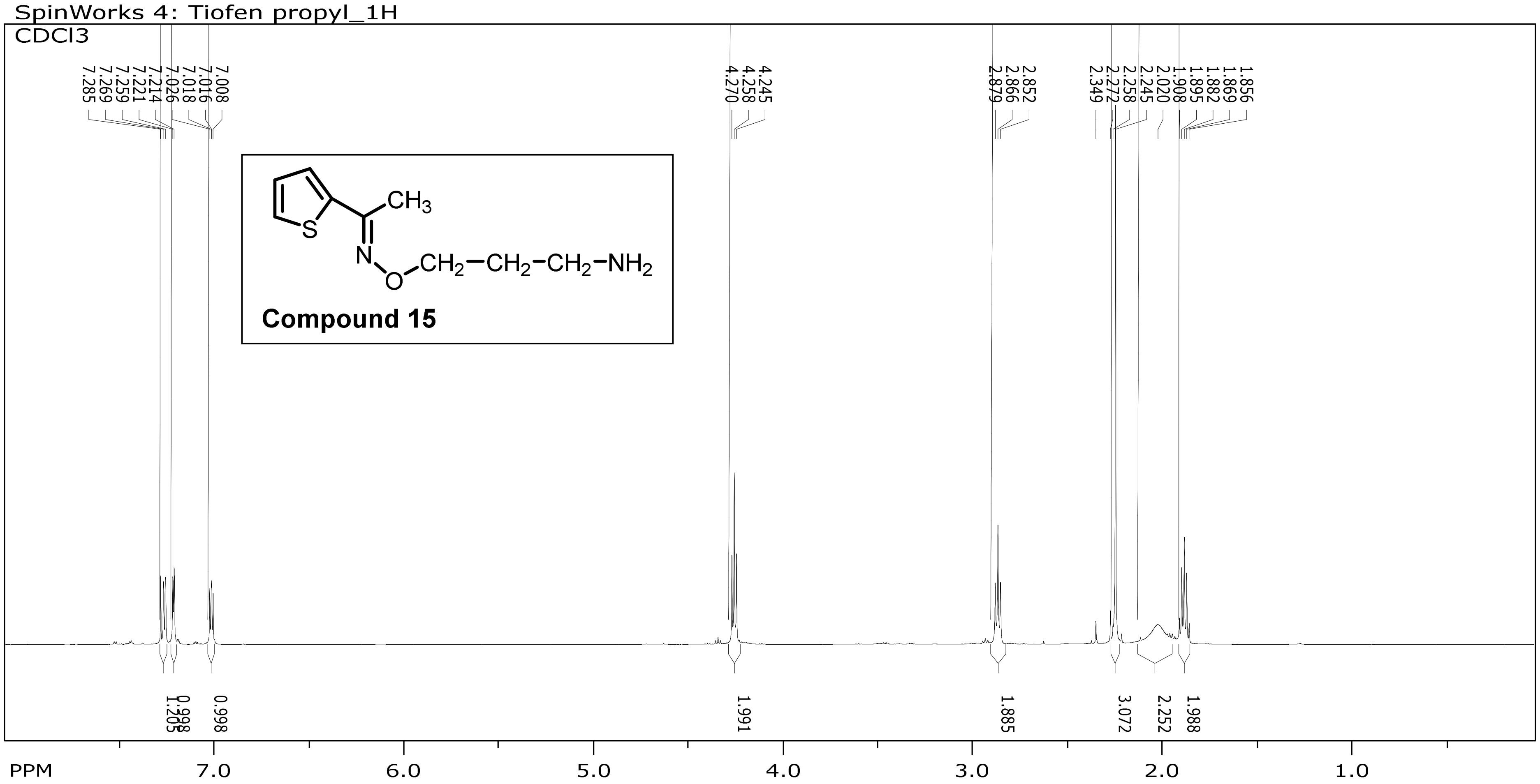

file: ...rs\user\Desktop\Widma 2022\5\5\fid expt: <zg30>
transmitter freq.: 500.255503 MHz
time domain size: 65536 points
width: 11520.74 Hz = 23.0297 ppm = 0.175793 Hz/pt
number of scans: 128

freq. of 0 ppm: 500.250000 MHz
processed size: 65536 complex points
LB: 0.300 GF: 0.0000

^{13}C NMR spectrum of the compound **15**

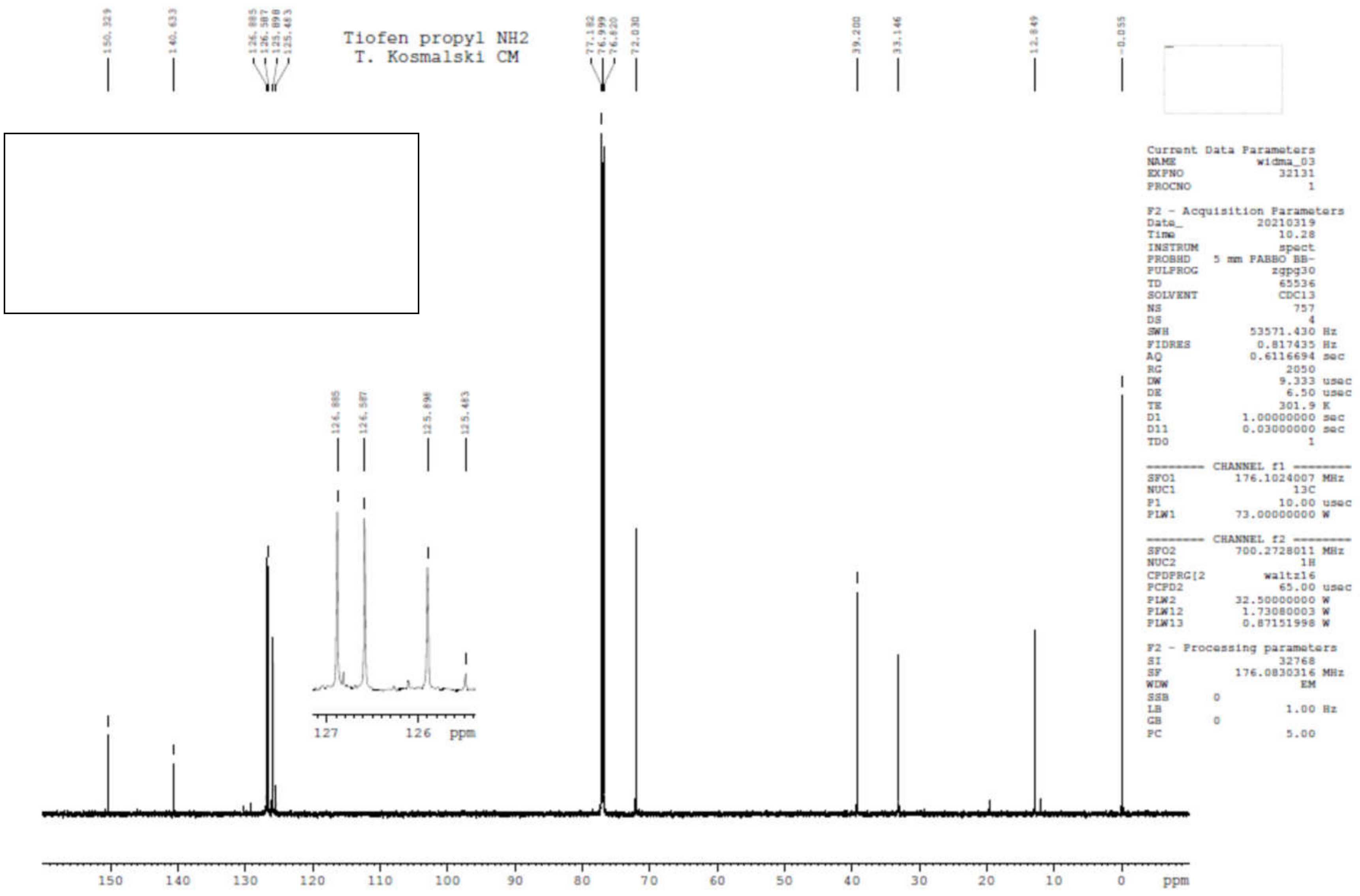

MS spectrum of the compound **15**

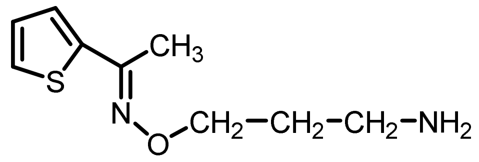

Compound 15

Elemental Composition Report

Single Mass Analysis

Tolerance = 5.0 PPM / DBE: min = -1.5, max = 70.0
Element prediction: Off
Number of isotope peaks used for i-FIT = 9

Monoisotopic Mass, Even Electron Ions
384 formula(e) evaluated with 1 results within limits (all results (up to 1000) for each mass)
Elements Used:
C: 3-40 H: 0-40 N: 0-10 O: 0-10 S: 0-2
201009_Tiofen_propyl_NH2_A 31 (0.328) Cm (31:39-4:8)

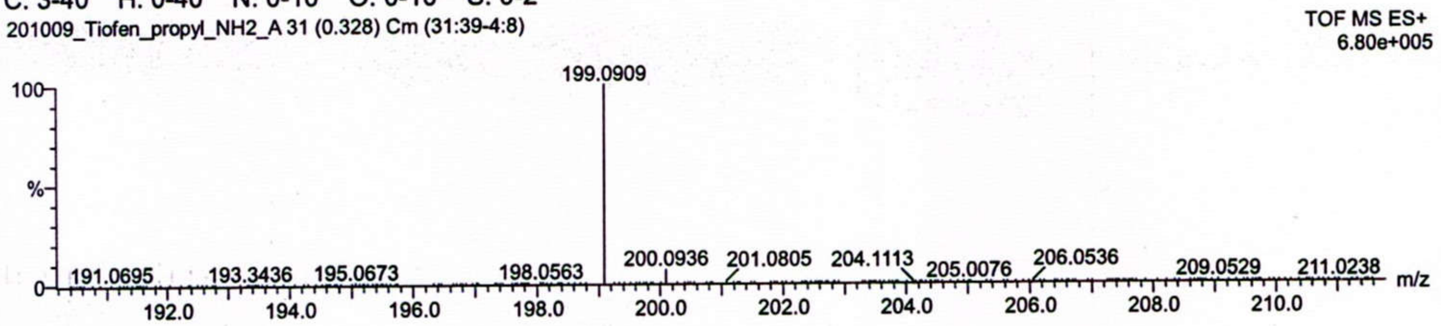

	Mass	Calc. Mass	mDa	PPM	DBE	i-FIT	Norm	Conf(%)	Formula
Minimum:					-1.5				
Maximum:			5.0	5.0	70.0				
	199.0909	199.0905	0.4	2.0	3.5	841.4	n/a	n/a	C9 H15 N2 O S

Spectral data of the compound **24.**

^{1}H NMR spectrum of the compound **24**

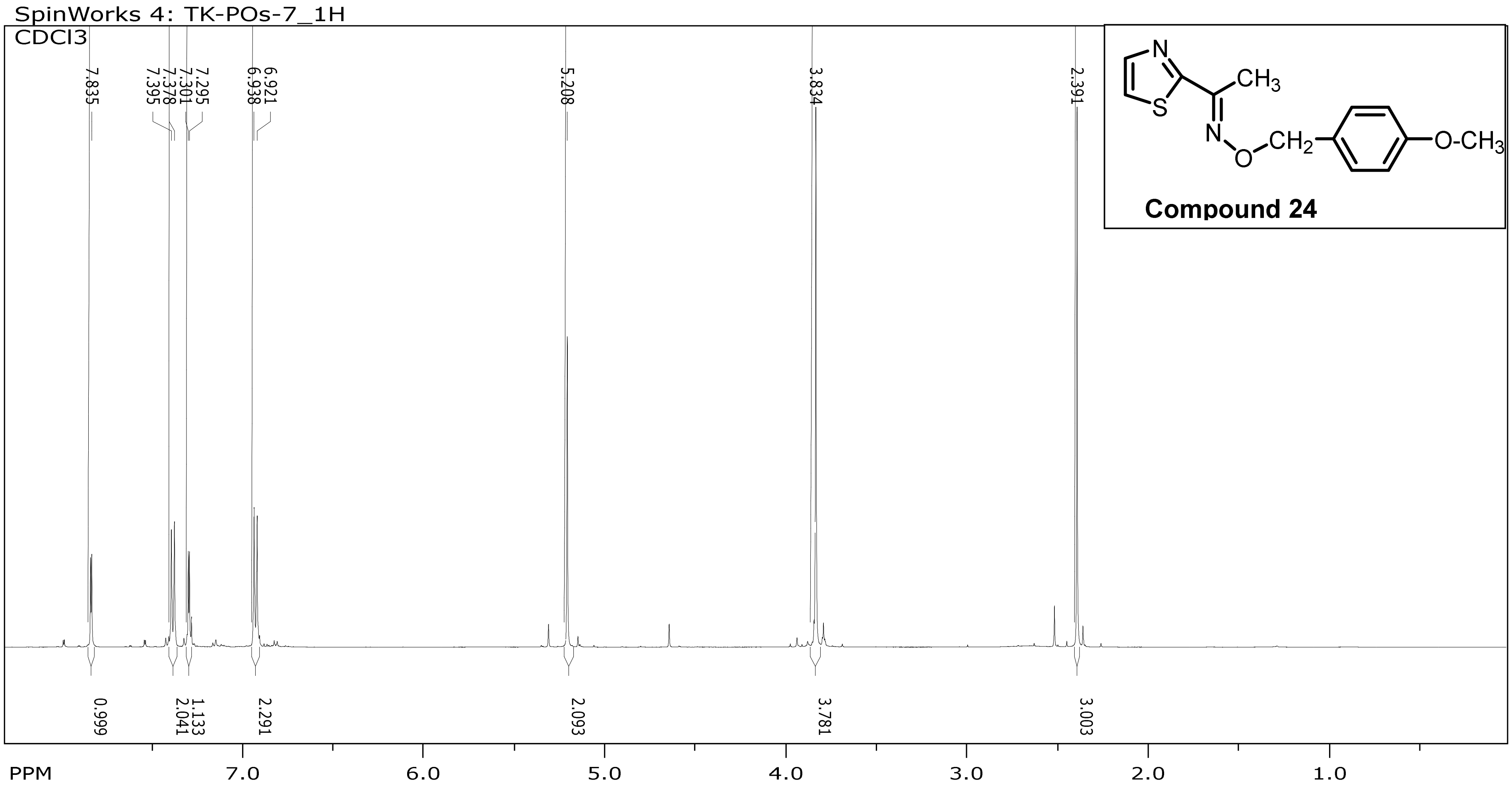

file: ...ers\user\Desktop\Widma 2021\35\fid expt: <zg30>
transmitter freq.: 500.255003 MHz
time domain size: 65536 points
width: 11029.41 Hz = 22.0476 ppm = 0.168295 Hz/pt
number of scans: 128

freq. of 0 ppm: 500.250000 MHz
processed size: 65536 complex points
LB: 0.300 GF: 0.0000

^{13}C NMR spectrum of the compound **24**

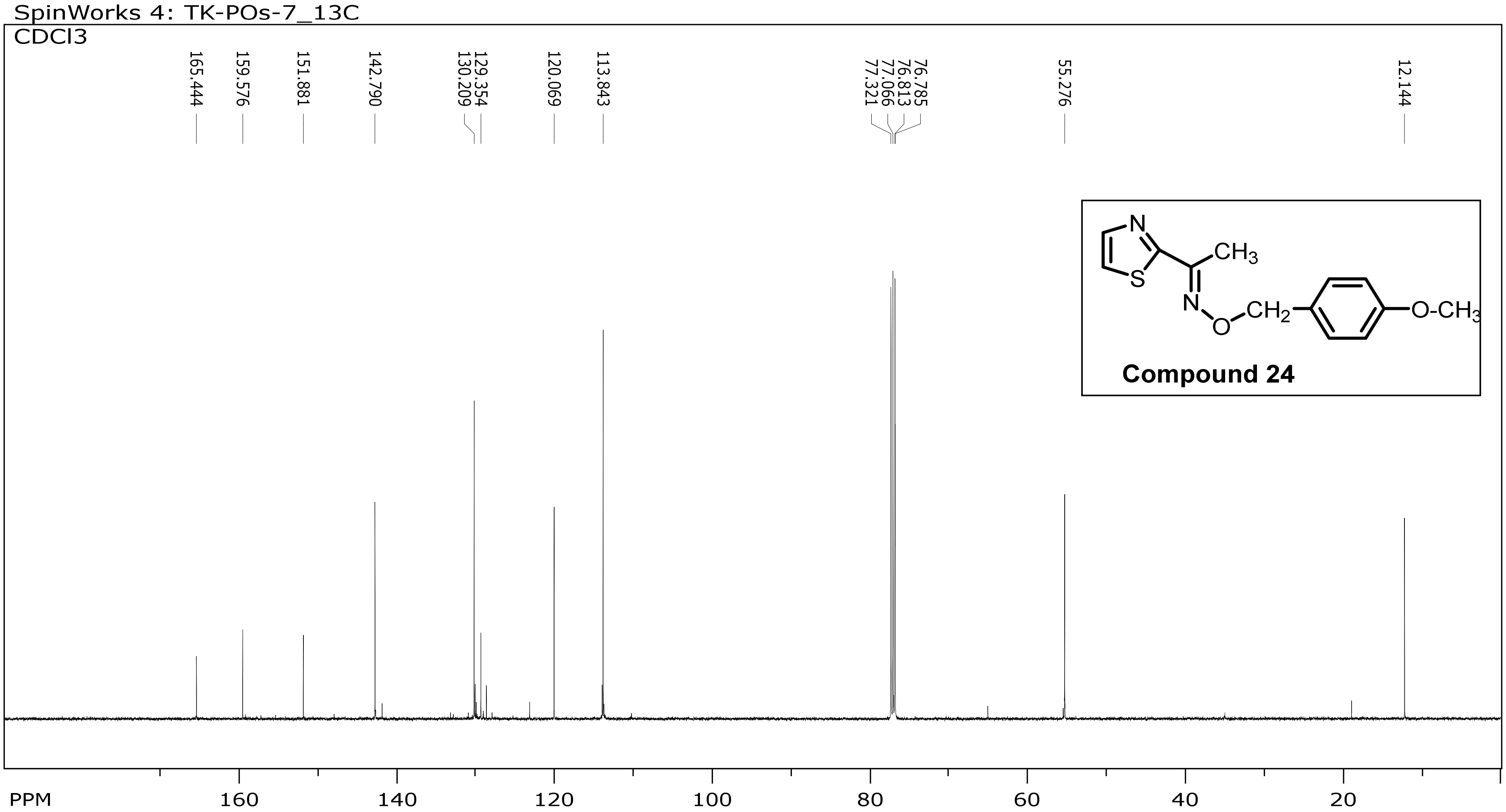

file: ...ers\user\Desktop\Widma 2021\36\fid expt: <zgpg30>
transmitter freq.: 125.803057 MHz
time domain size: 65536 points
width: 31250.00 Hz = 248.4041 ppm = 0.476837 Hz/pt
number of scans: 1024

freq. of 0 ppm: 125.787963 MHz
processed size: 32768 complex points
LB: 1.000 GF: 0.0000

MS spectrum of the compound **24**

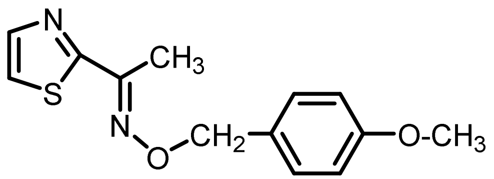

Compound 24

Elemental Composition Report

Single Mass Analysis

Tolerance = 5.0 PPM / DBE: min = -1.5, max = 80.0
Element prediction: Off
Number of isotope peaks used for i-FIT = 9

Monoisotopic Mass, Even Electron Ions
96 formula(e) evaluated with 1 results within limits (all results (up to 1000) for each mass)
Elements Used:
C: 0-30 H: 0-30 N: 0-2 O: 0-5 S: 1-2
210901_Tiazol_4_OMeA 20 (0.223) Cm (20:25-4:8)

TOF MS ES+
6.54e+005

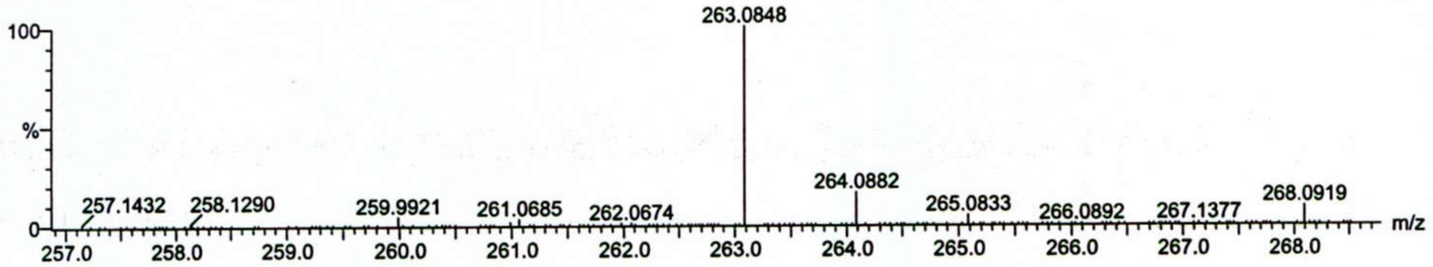

Minimum:				-1.5				
Maximum:		5.0	5.0	80.0				
Mass	Calc. Mass	mDa	PPM	DBE	i-FIT	Norm	Conf(%)	Formula
263.0848	263.0854	-0.6	-2.3	7.5	787.9	n/a	n/a	C13 H15 N2 O2 S

Spectral data of the compound **25.**

^{1}H NMR spectrum of the compound **25**

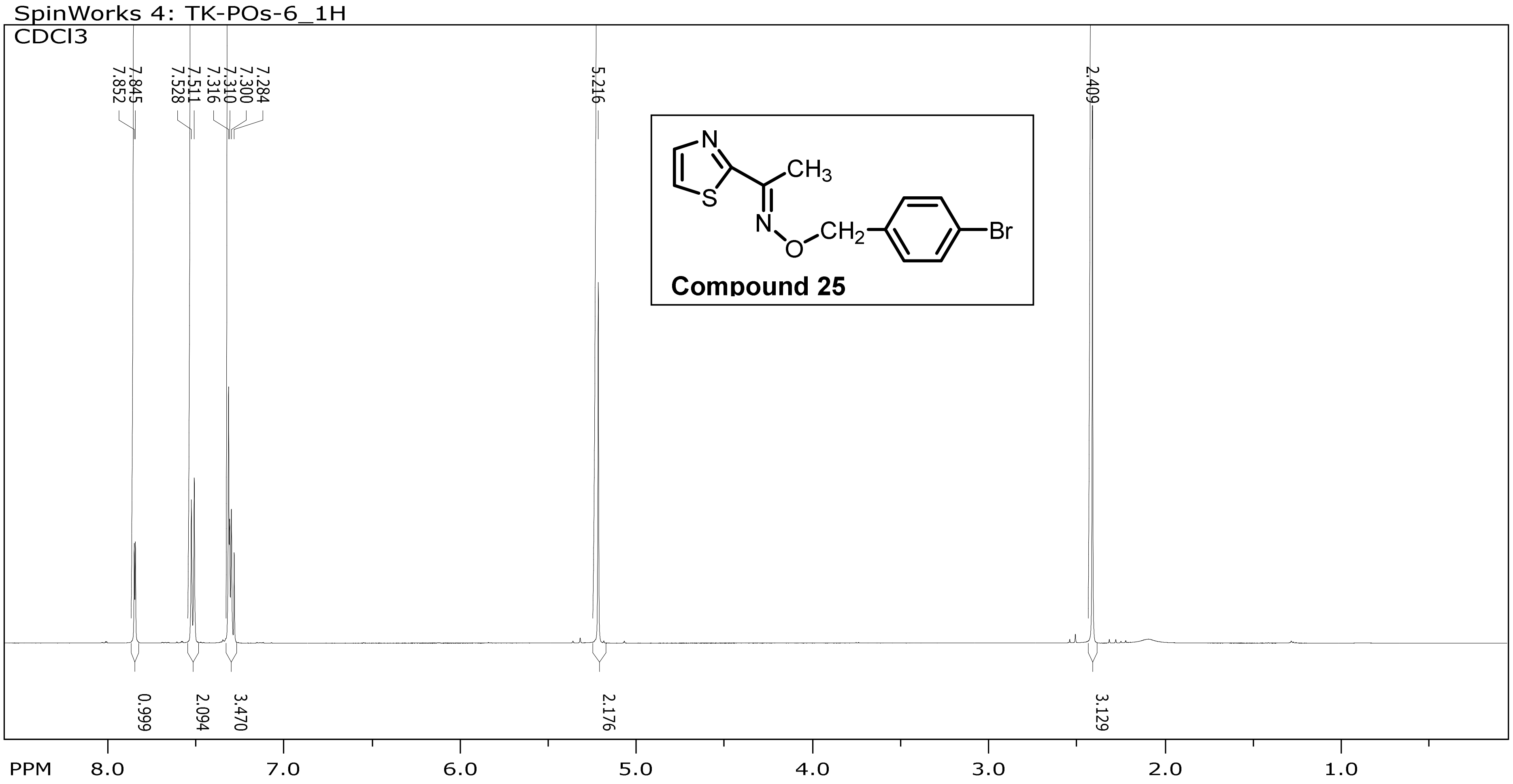

file: ...ers\user\Desktop\Widma 2021\33\fid expt: <zg30>
transmitter freq.: 500.255003 MHz
time domain size: 65536 points
width: 11029.41 Hz = 22.0476 ppm = 0.168295 Hz/pt
number of scans: 128

freq. of 0 ppm: 500.250000 MHz
processed size: 65536 complex points
LB: 0.300 GF: 0.0000

^{13}C NMR spectrum of the compound **25**

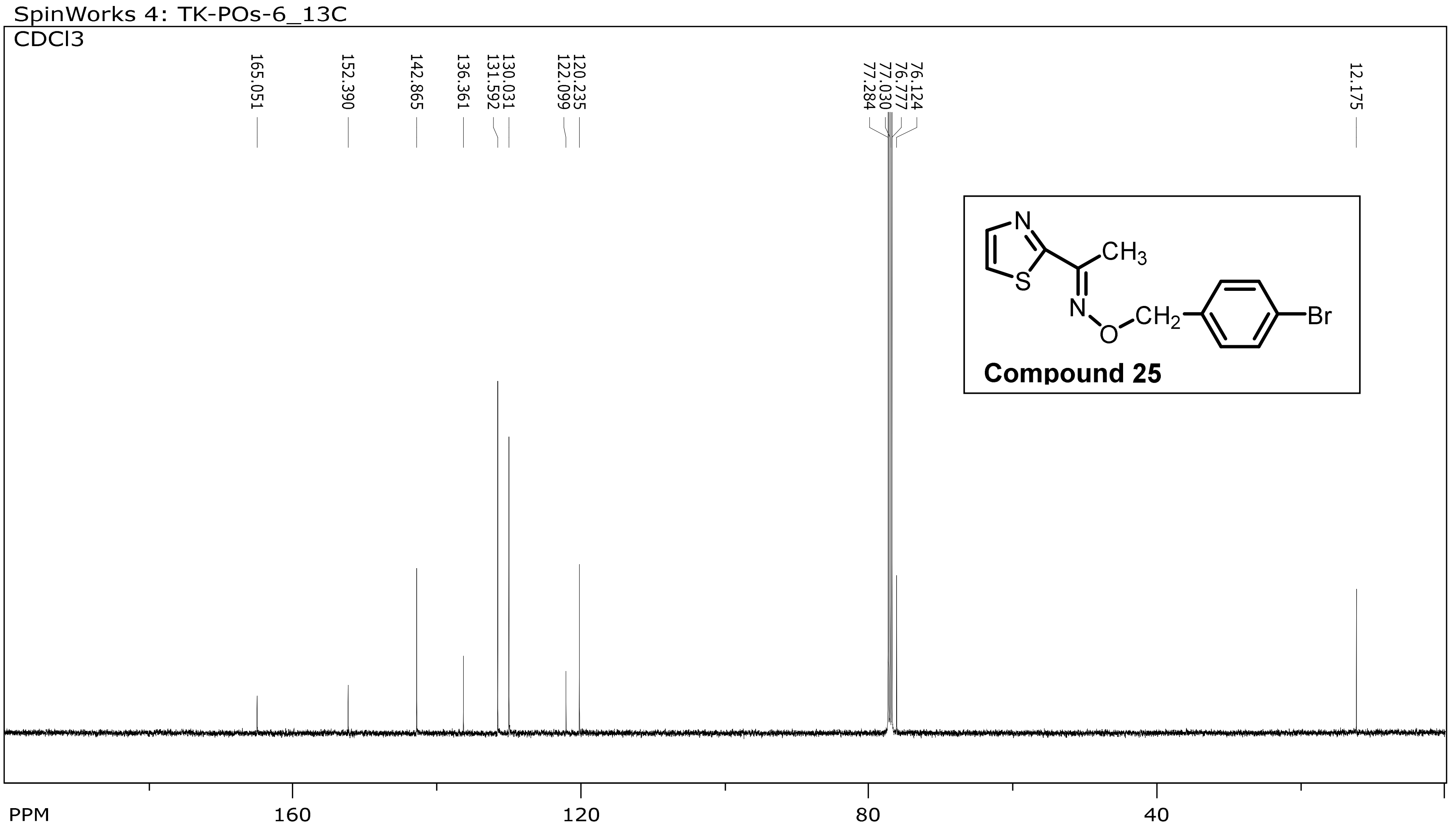

file: ...ers\user\Desktop\Widma 2021\34\fid expt: <zgpg30>
transmitter freq.: 125.803057 MHz
time domain size: 65536 points
width: 31250.00 Hz = 248.4041 ppm = 0.476837 Hz/pt
number of scans: 1024

freq. of 0 ppm: 125.787963 MHz
processed size: 32768 complex points
LB: 1.000 GF: 0.0000

MS spectrum of the compound **25**

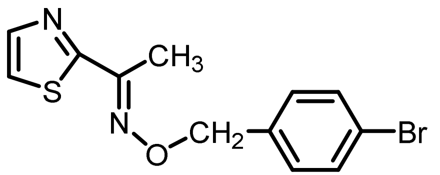

Compound 25

Elemental Composition Report

Single Mass Analysis

Tolerance = 5.0 PPM / DBE: min = -1.5, max = 80.0
Element prediction: Off
Number of isotope peaks used for i-FIT = 9

Monoisotopic Mass, Even Electron Ions
187 formula(e) evaluated with 1 results within limits (all results (up to 1000) for each mass)
Elements Used:
C: 0-30 H: 0-30 N: 0-2 O: 0-5 S: 1-2 Br: 0-1
210901_Tiazol_BrA 43 (0.454) Cm (34:46-3:8)

TOF MS ES+
8.04e+006

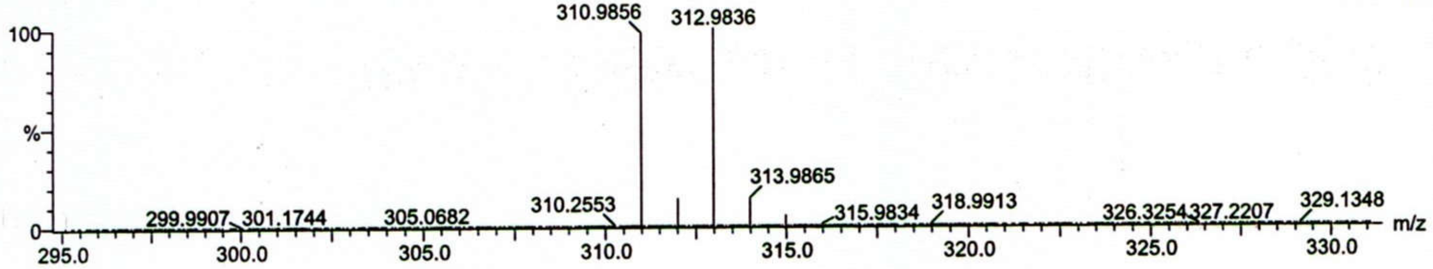

Minimum:				-1.5				
Maximum:		5.0	5.0	80.0				
Mass	Calc. Mass	mDa	PPM	DBE	i-FIT	Norm	Conf(%)	Formula
310.9856	310.9854	0.2	0.6	7.5	1966.8	n/a	n/a	C12 H12 N2 O S Br

Spectral data of the compound **26.**

^{1}H NMR spectrum of the compound **26**

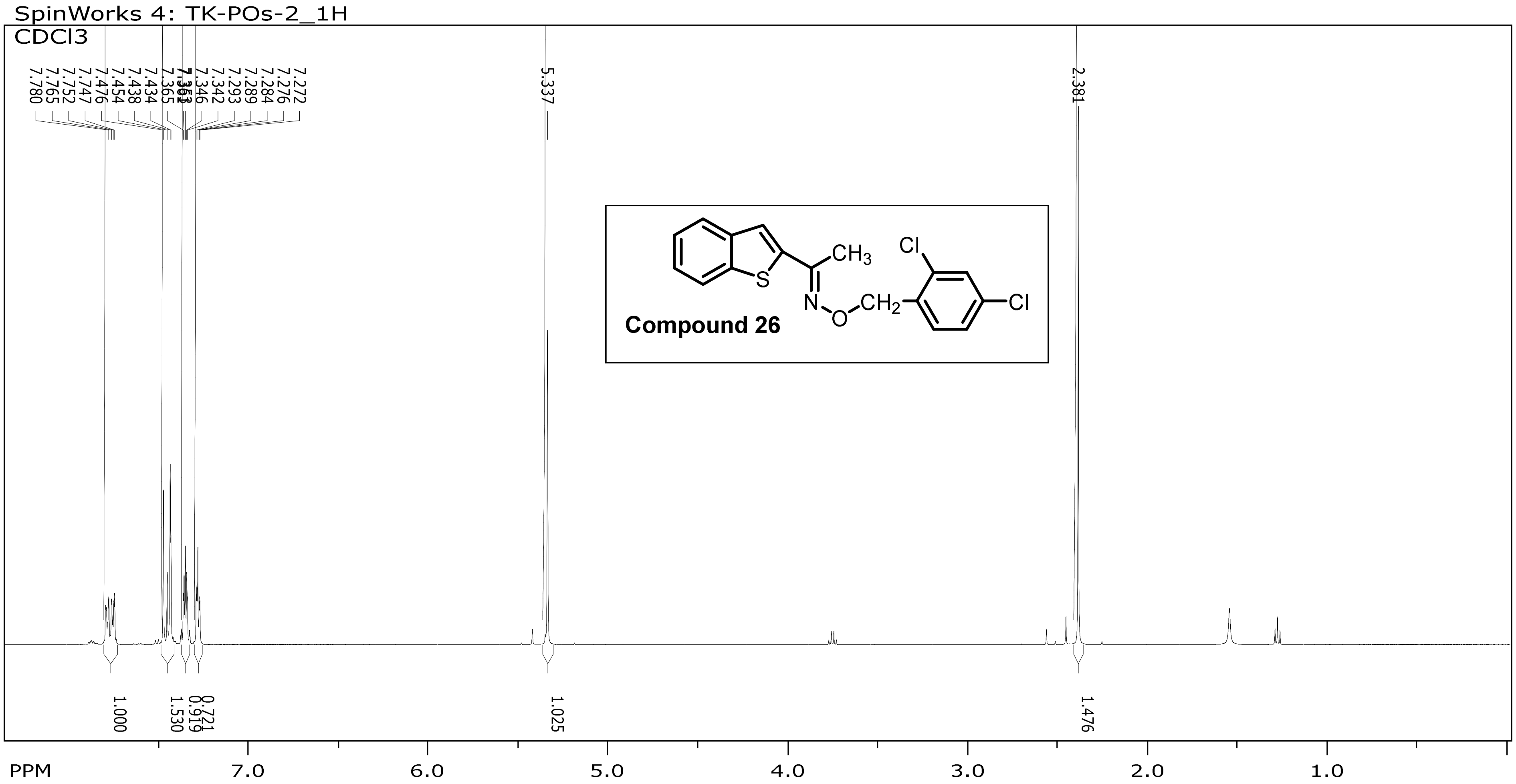

file: ...ers\user\Desktop\Widma 2021\29\fid expt: <zg30>
transmitter freq.: 500.255003 MHz
time domain size: 65536 points
width: 11029.41 Hz = 22.0476 ppm = 0.168295 Hz/pt
number of scans: 128

freq. of 0 ppm: 500.250000 MHz
processed size: 65536 complex points
LB: 0.300 GF: 0.0000

^{13}C NMR spectrum of the compound **26**

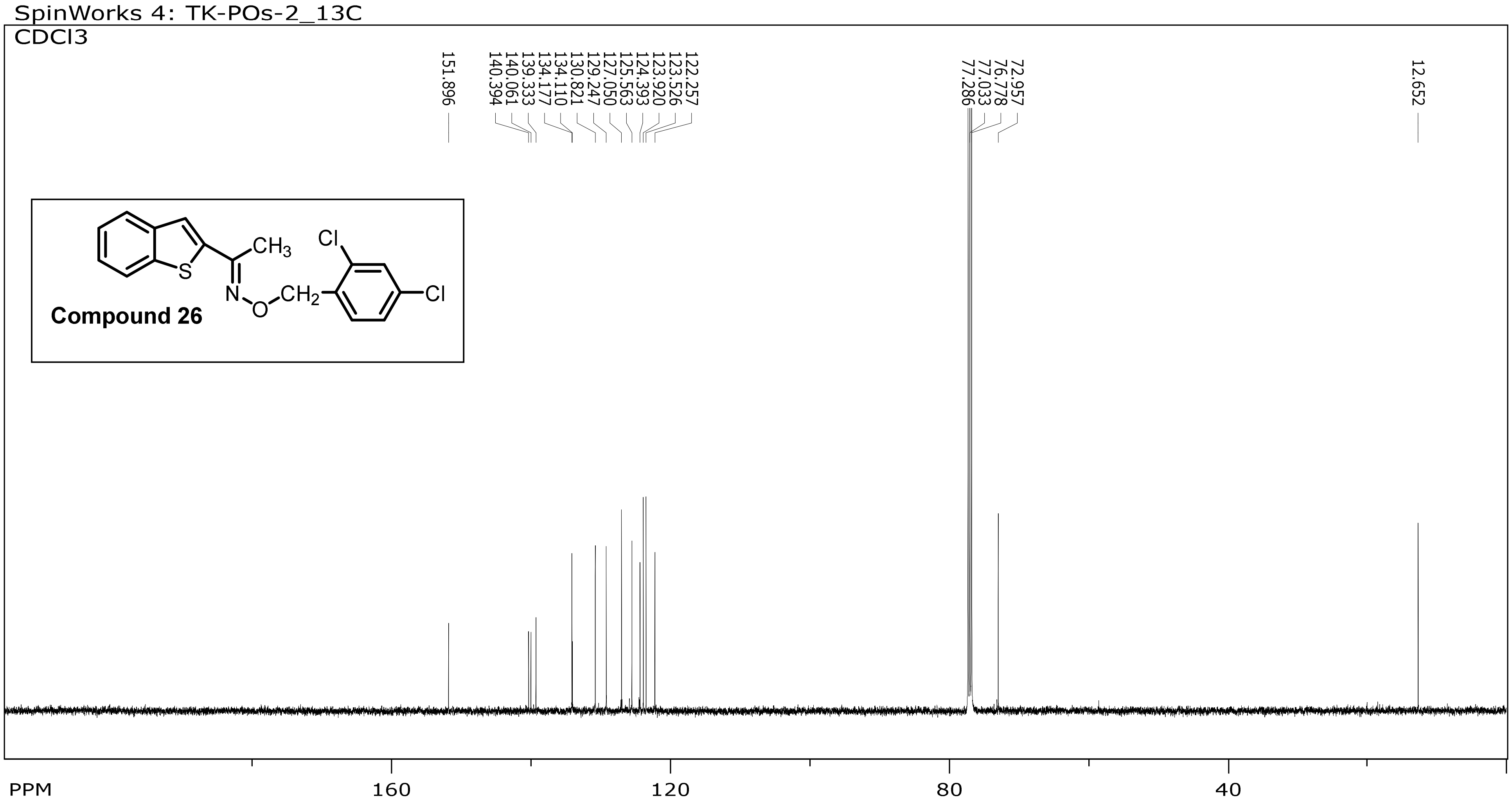

file: ...ers\user\Desktop\Widma 2021\30\fid expt: <zgpg30>
transmitter freq.: 125.803057 MHz
time domain size: 65536 points
width: 31250.00 Hz = 248.4041 ppm = 0.476837 Hz/pt
number of scans: 1024

freq. of 0 ppm: 125.787963 MHz
processed size: 32768 complex points
LB: 1.000 GF: 0.0000

MS spectrum of the compound **26**

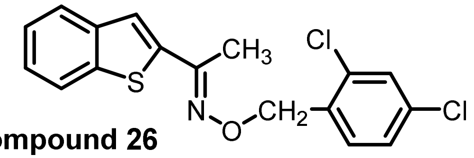

Compound 26

Elemental Composition Report

Single Mass Analysis

Tolerance = 5.0 PPM / DBE: min = -1.5, max = 80.0
Element prediction: Off
Number of isotope peaks used for i-FIT = 9

Monoisotopic Mass, Even Electron Ions
287 formula(e) evaluated with 2 results within limits (all results (up to 1000) for each mass)
Elements Used:
C: 0-30 H: 0-30 N: 0-2 O: 0-5 S: 1-2 Cl: 0-2
210901_Benzotiof_DiCl_A 17 (0.197) Cm (14:27-3:8)

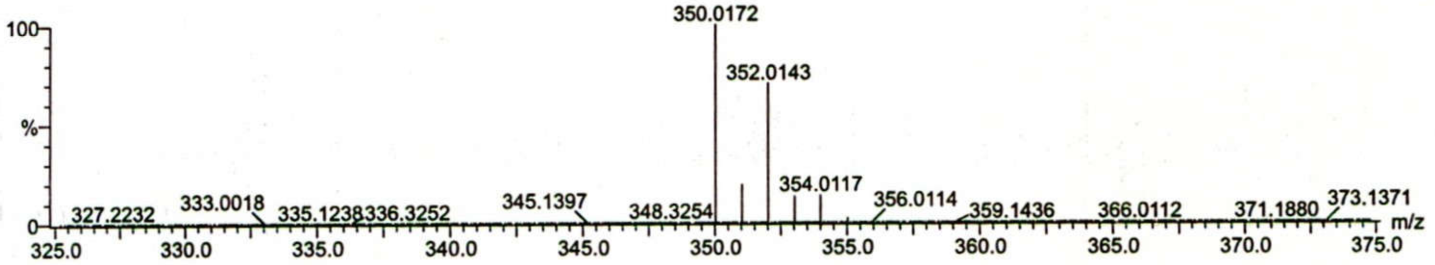

Minimum:				-1.5				
Maximum:		5.0	5.0	80.0				
Mass	Calc. Mass	mDa	PPM	DBE	i-FIT	Norm	Conf(%)	Formula
350.0172	350.0173	-0.1	-0.3	10.5	1616.9	0.000	100.00	C17 H14 N O S Cl2
	350.0157	1.5	4.3	10.5	1632.8	15.837	0.00	C15 H12 N O5 S2

Spectral data of the compound **27.**

^{1}H NMR spectrum of the compound **27**

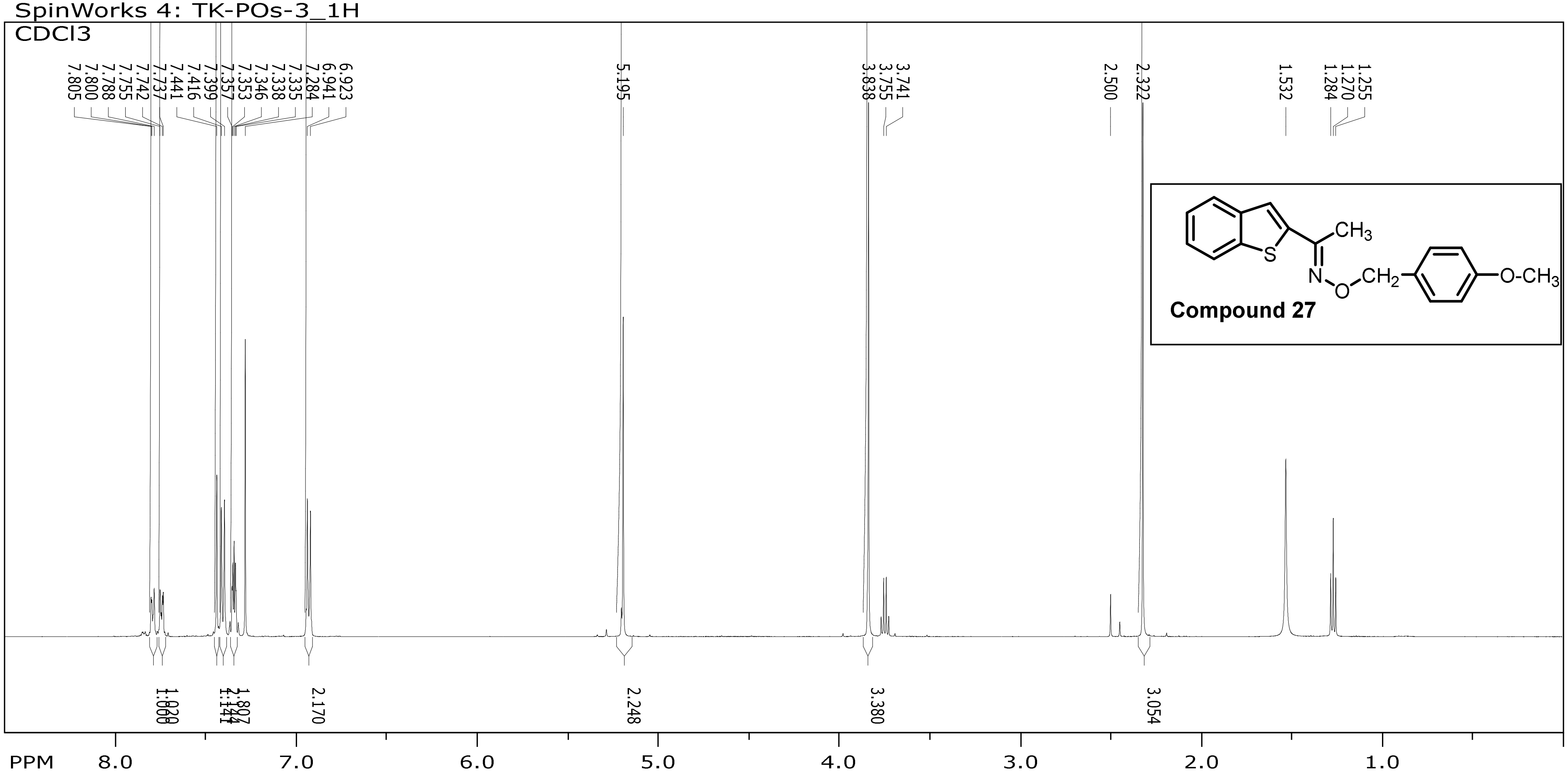

file: ...ers\user\Desktop\Widma 2021\44\fid   expt: <zg30>
transmitter freq.: 500.255003 MHz
time domain size: 65536 points
width: 11029.41 Hz = 22.0476 ppm = 0.168295 Hz/pt
number of scans: 128

freq. of 0 ppm: 500.250000 MHz
processed size: 65536 complex points
LB: 0.300   GF: 0.0000

^{13}C NMR spectrum of the compound **27**

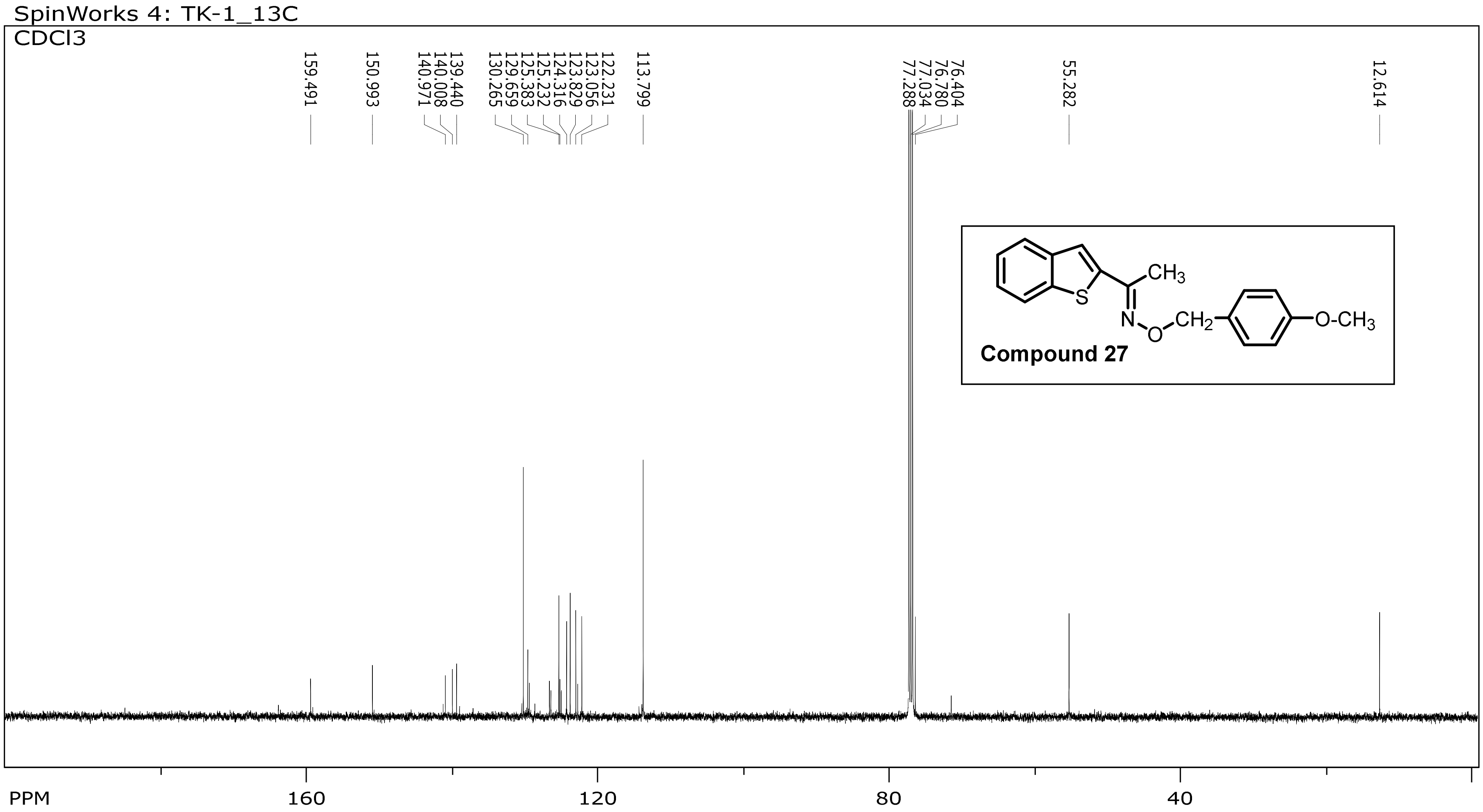

file: ...op\Widma 2021\Widma 09.2021\54\fid expt: <zgpg30>
transmitter freq.: 125.803057 MHz
time domain size: 65536 points
width: 31250.00 Hz = 248.4041 ppm = 0.476837 Hz/pt
number of scans: 1024

freq. of 0 ppm: 125.787963 MHz
processed size: 32768 complex points
LB: 1.000 GF: 0.0000

MS spectrum of the compound **27**

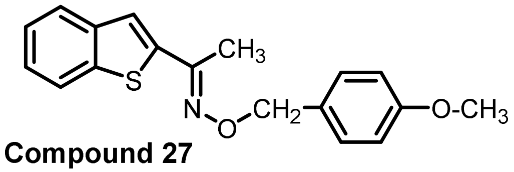

Compound 27

Elemental Composition Report

Single Mass Analysis

Tolerance = 5.0 PPM / DBE: min = -1.5, max = 80.0
Element prediction: Off
Number of isotope peaks used for i-FIT = 9

Monoisotopic Mass, Even Electron Ions
96 formula(e) evaluated with 1 results within limits (all results (up to 1000) for each mass)
Elements Used:
C: 0-30 H: 0-30 N: 0-2 O: 0-5 S: 1-2
210901_BenzotiofOMe_4_B 18 (0.205) Cm (17:24-3:8)

TOF MS ES+
5.56e+005

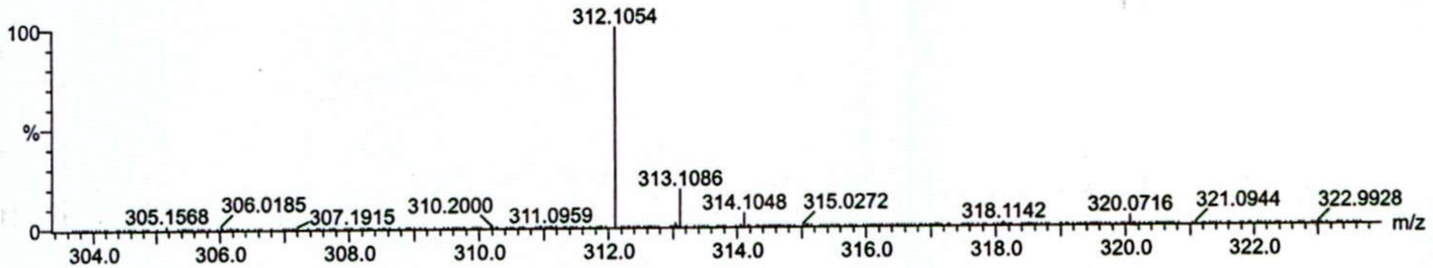

Minimum:				-1.5				
Maximum:		5.0	5.0	80.0				
Mass	Calc. Mass	mDa	PPM	DBE	i-FIT	Norm	Conf(%)	Formula
312.1054	312.1058	-0.4	-1.3	10.5	1426.6	n/a	n/a	C18 H18 N O2 S

S3. NMR Spectra of the other compounds.

^{1}H NMR Spectra of the compounds **1-5**, **7-12** and **16-23**.

^{1}H NMR spectrum of the compound **1**

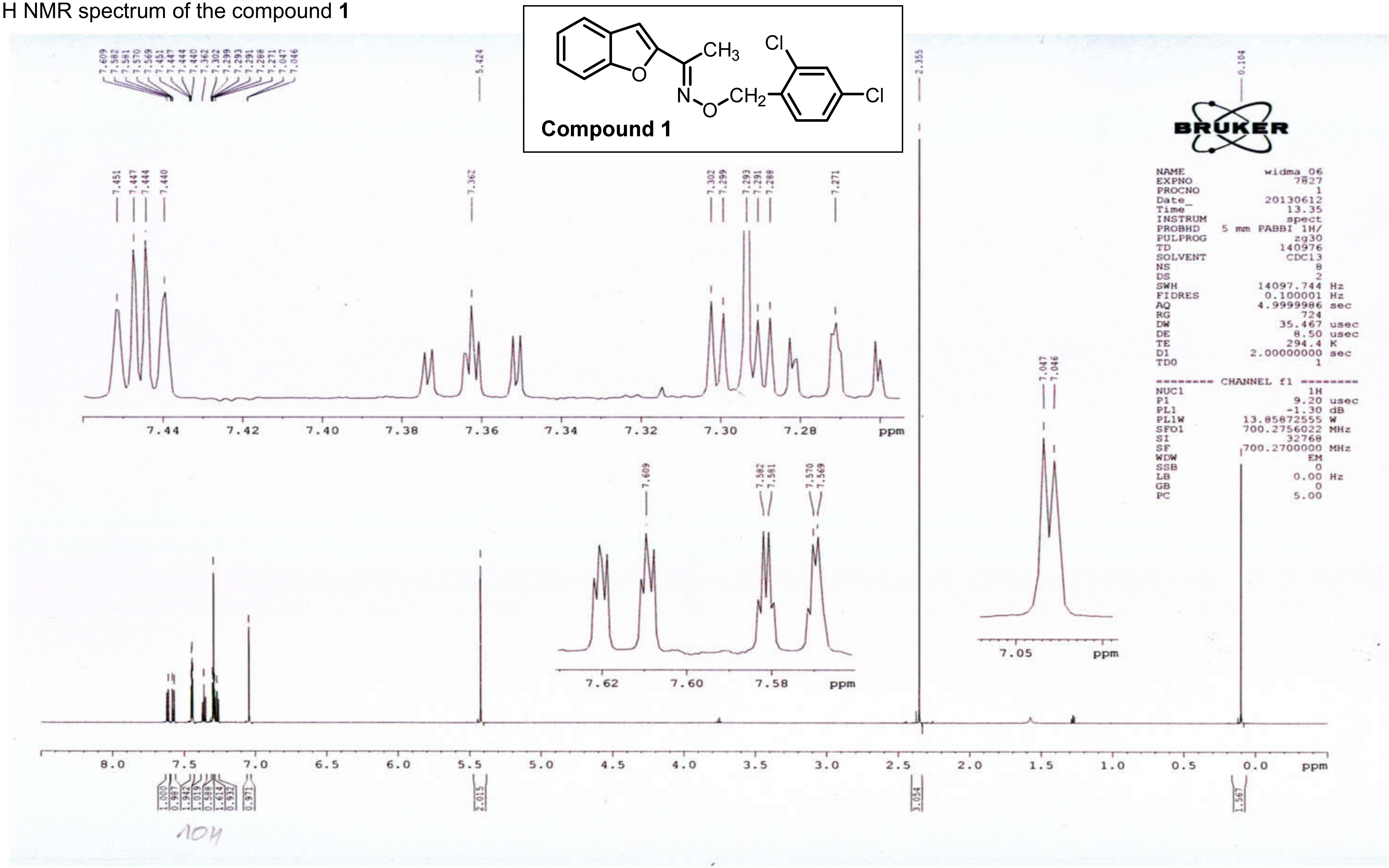

^{1}H NMR spectrum of the compound **2**

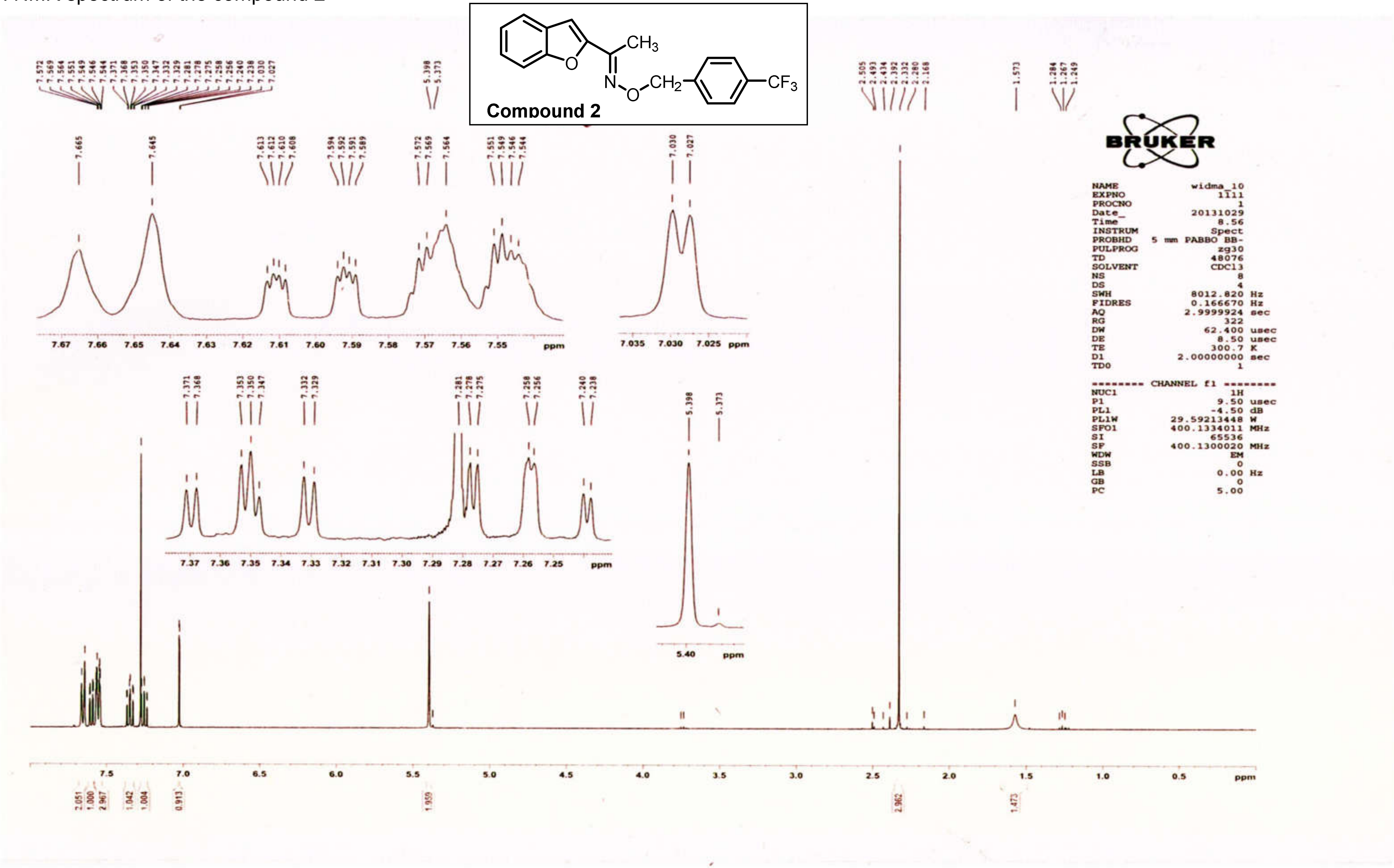

^{1}H NMR spectrum of the compound **3**

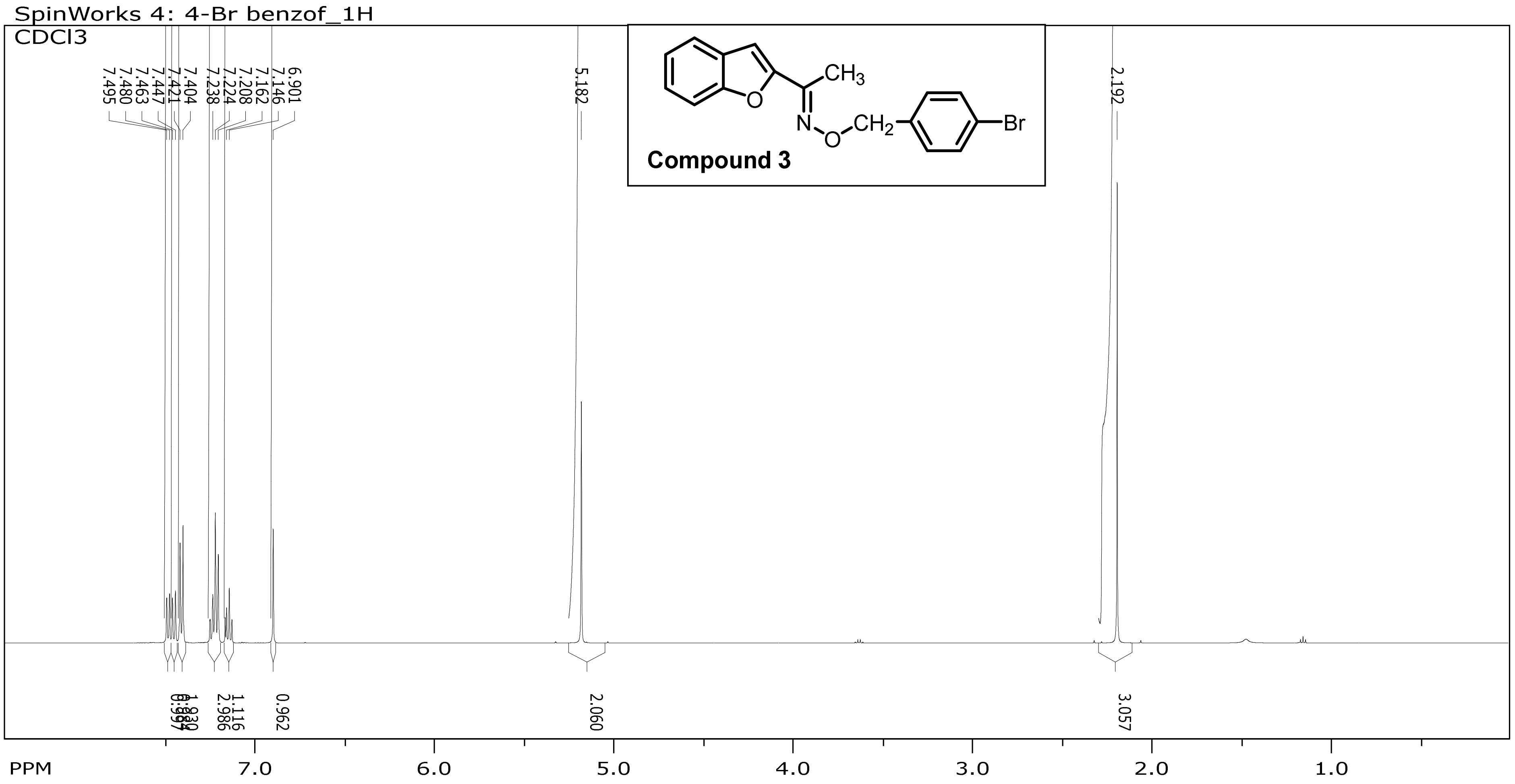

file: ...rs\user\Desktop\Widma 2022\1\1\fid expt: <zg30>
transmitter freq.: 500.255503 MHz
time domain size: 65536 points
width: 11520.74 Hz = 23.0297 ppm = 0.175793 Hz/pt
number of scans: 128

freq. of 0 ppm: 500.250057 MHz
processed size: 65536 complex points
LB: 0.300 GF: 0.0000

^{1}H NMR spectrum of the compound **4**

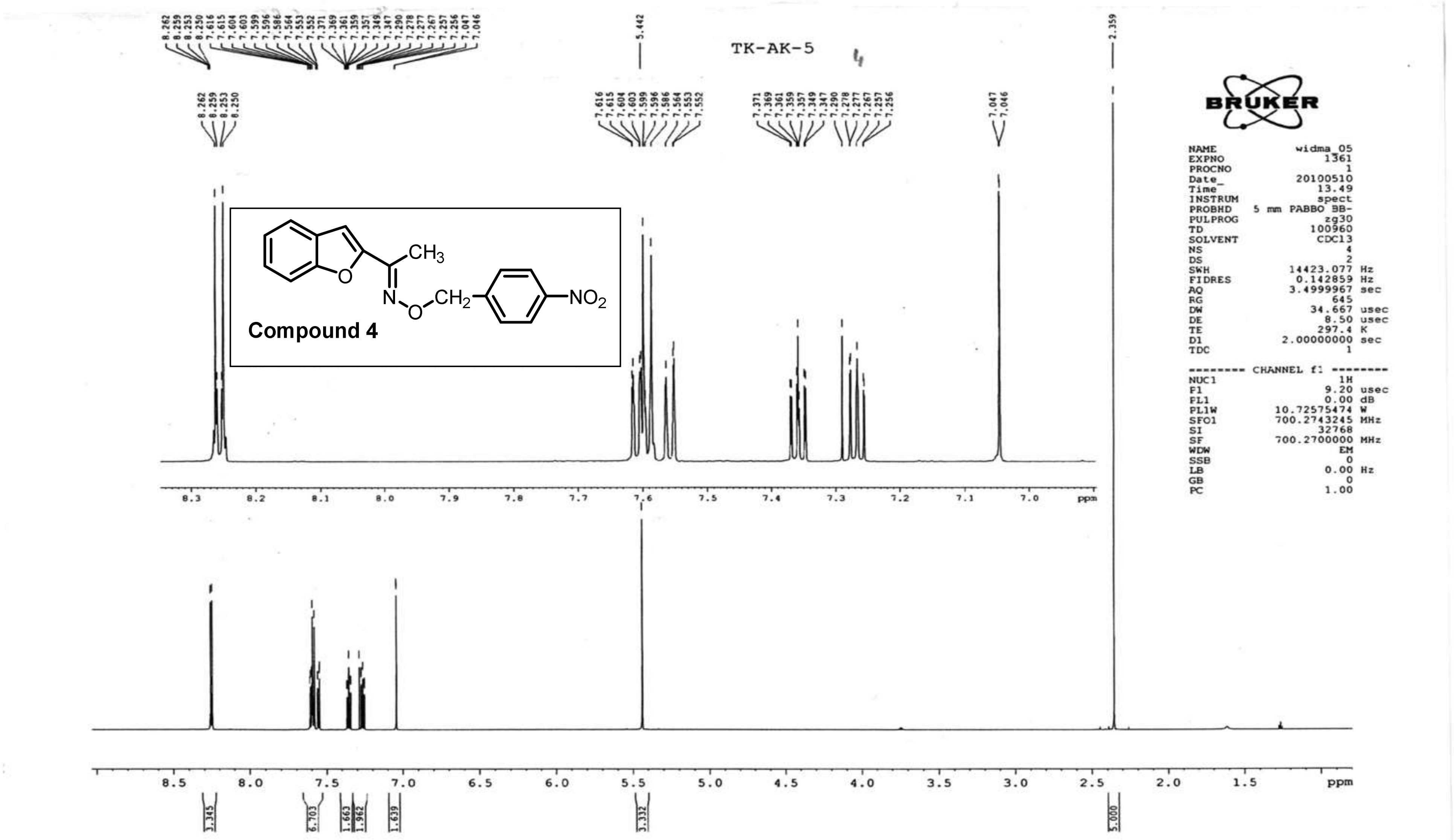

^{1}H NMR spectrum of the compound **5**

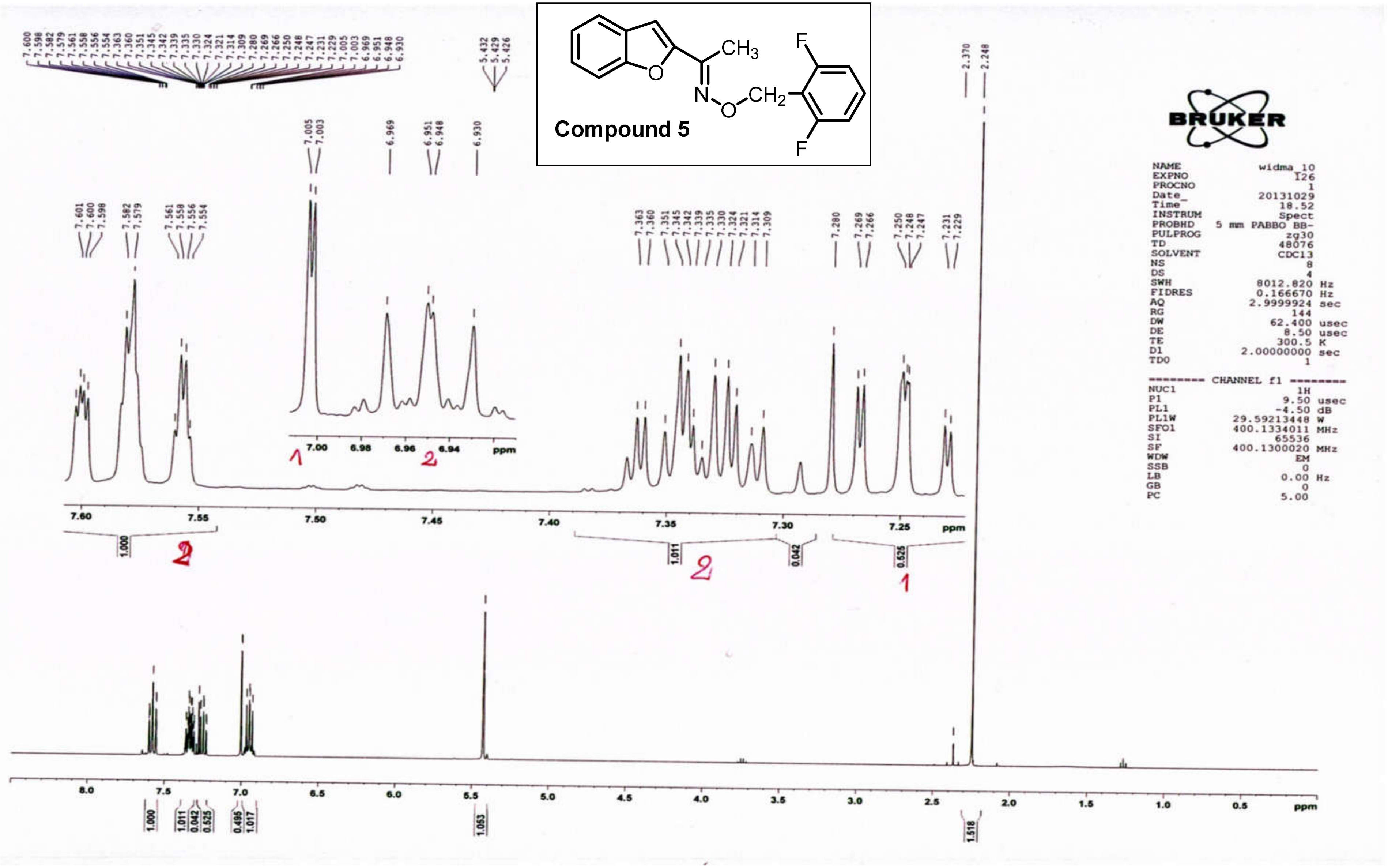

^{1}H NMR spectrum of the compound **7**

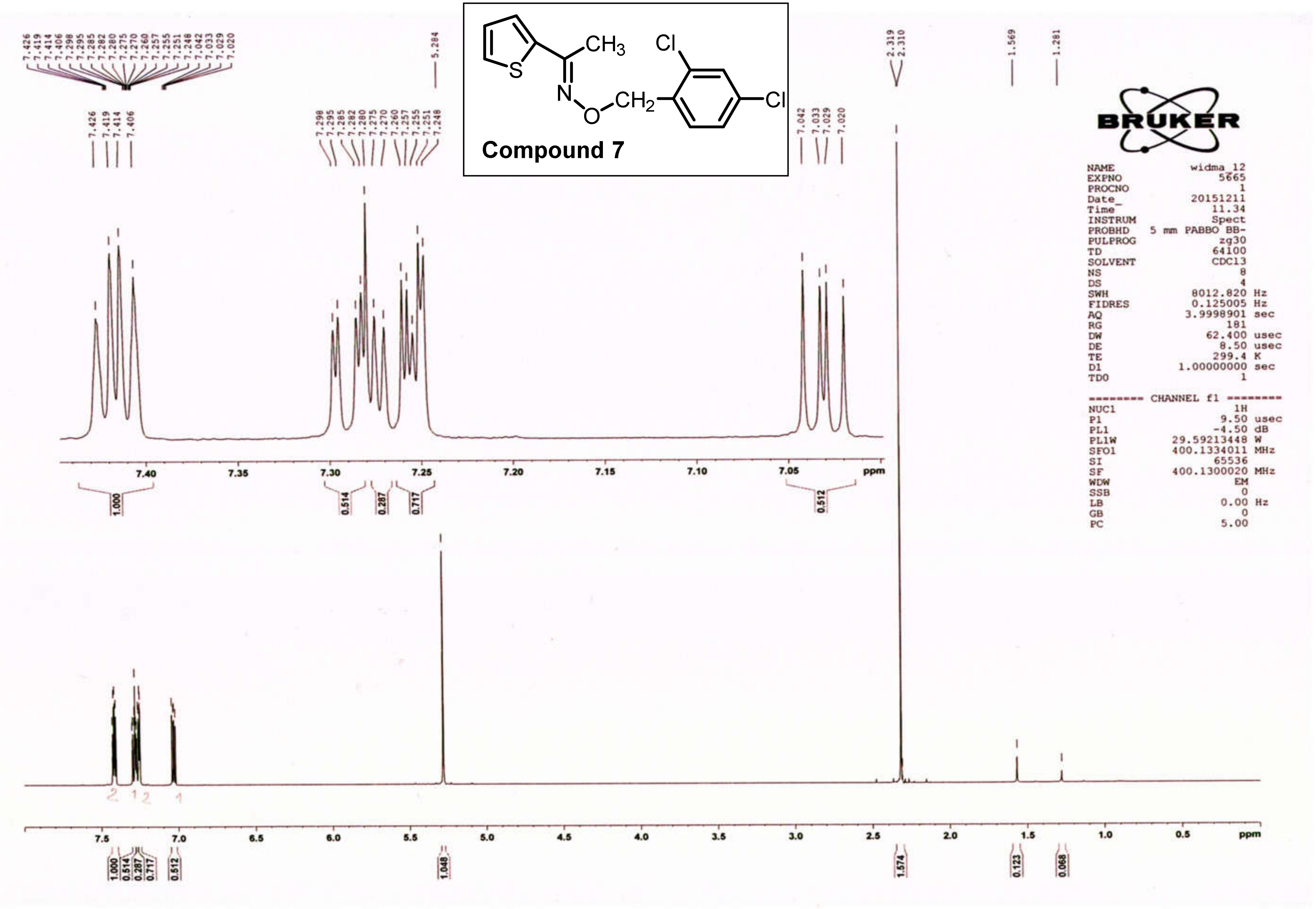

^{1}H NMR spectrum of the compound **8**

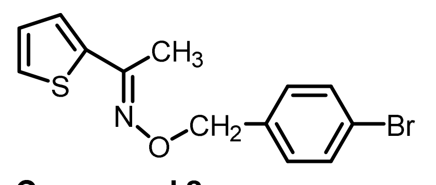

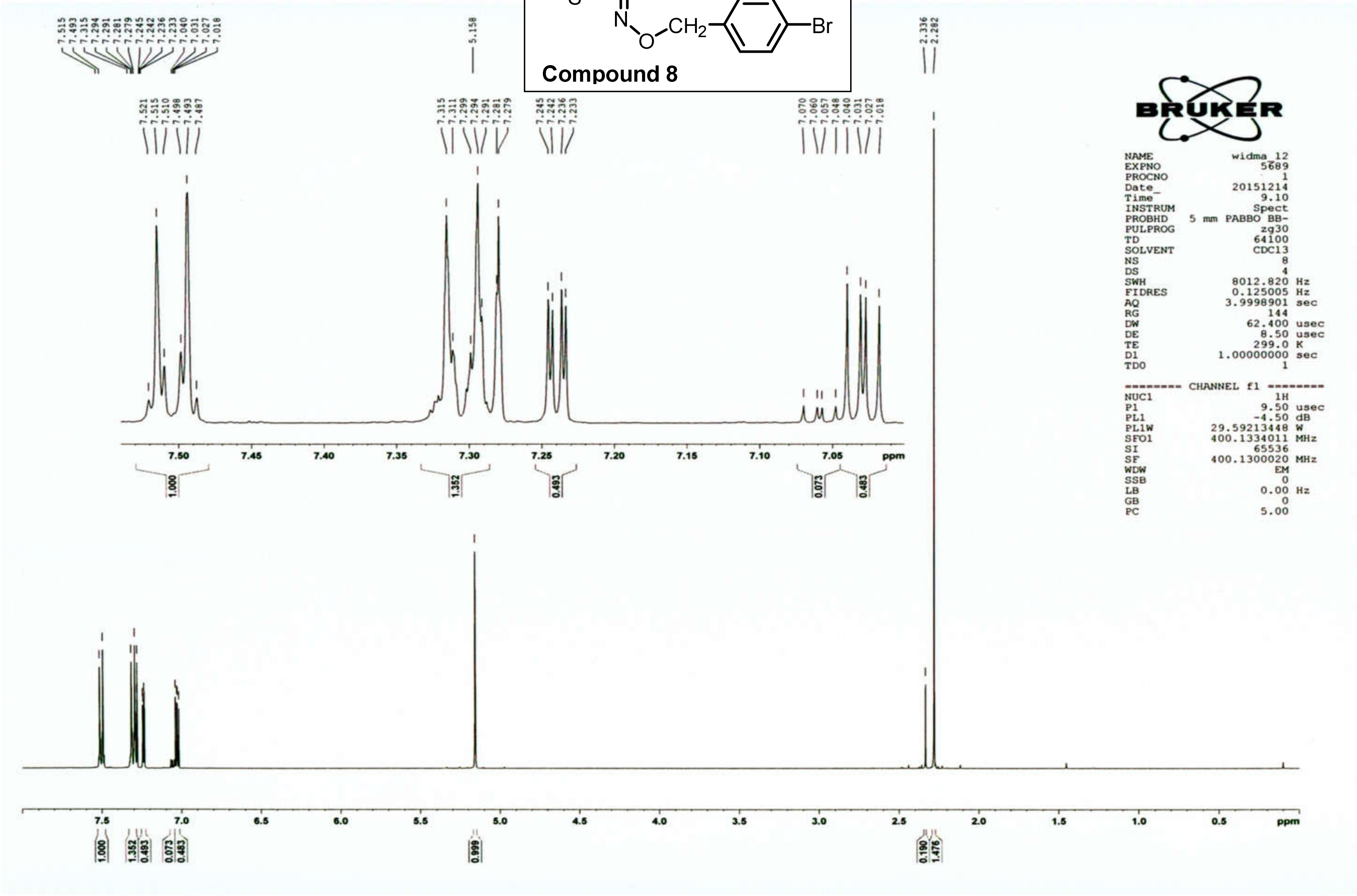

^{1}H NMR spectrum of the compound **9**

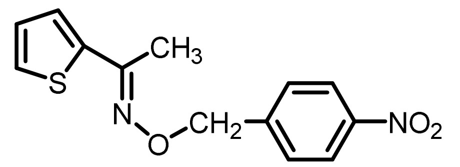

Compound 9

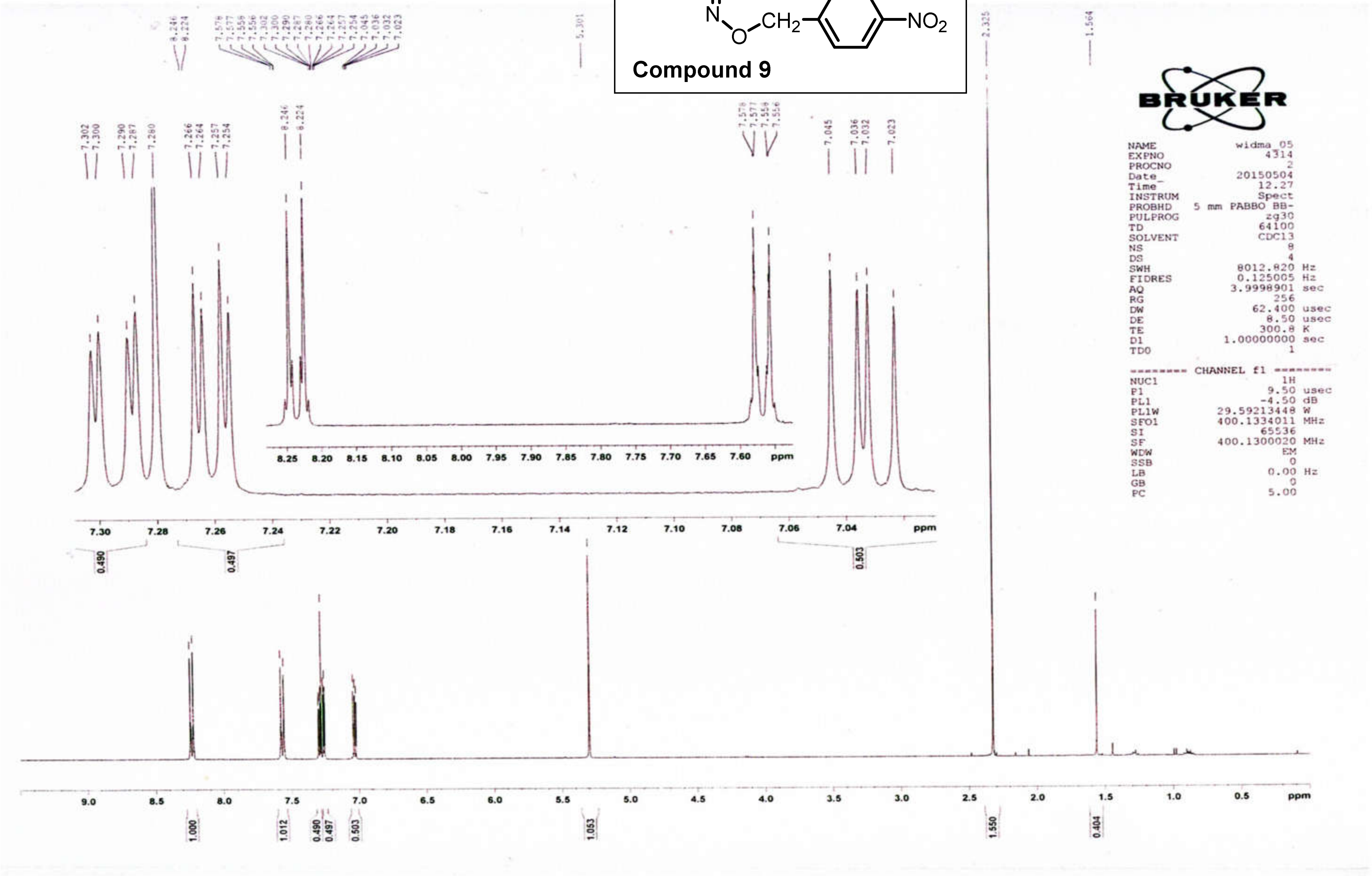

^{1}H NMR spectrum of the compound **10**

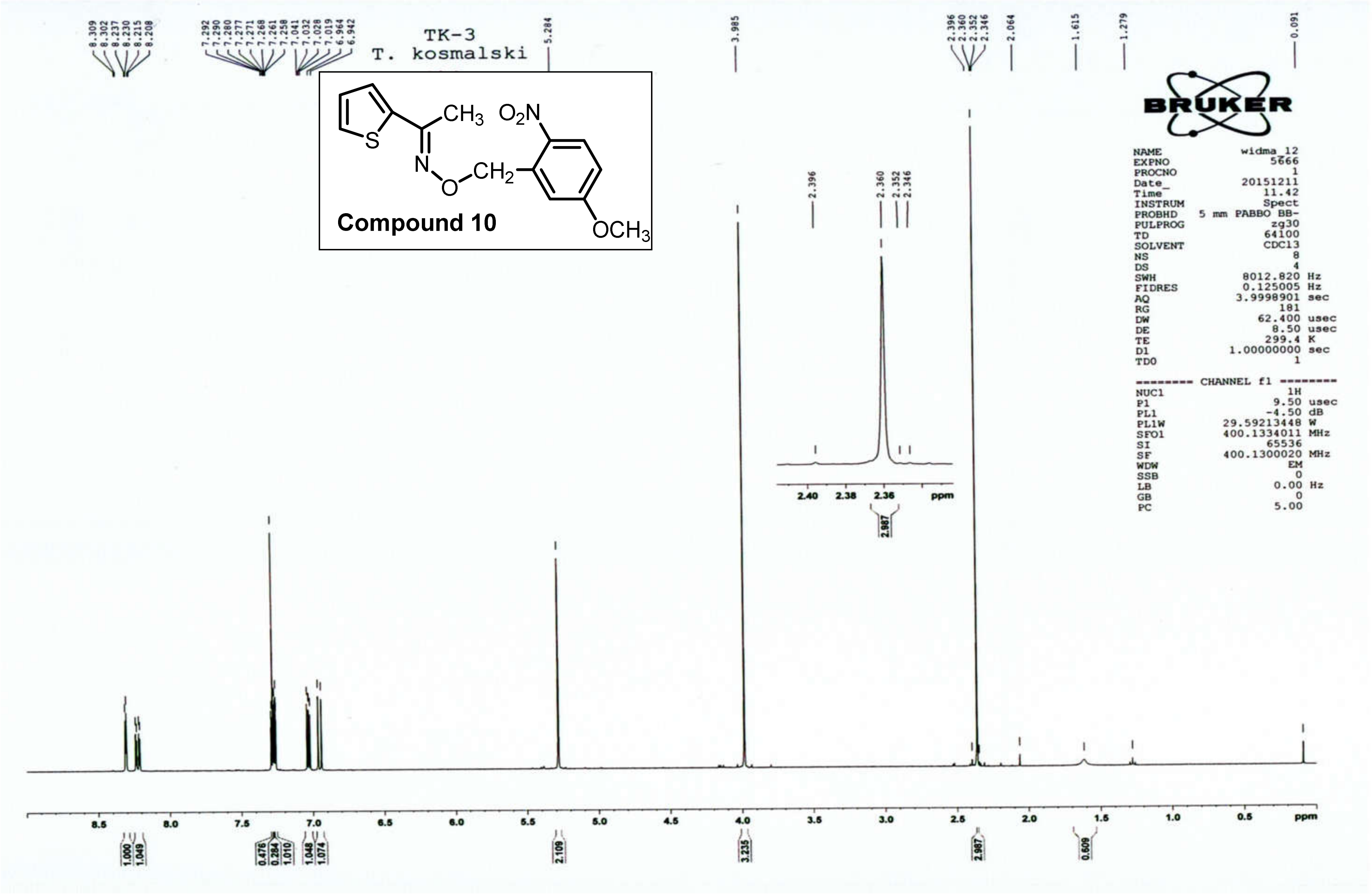

^{1}H NMR spectrum of the compound **11**

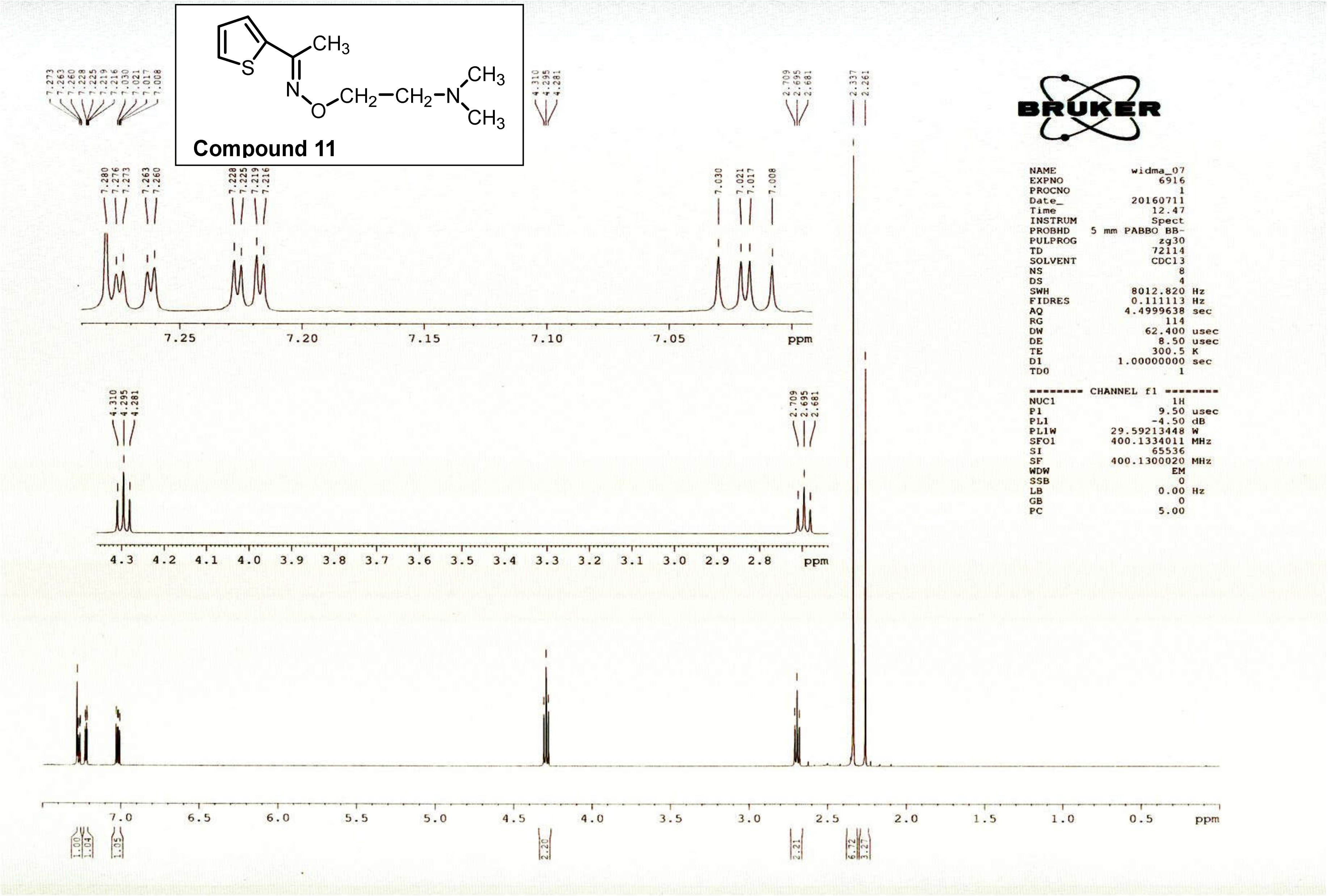

^{1}H NMR spectrum of the compound **12**

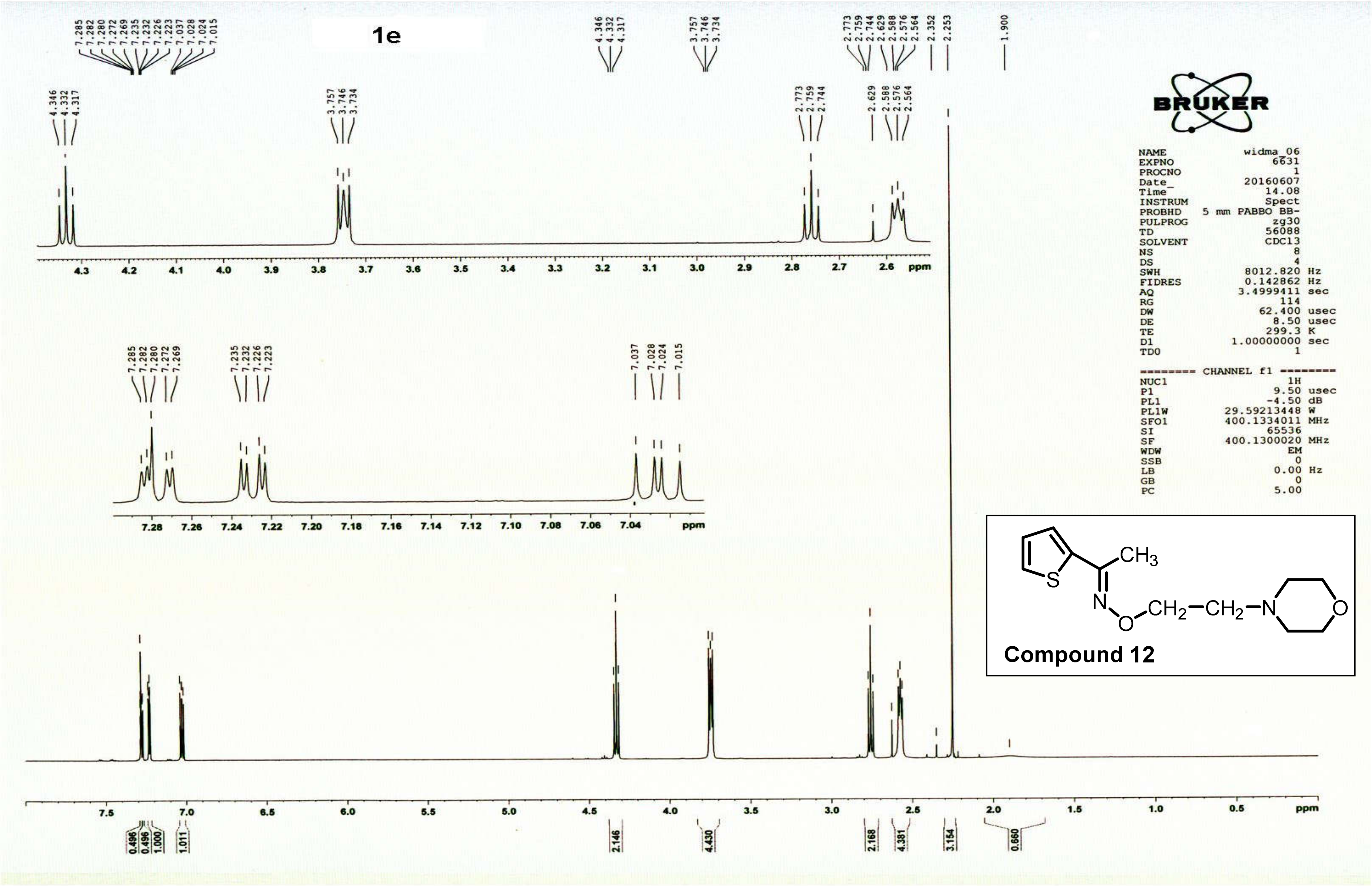

Compound 12

^{1}H NMR spectrum of the compound **16**

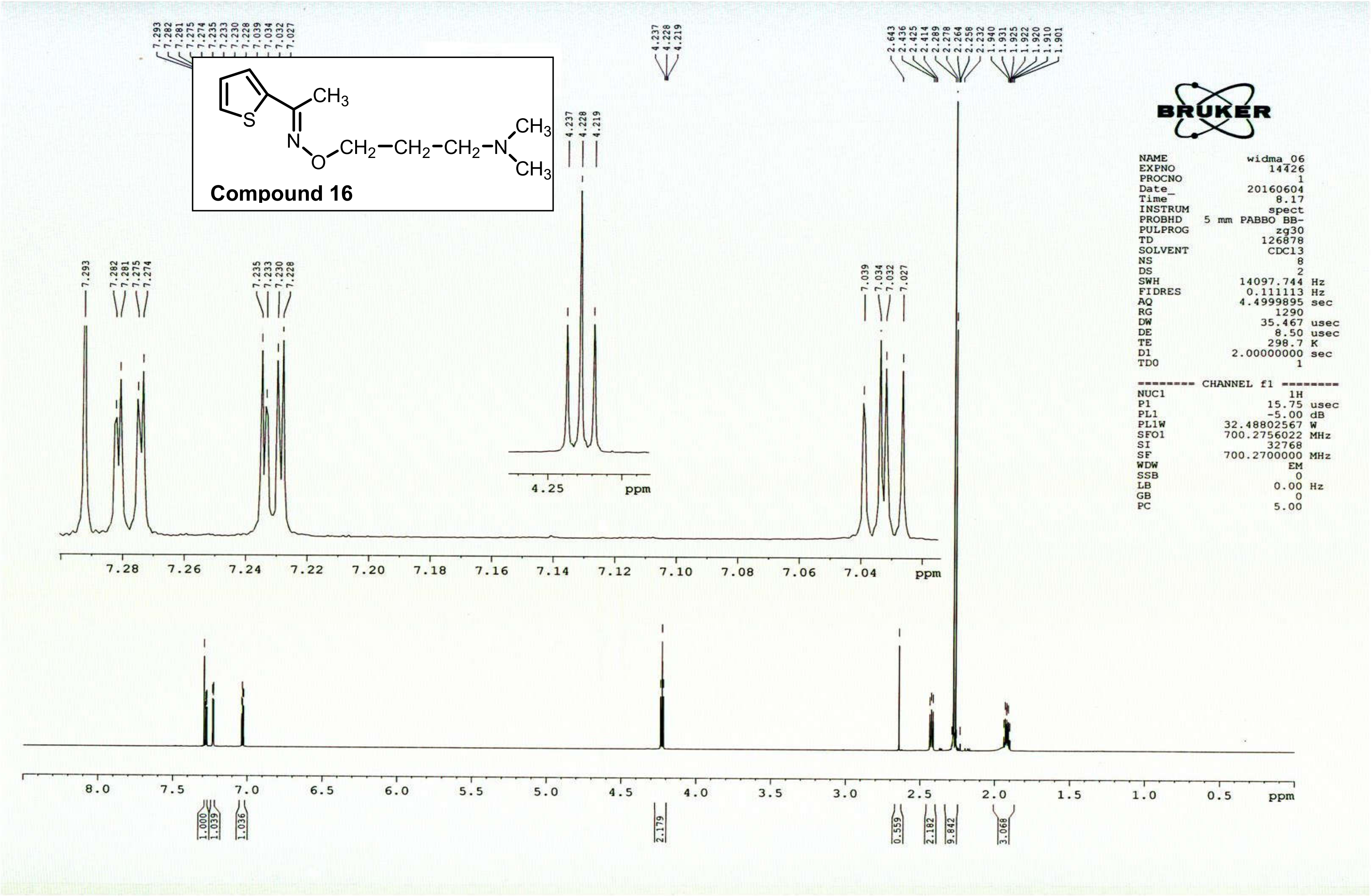

^{1}H NMR spectrum of the compound **17**

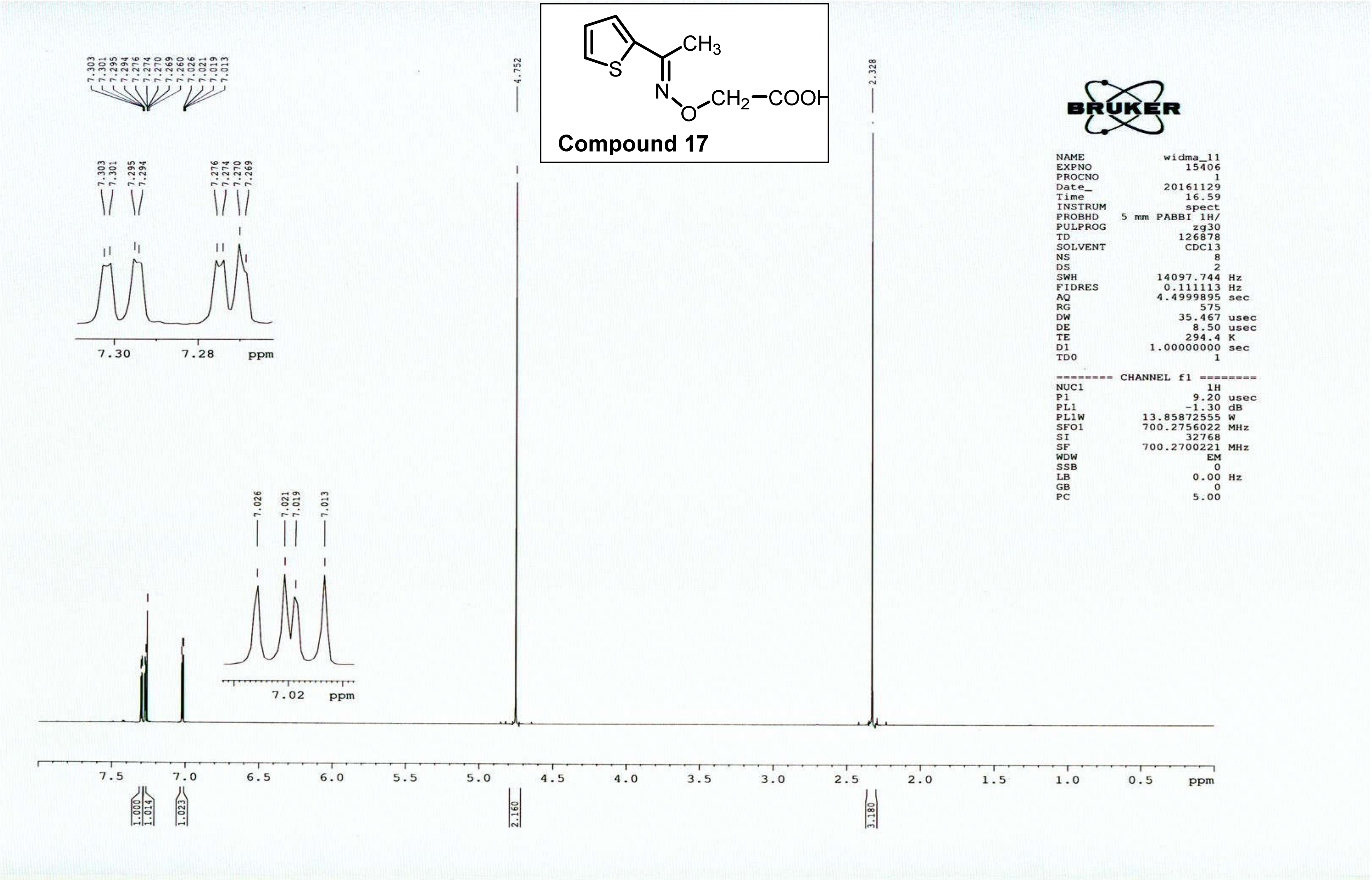

^{1}H NMR spectrum of the compound **18**

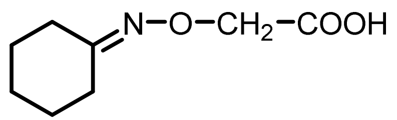

Compound 18

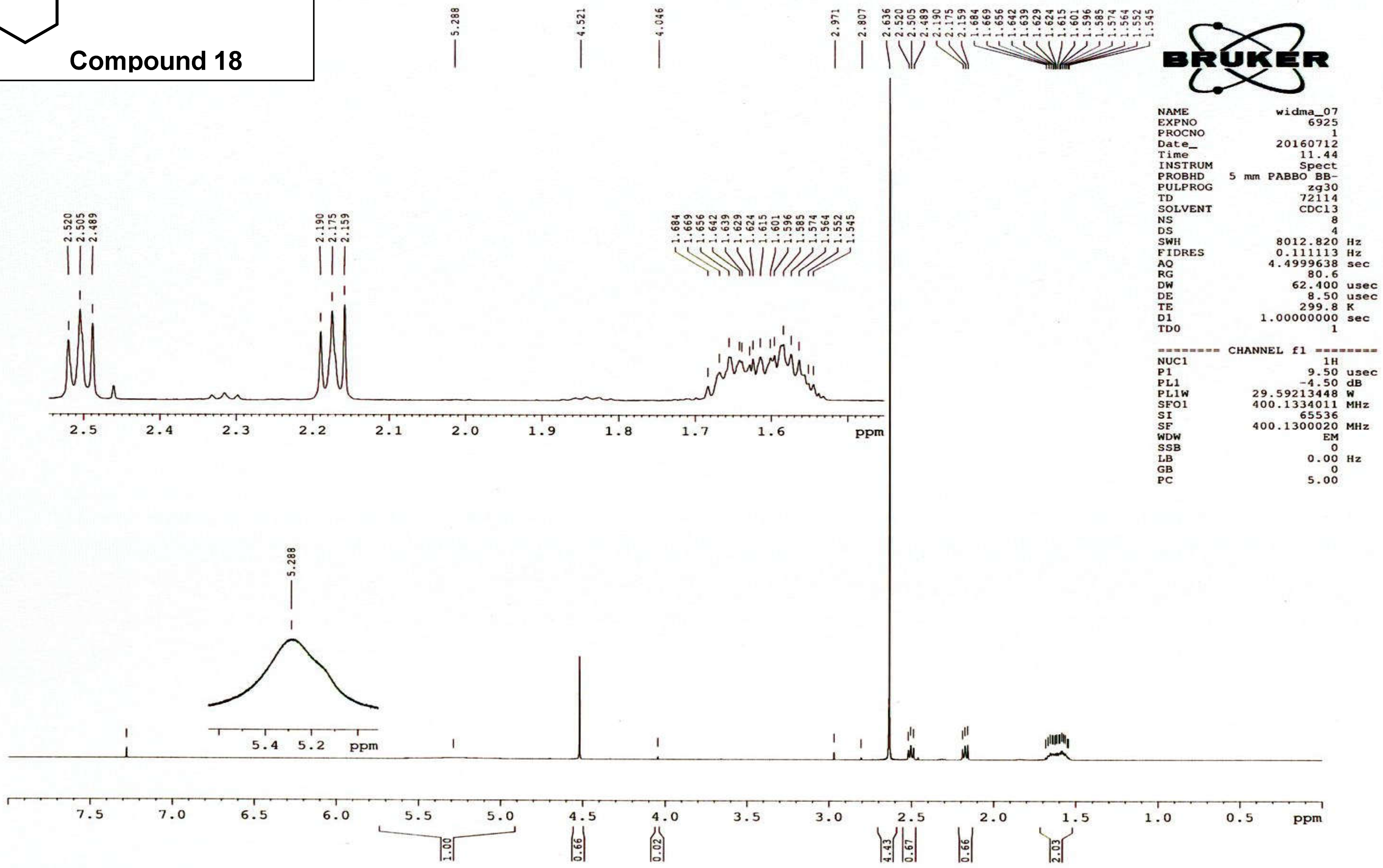

^{1}H NMR spectrum of the compound **19**

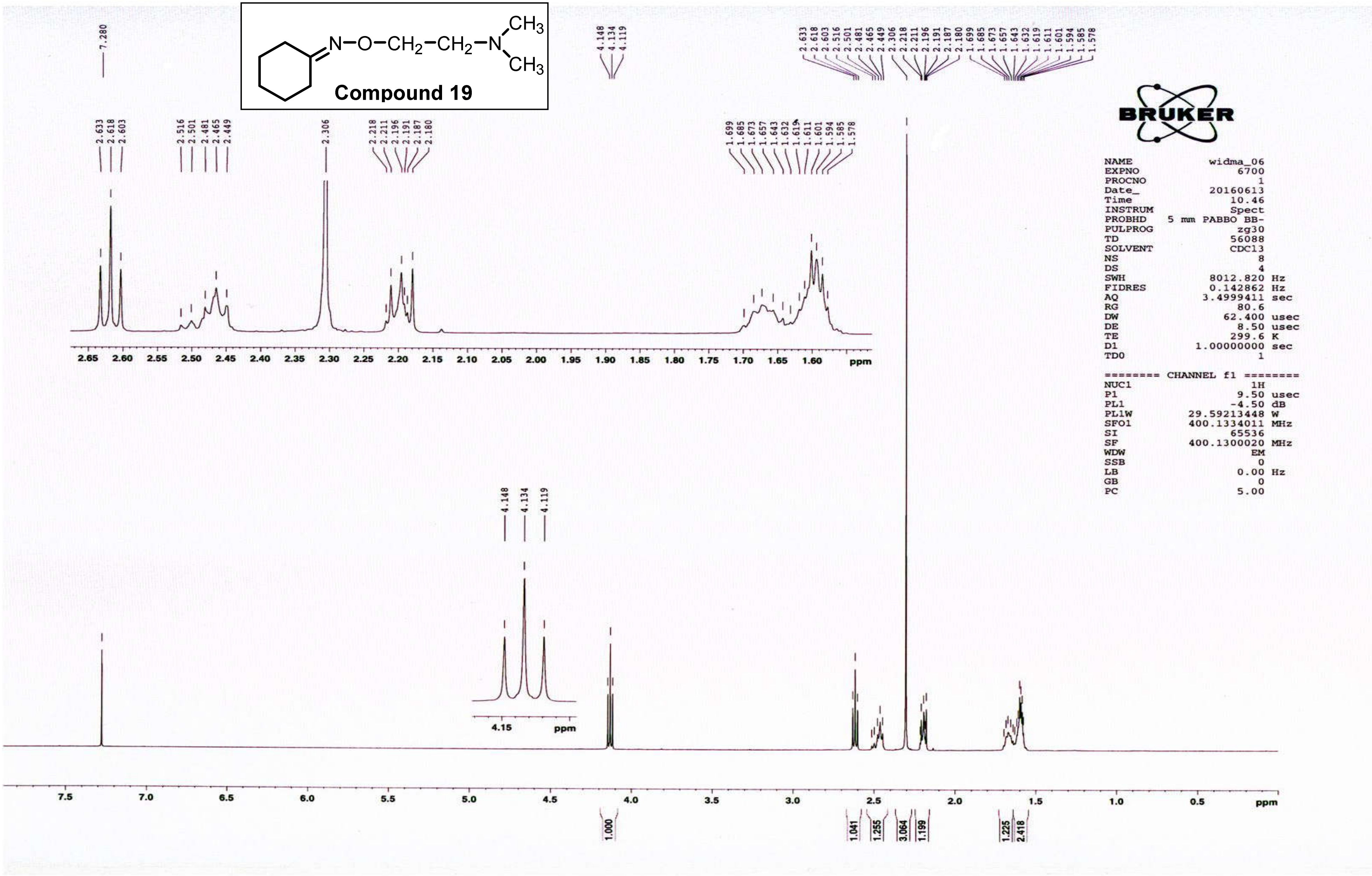

^{1}H NMR spectrum of the compound **20**

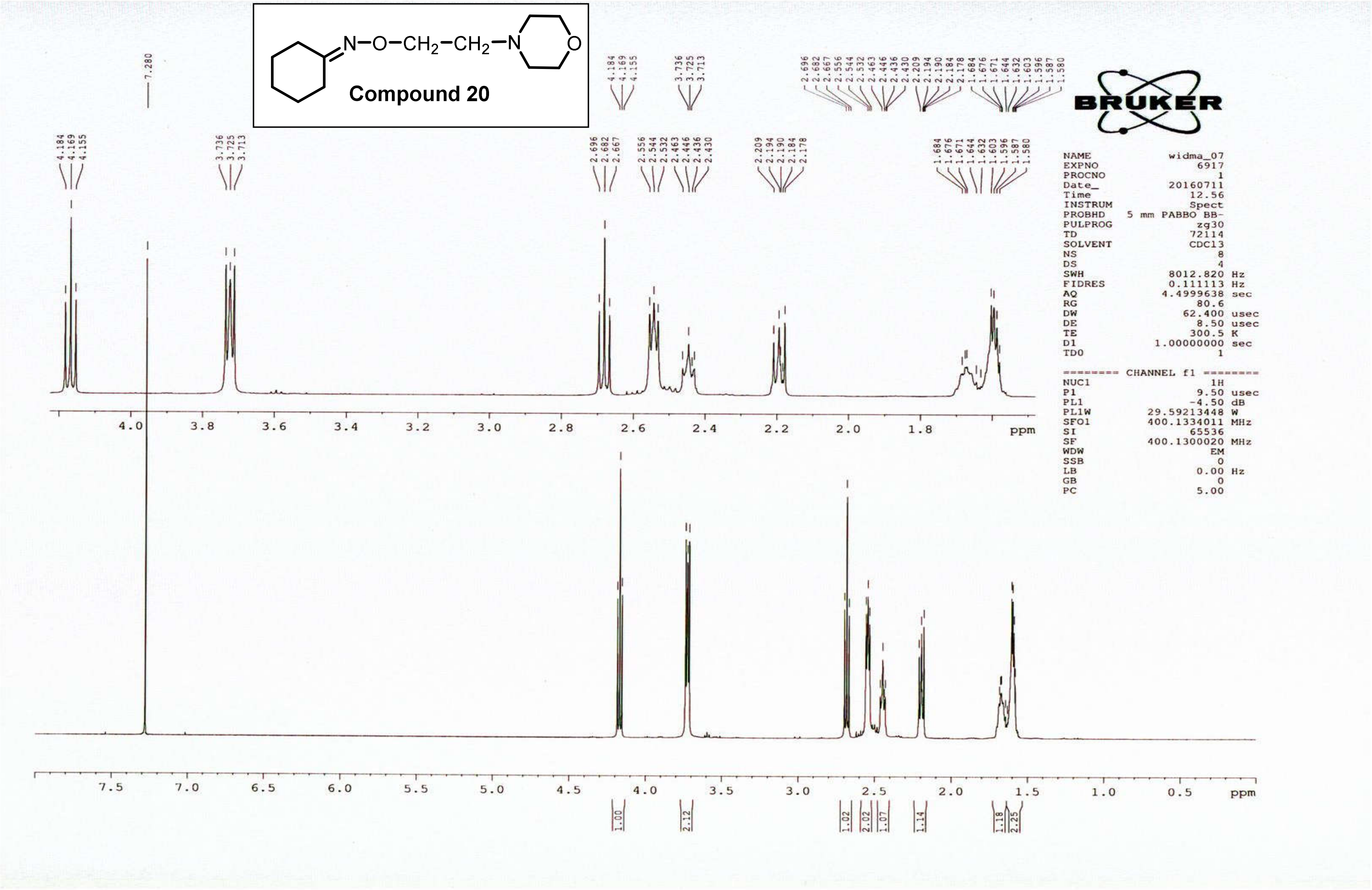

^{1}H NMR spectrum of the compound **21**

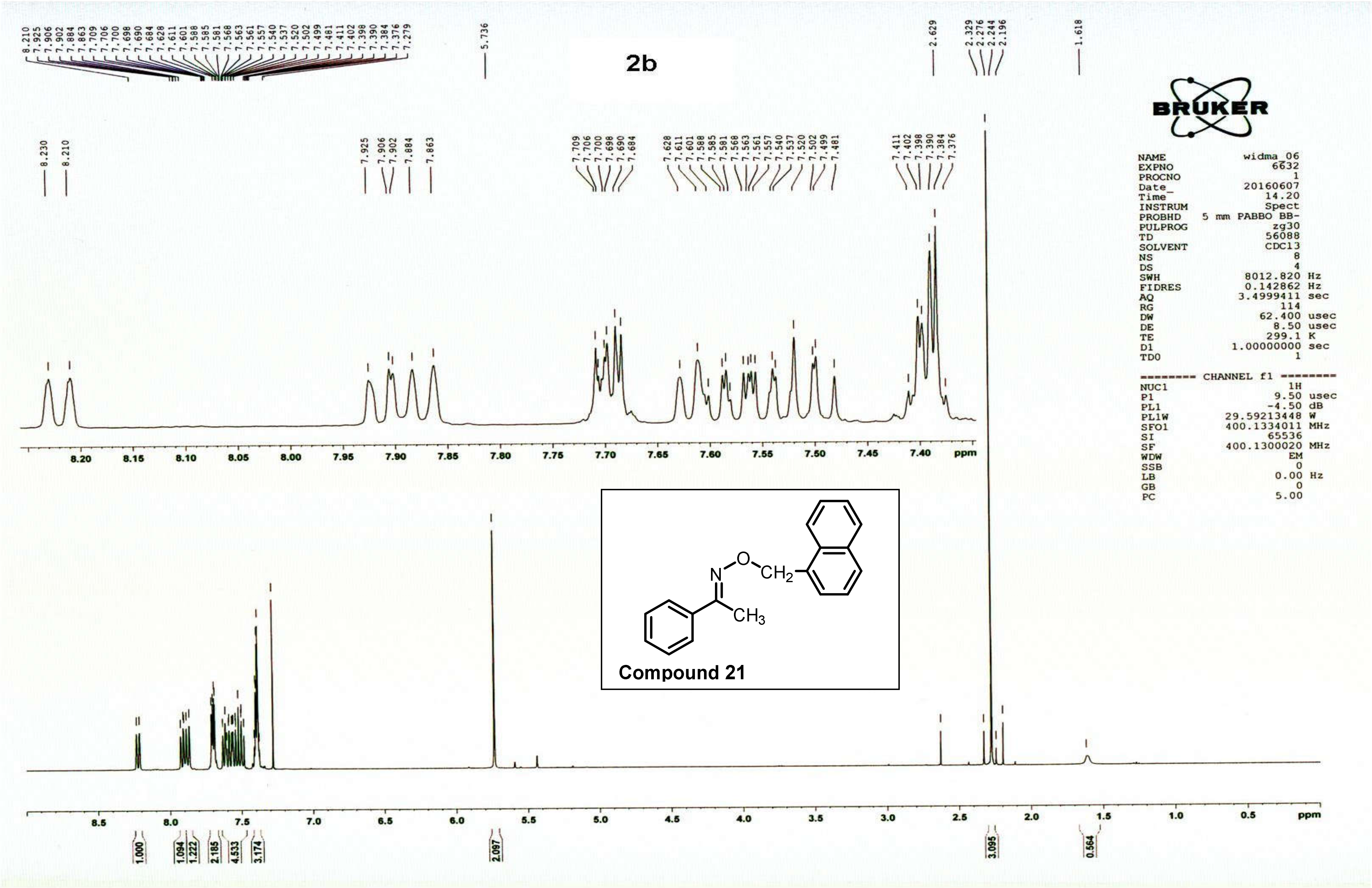

^{1}H NMR spectrum of the compound **22**

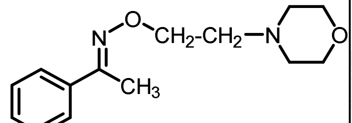

Compound 22

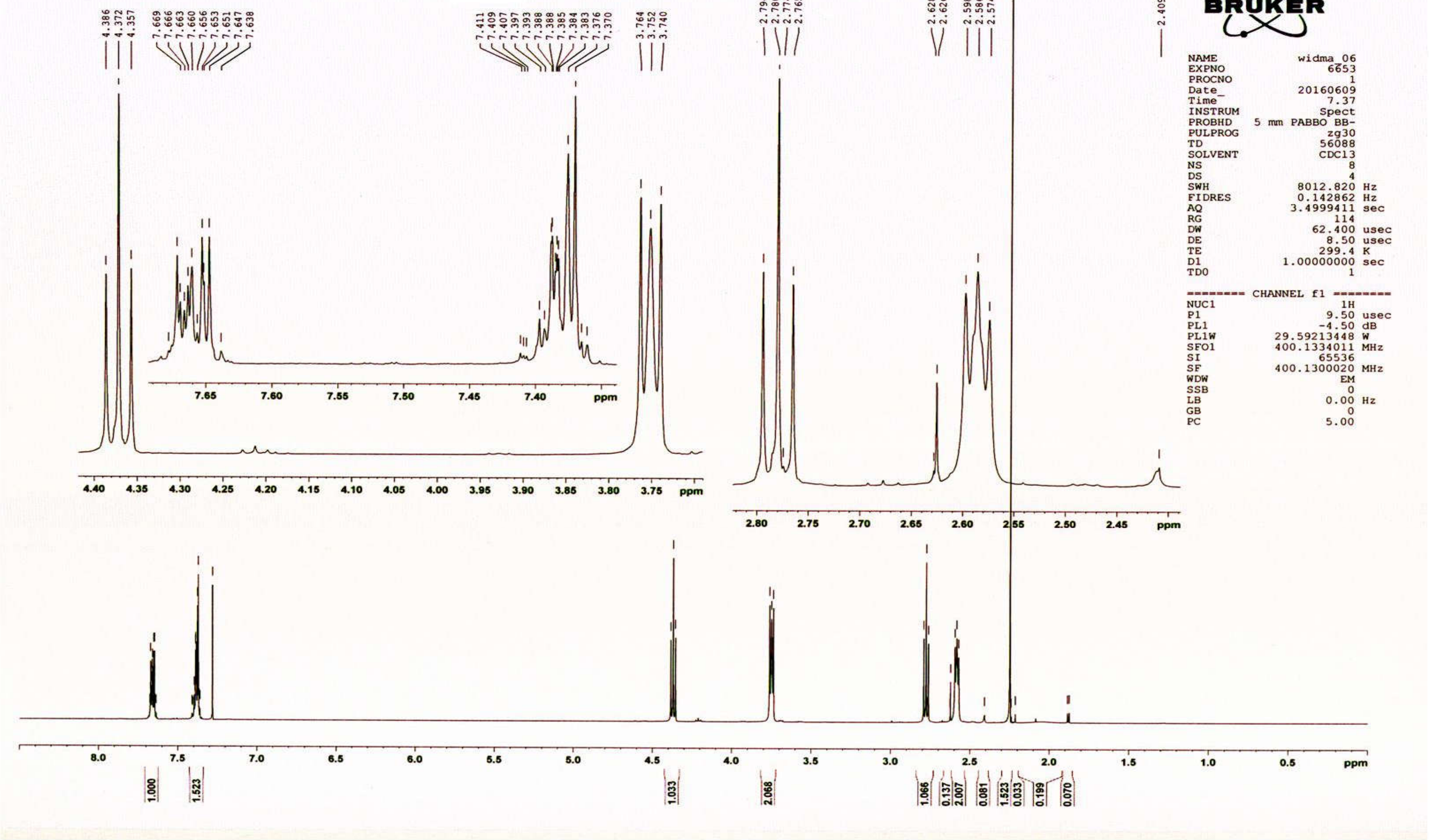

^{1}H NMR spectrum of the compound **23**

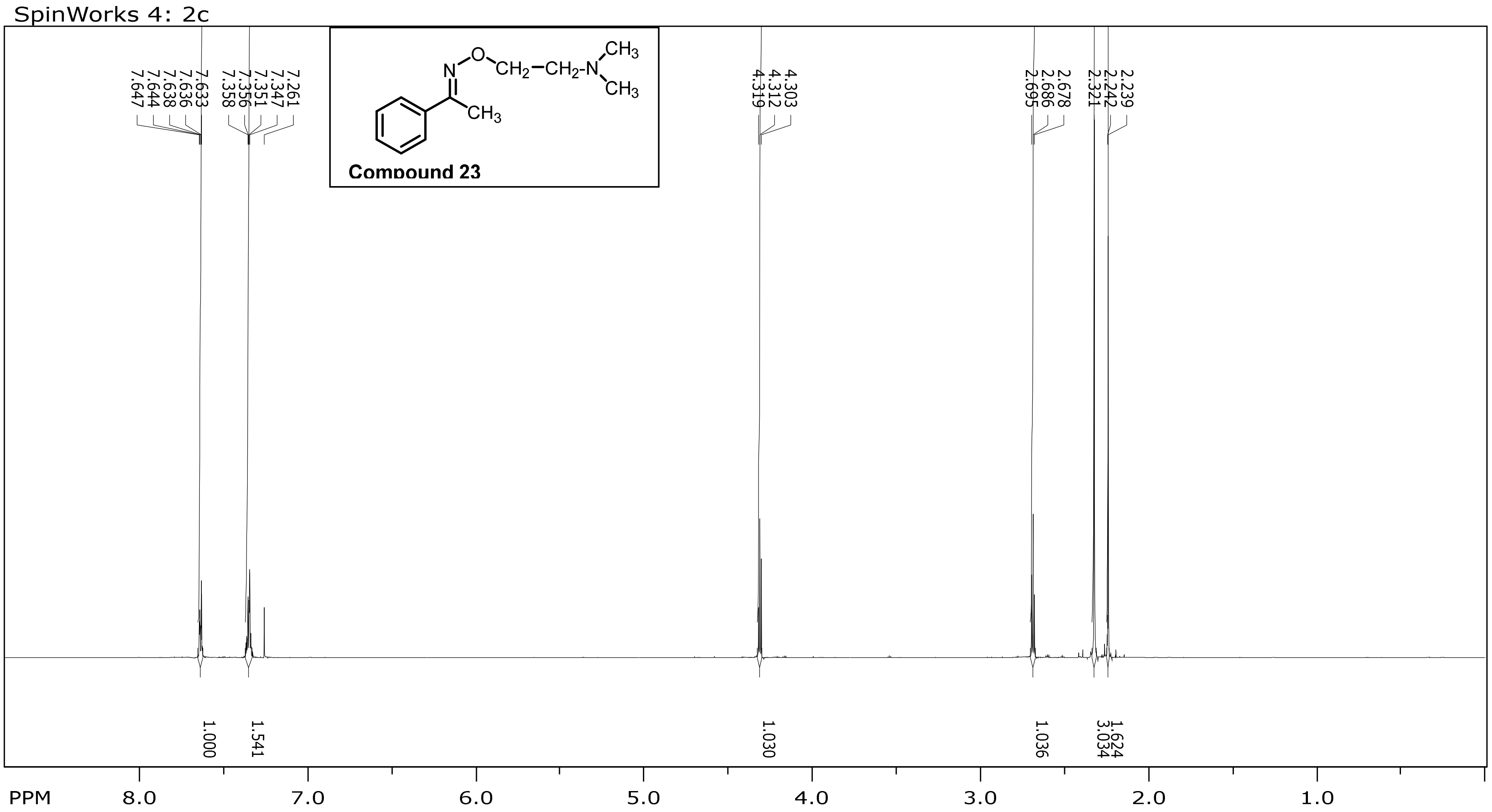

file: ...rs\Tomek\Desktop\NMR 700\15405\fid expt: <zg30>
transmitter freq.: 700.275602 MHz
time domain size: 126878 points
width: 14097.74 Hz = 20.1317 ppm = 0.111113 Hz/pt
number of scans: 8

freq. of 0 ppm: 700.270022 MHz
processed size: 16384 complex points
LB: 0.000 GF: 0.0000

^{13}C NMR Spectra of the compounds **1-5**, **7-12** and **16-23**.

^{13}C NMR spectrum of the compound **1**

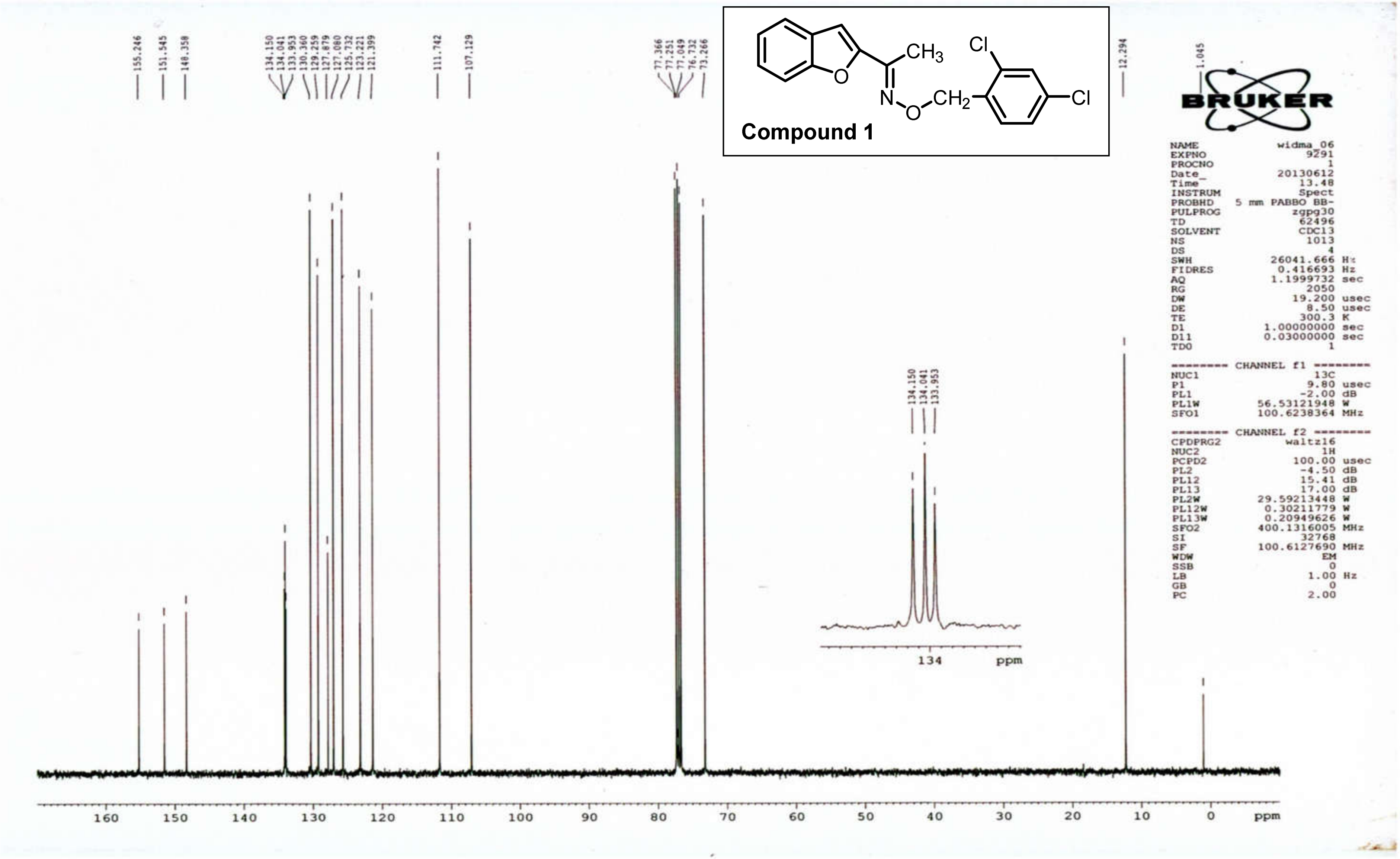

^{13}C NMR spectrum of the compound **2**

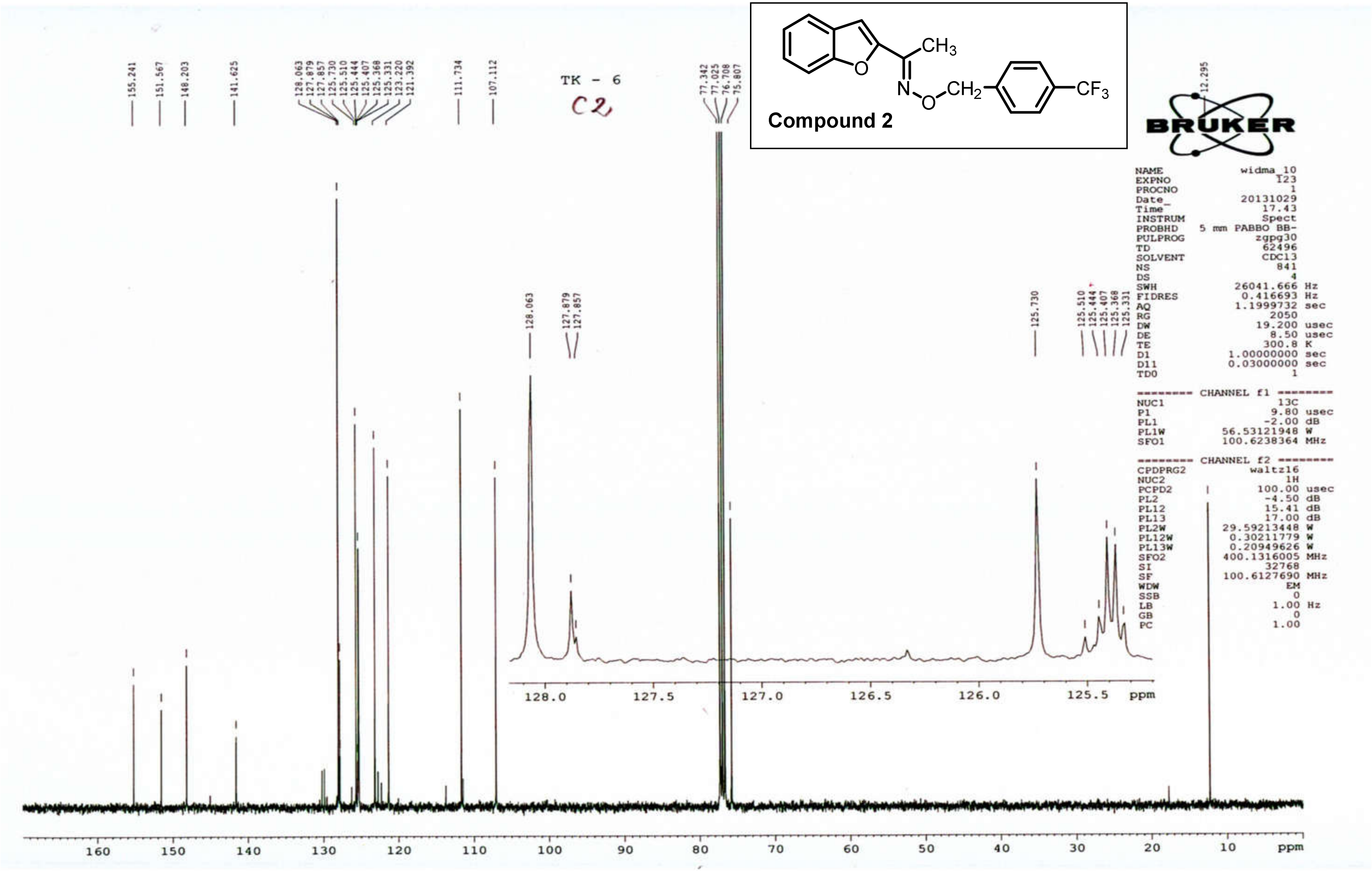

^{13}C NMR spectrum of the compound **3**

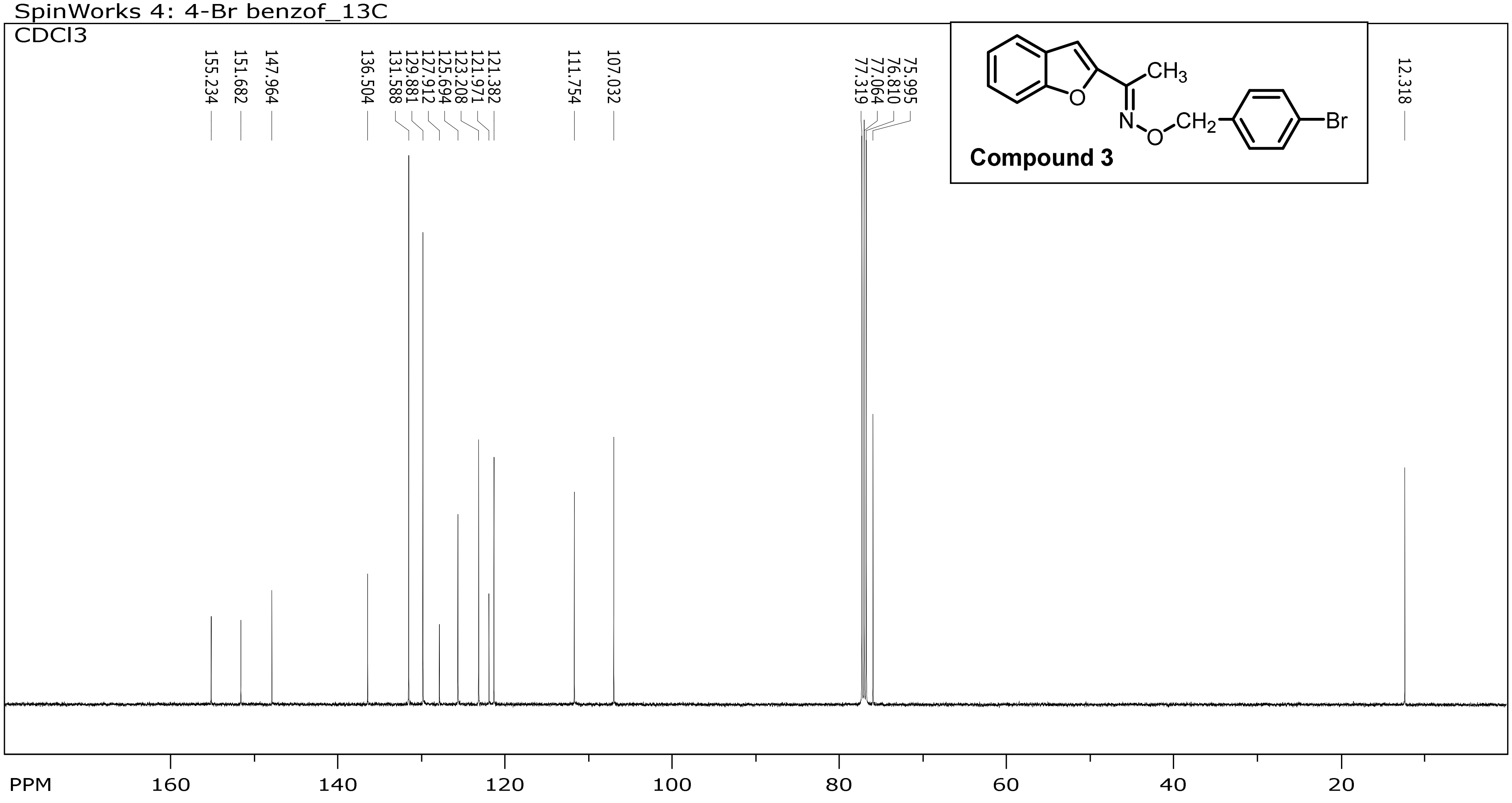

file: ...rs\user\Desktop\Widma 2022\2\2\fid expt: <zgpg30>
transmitter freq.: 125.803057 MHz
time domain size: 65536 points
width: 32894.74 Hz = 261.4780 ppm = 0.501934 Hz/pt
number of scans: 1024

freq. of 0 ppm: 125.787963 MHz
processed size: 32768 complex points
LB: 1.000 GF: 0.0000

^{13}C NMR spectrum of the compound **4**

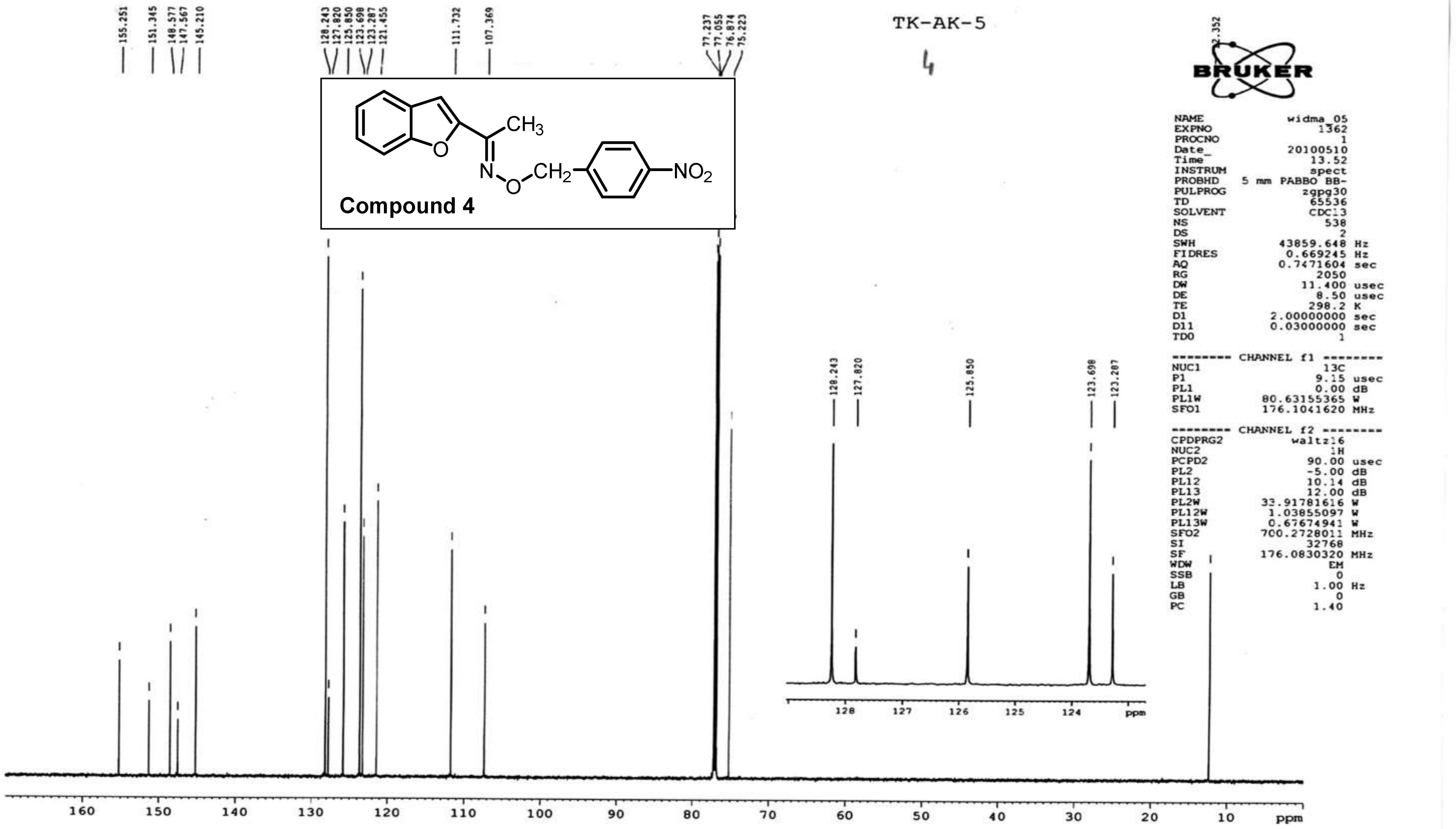

^{13}C NMR spectrum of the compound **5**

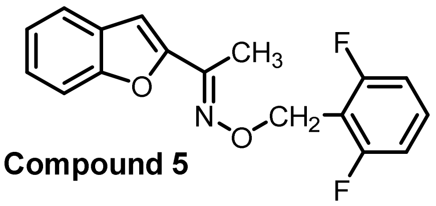

Compound 5

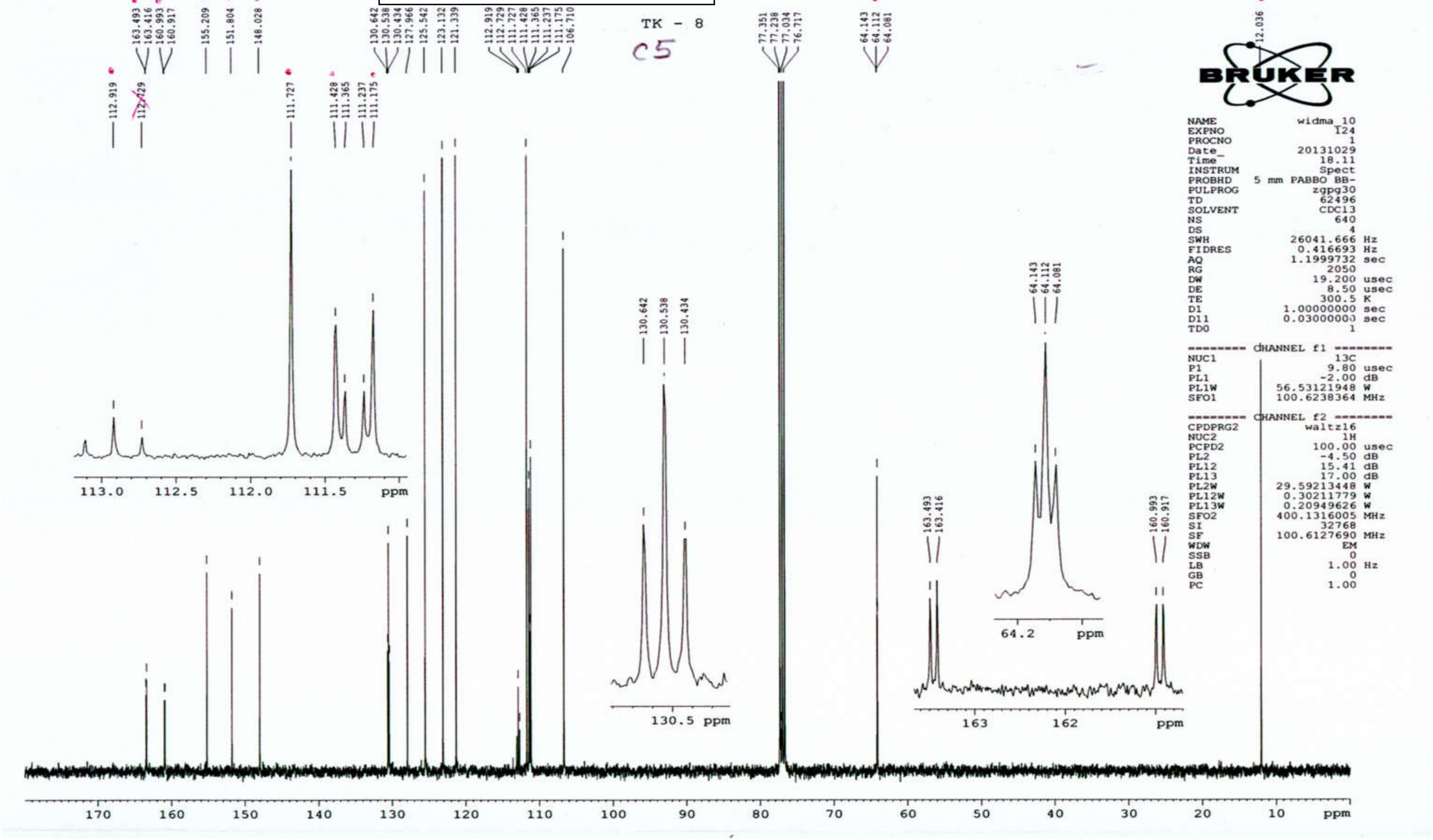

^{13}C NMR spectrum of the compound **7**

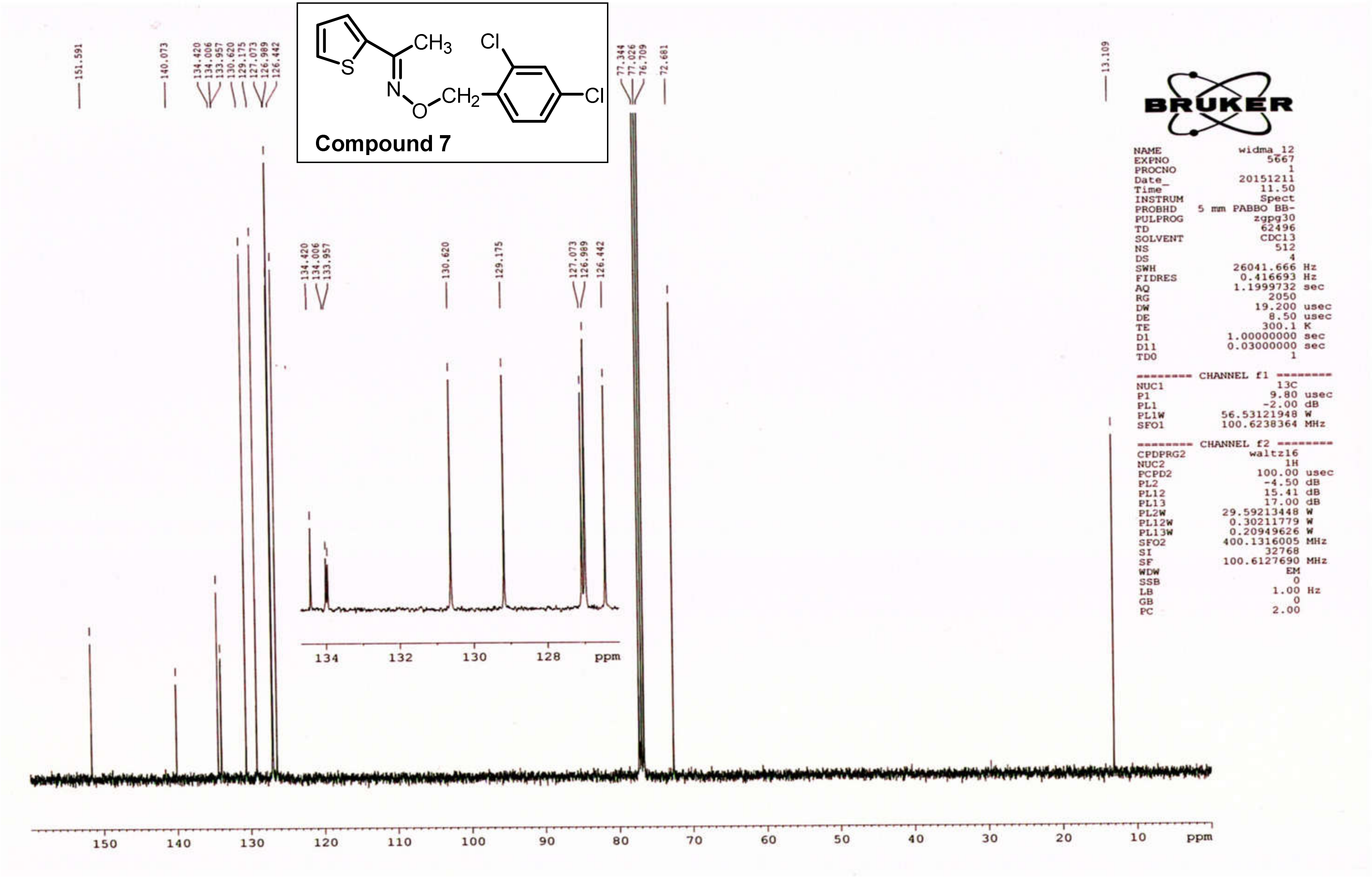

^{13}C NMR spectrum of the compound **8**

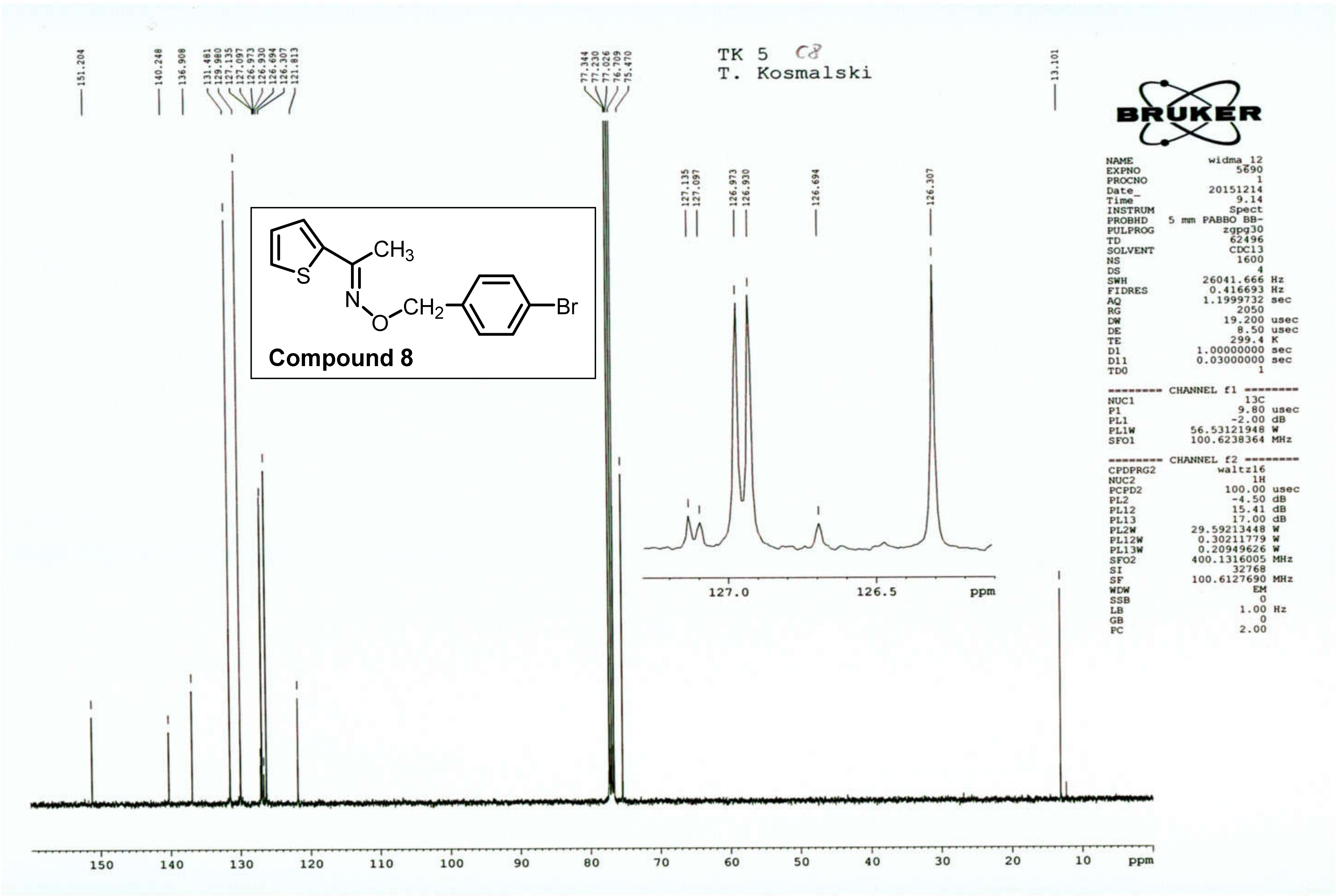

^{13}C NMR spectrum of the compound **9**

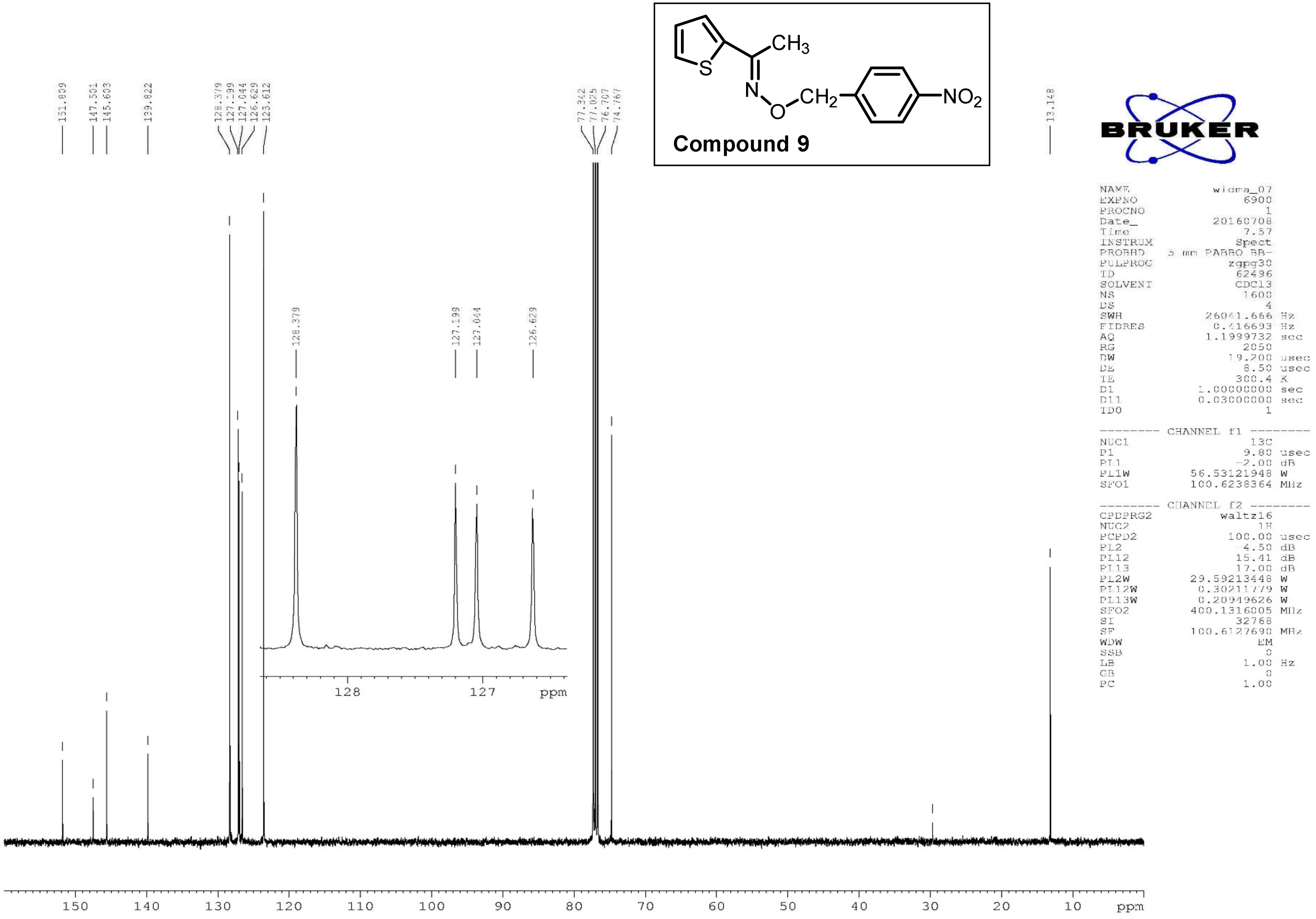

^{13}C NMR spectrum of the compound **10**

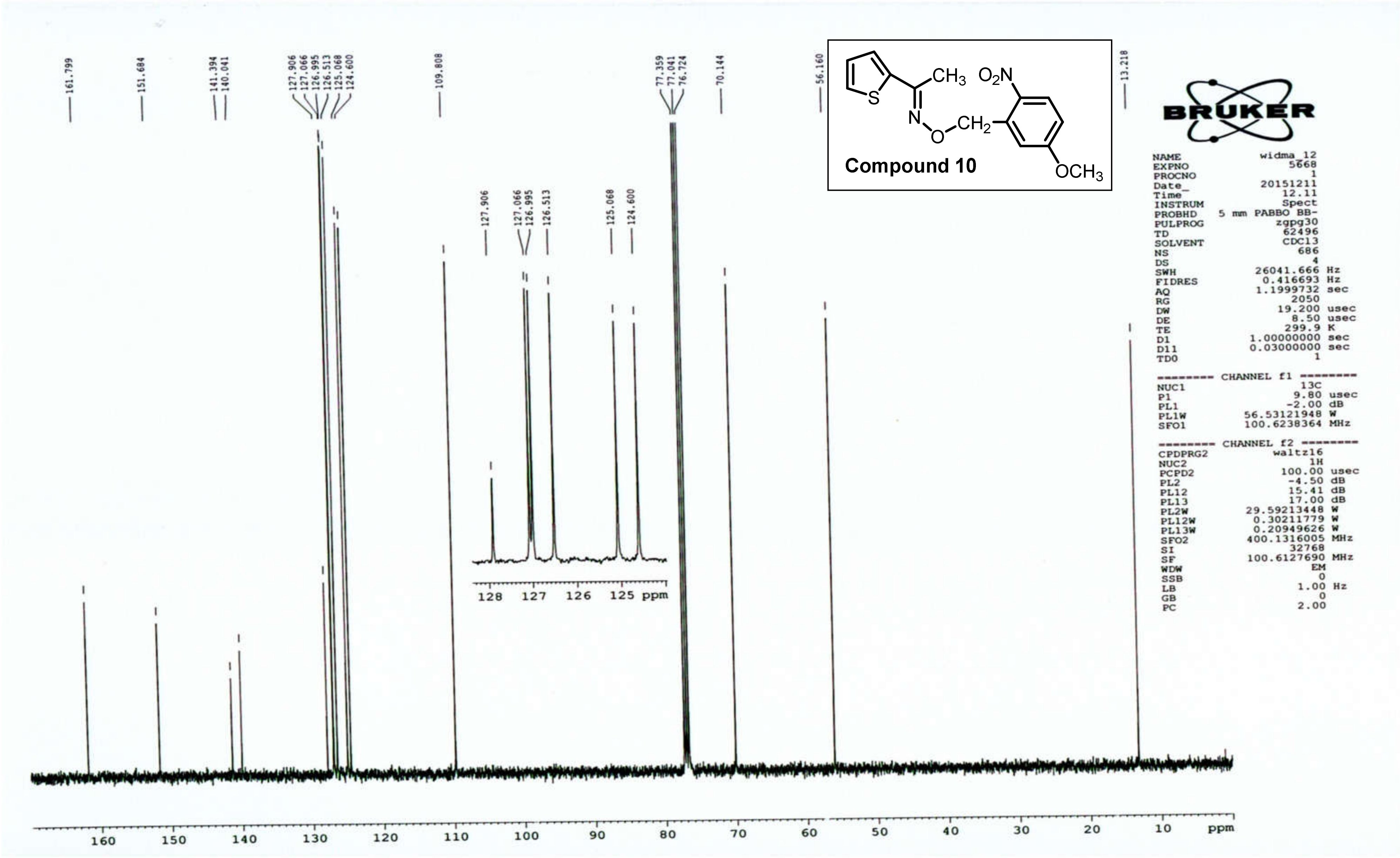

^{13}C NMR spectrum of the compound **11**

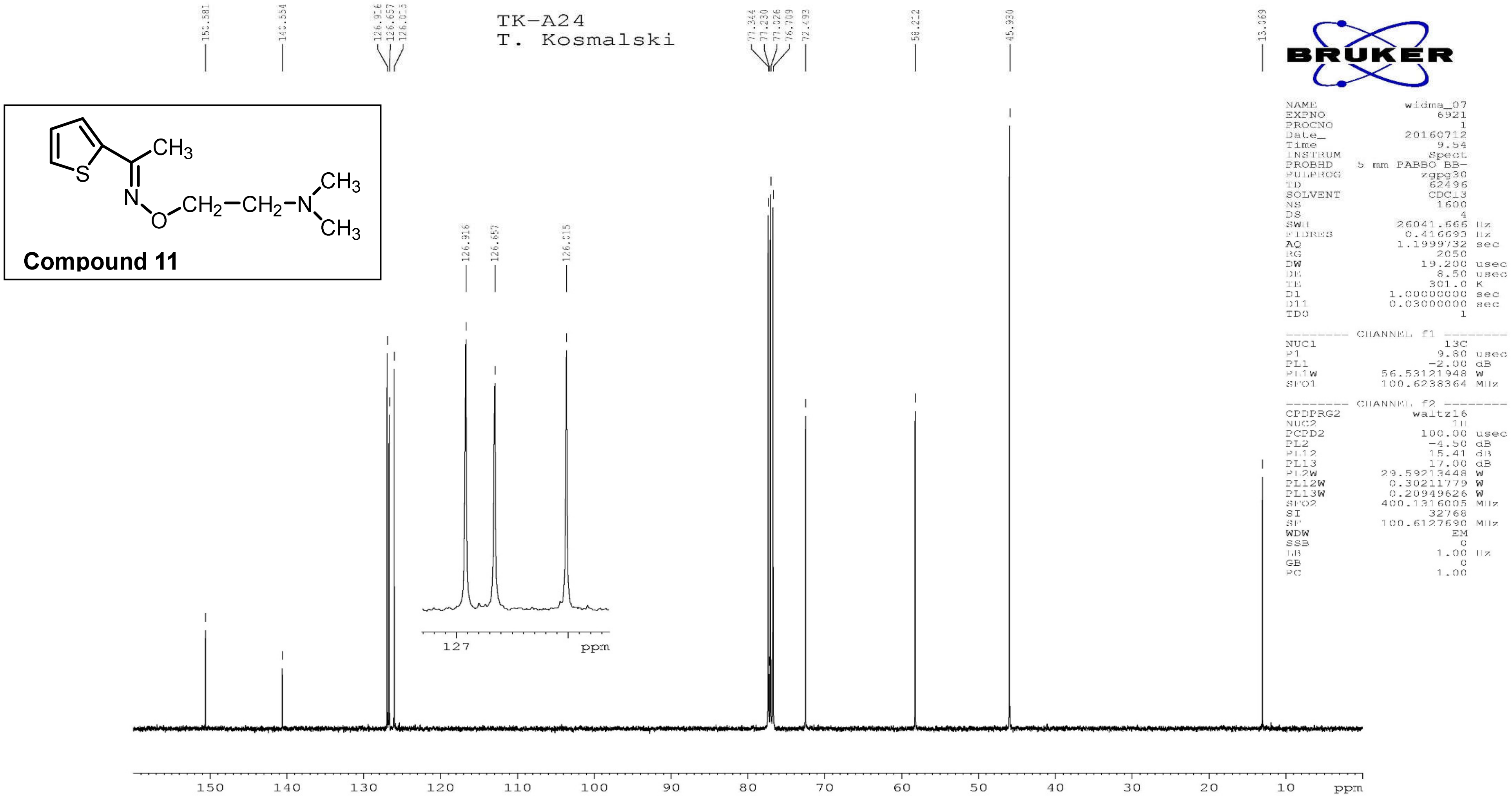

^{13}C NMR spectrum of the compound **12**

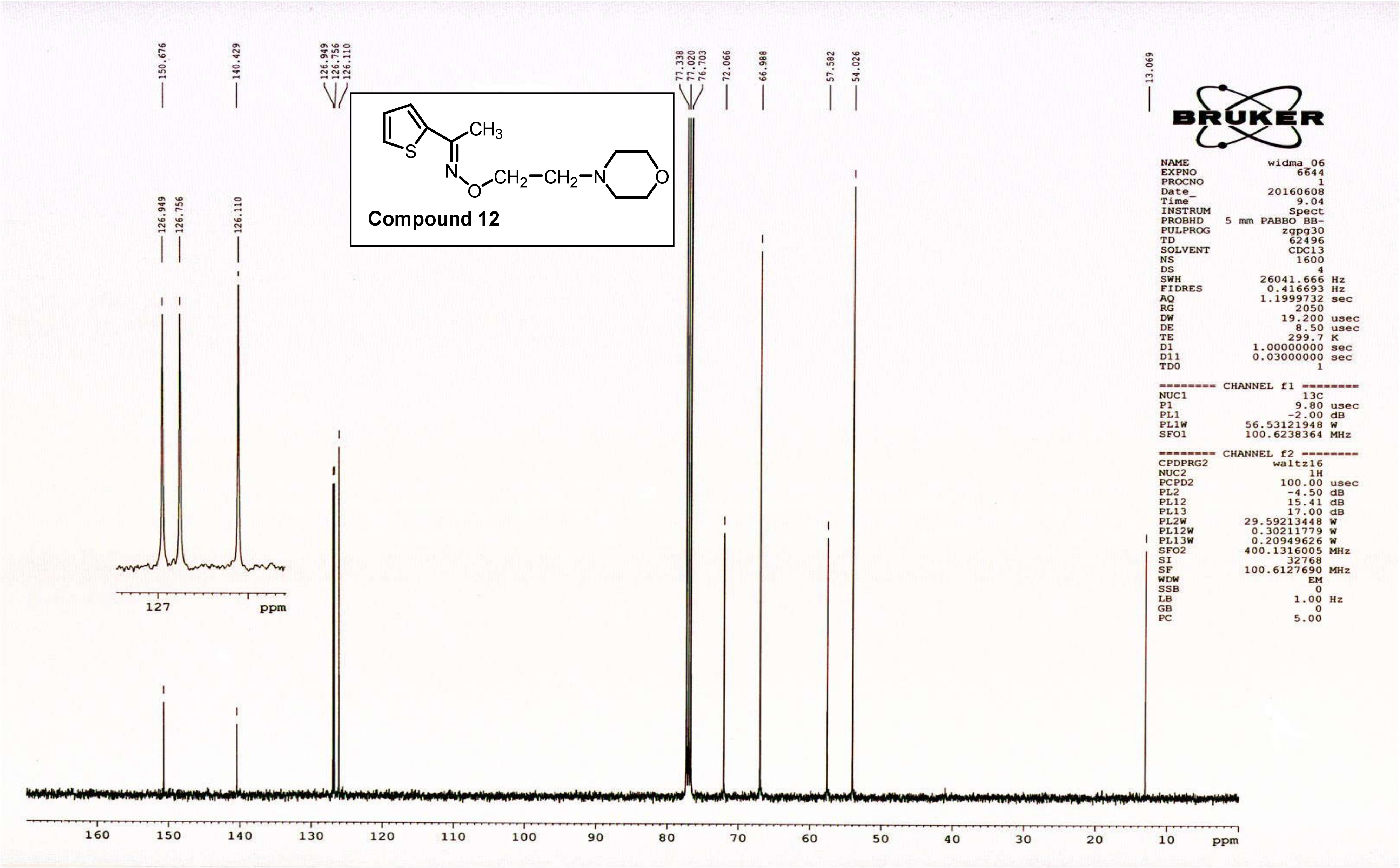

^{13}C NMR spectrum of the compound **16**

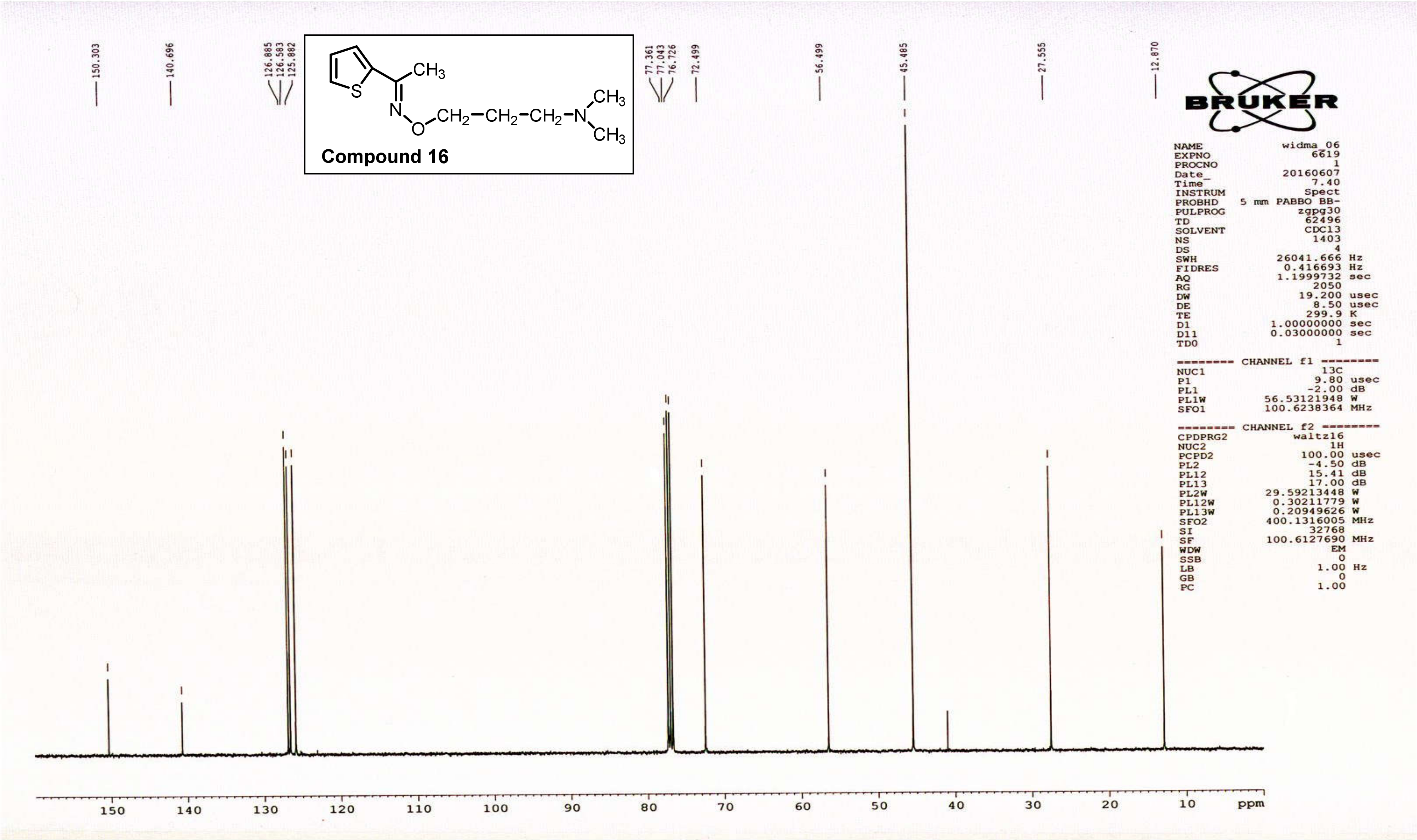

^{13}C NMR spectrum of the compound **17**

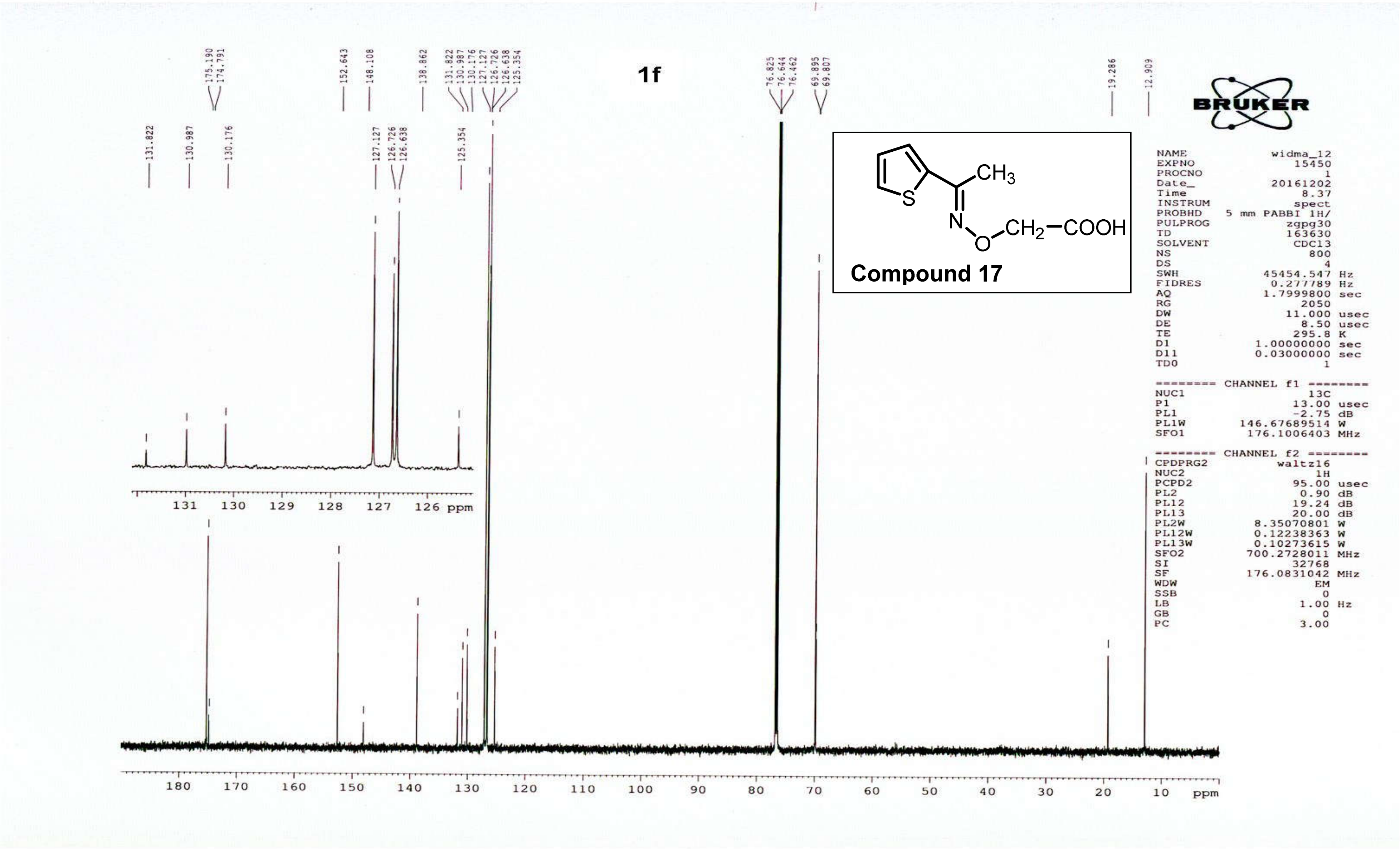

^{13}C NMR spectrum of the compound **18**

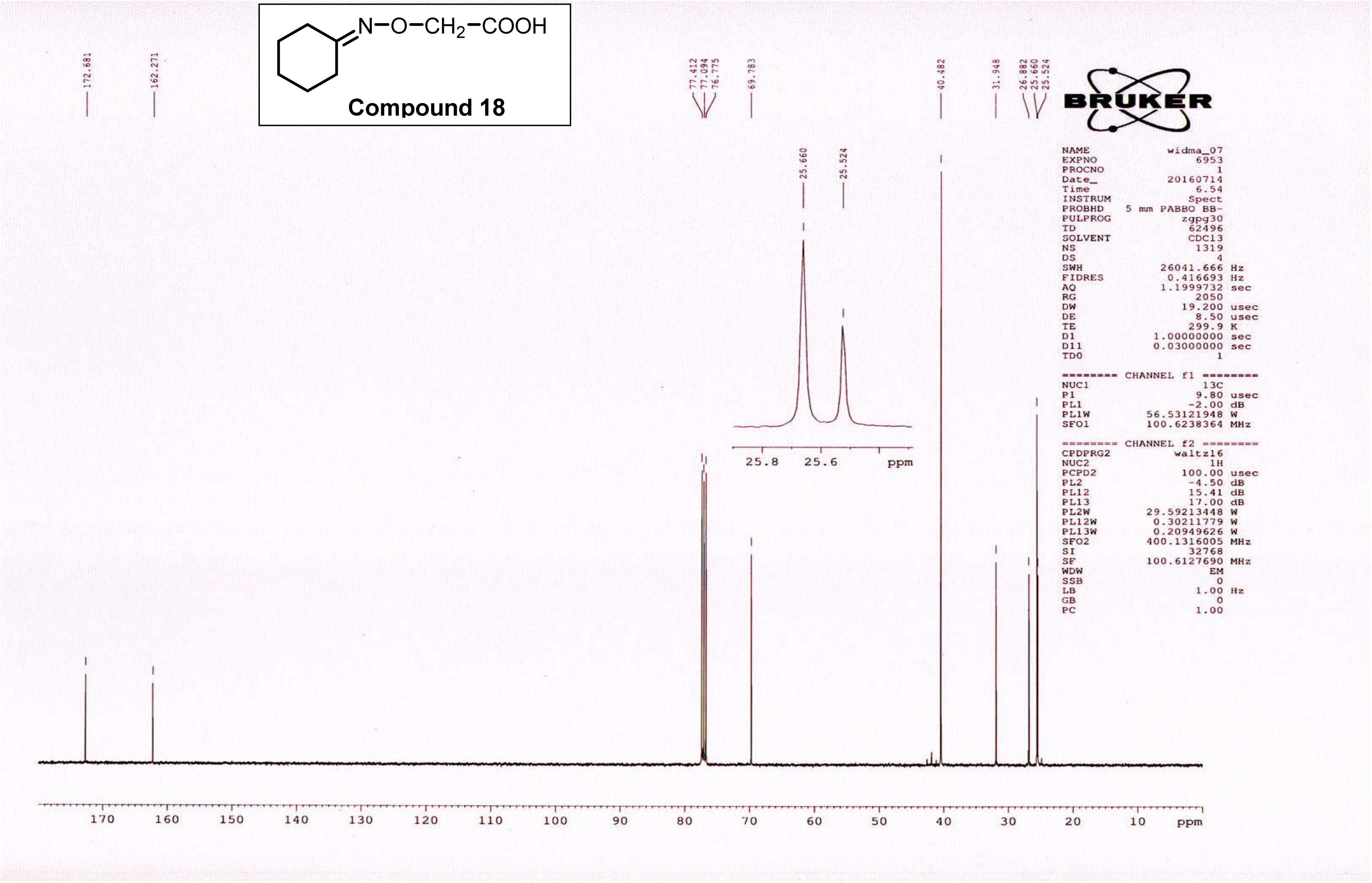

^{13}C NMR spectrum of the compound **19**

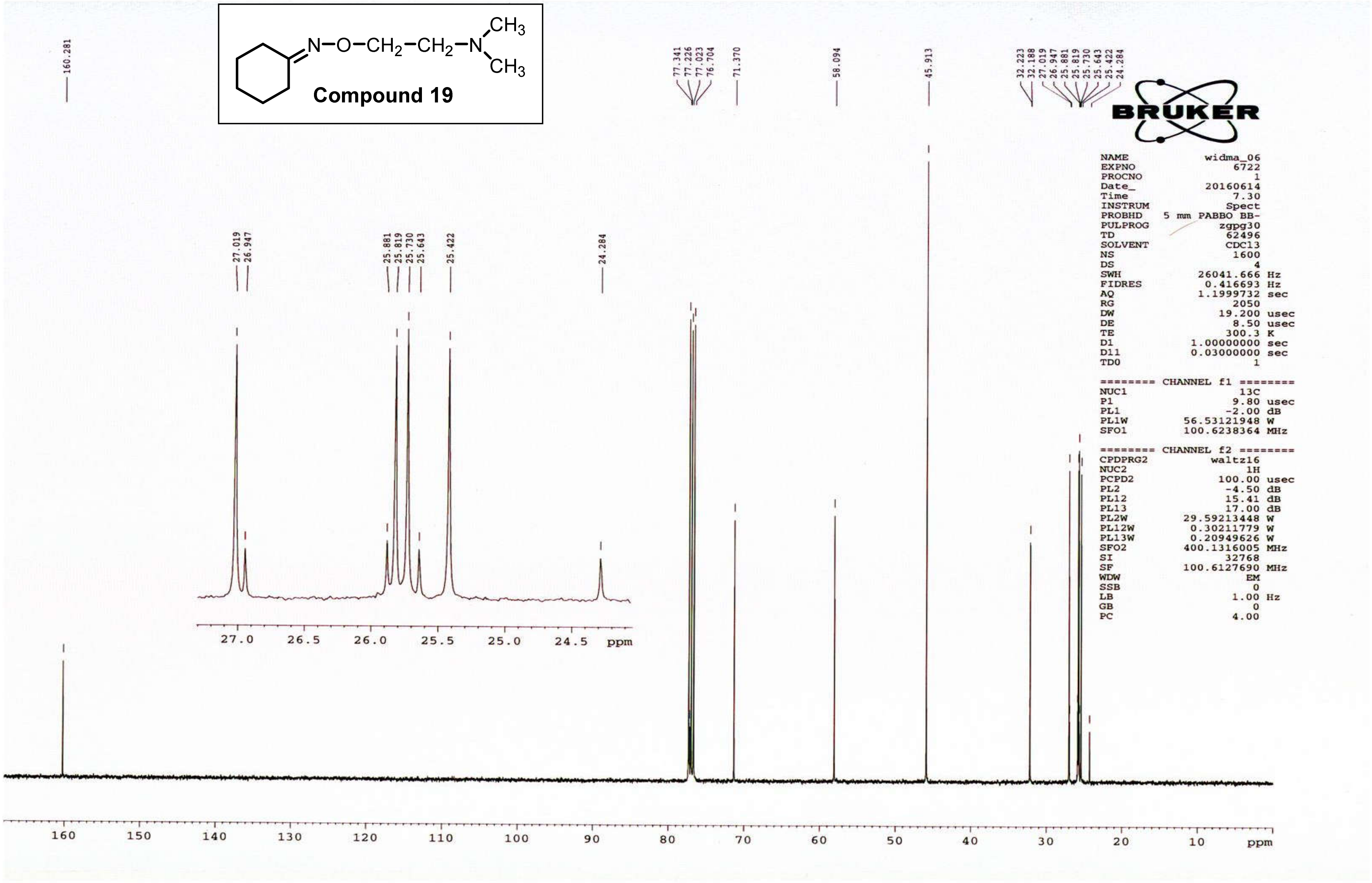

^{13}C NMR spectrum of the compound **20**

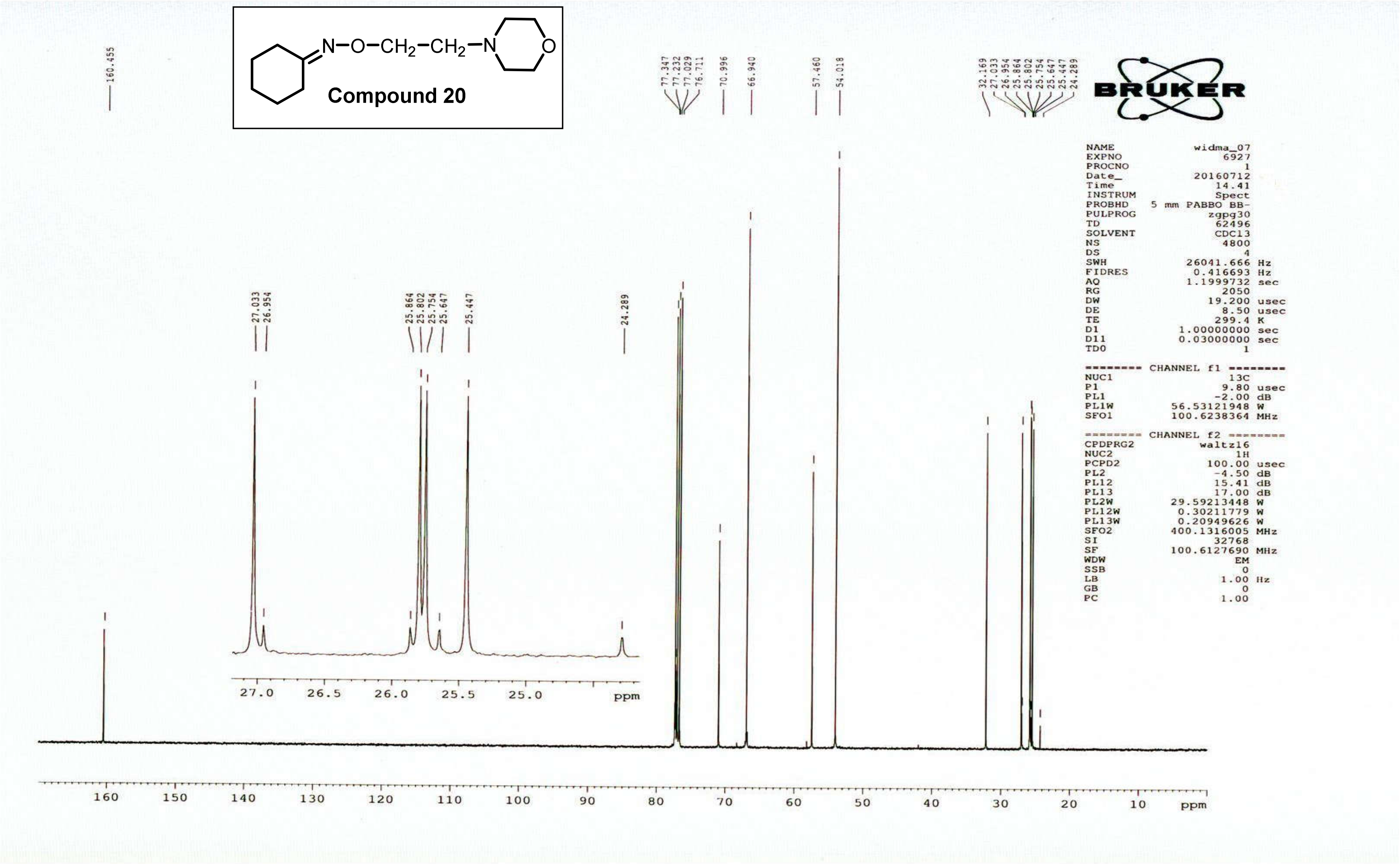

^{13}C NMR spectrum of the compound **21**

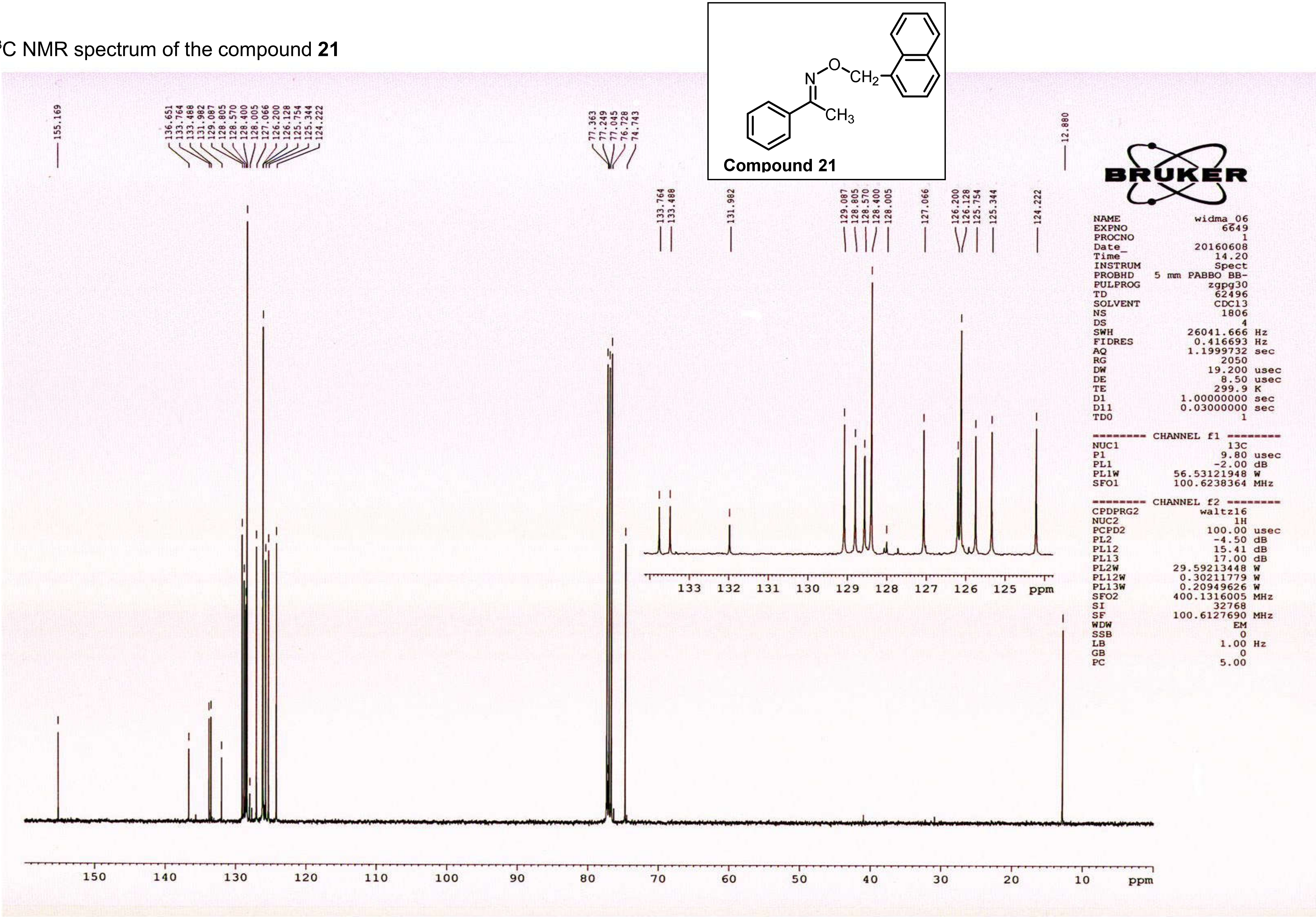

^{13}C NMR spectrum of the compound **22**

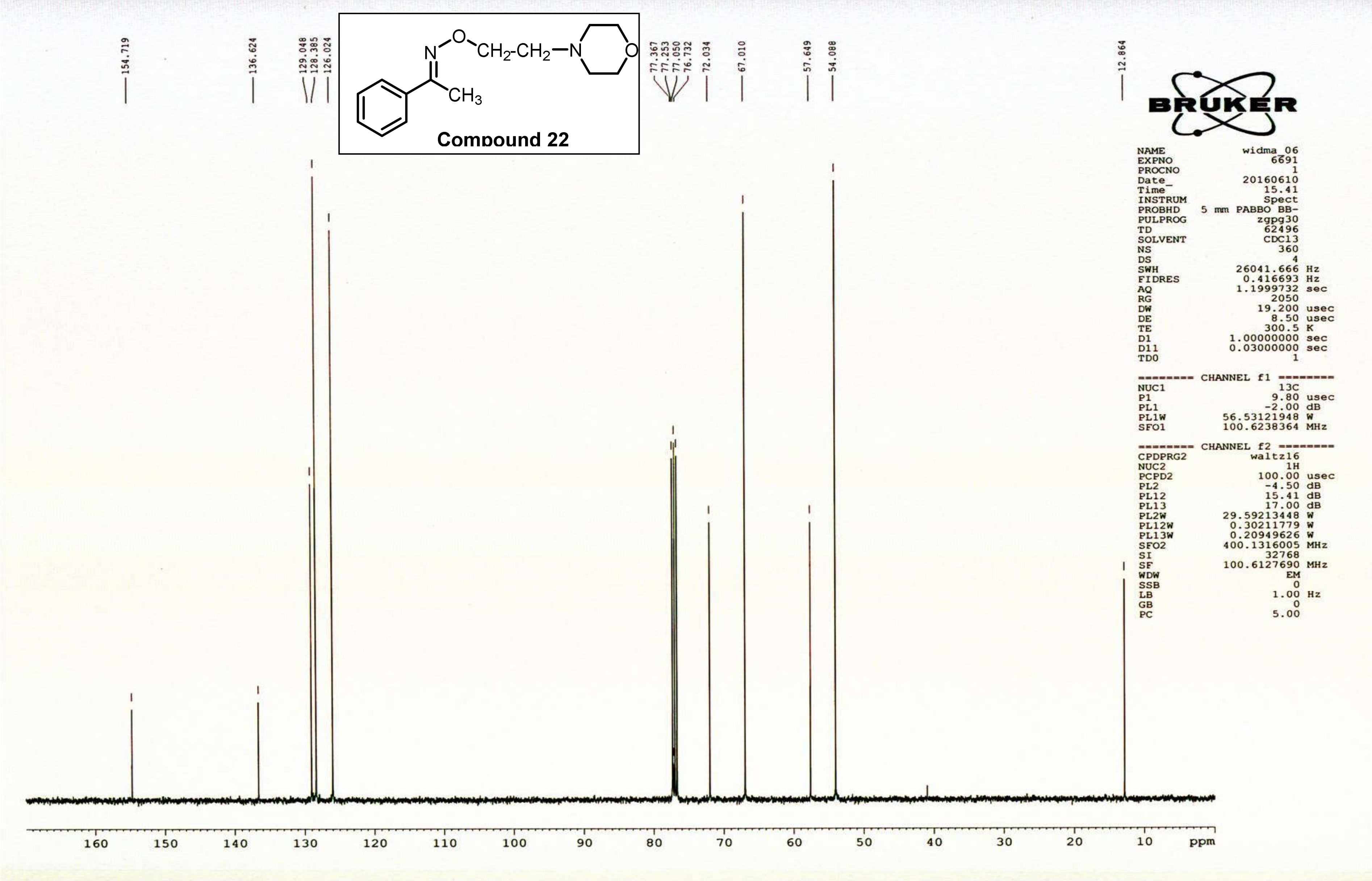

^{13}C NMR spectrum of the compound **23**

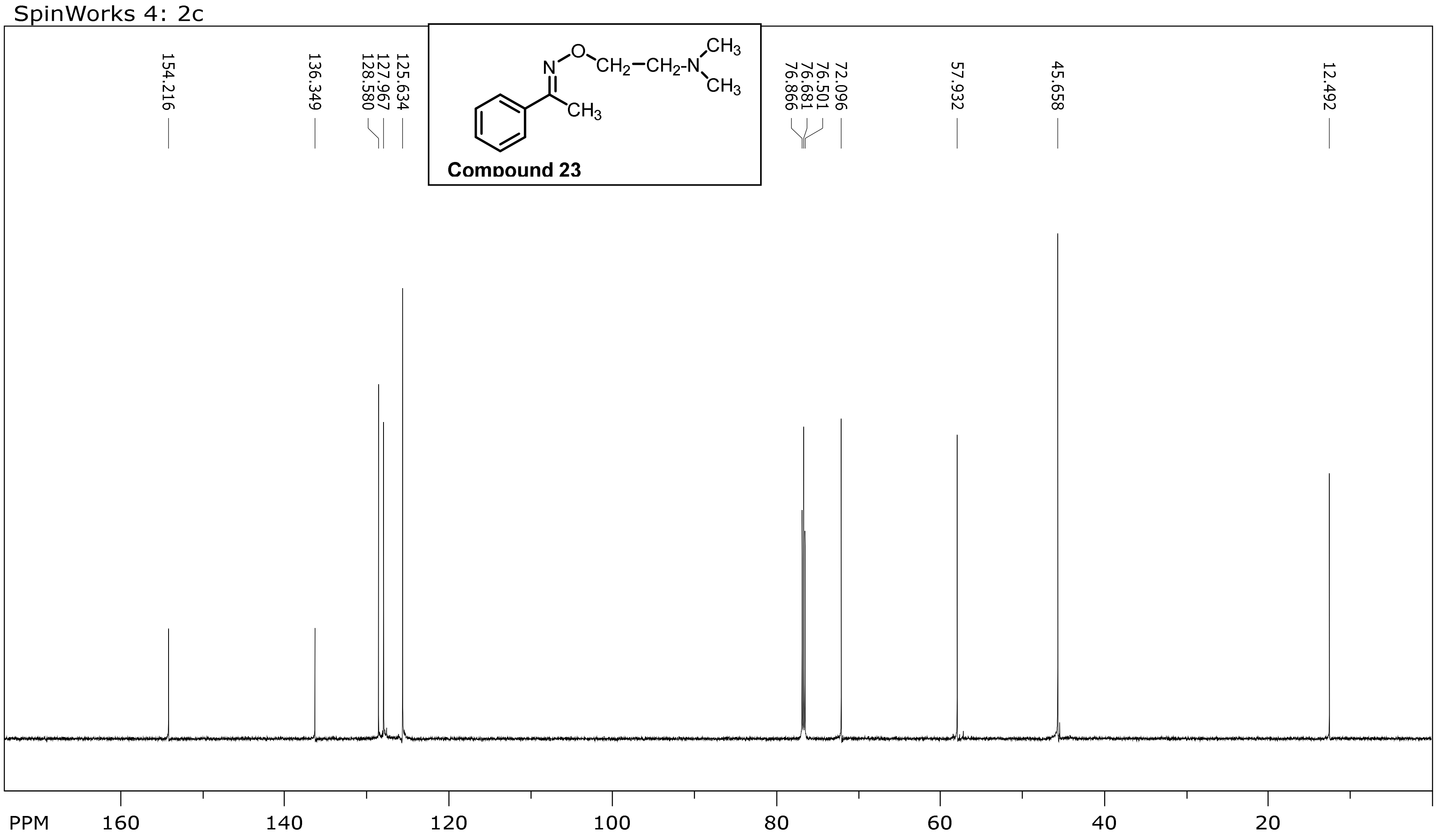

file: ...rs\Tomek\Desktop\NMR 700\15448\fid expt: <zgpg30>
transmitter freq.: 176.100640 MHz
time domain size: 163630 points
width: 45454.55 Hz = 258.1169 ppm = 0.277789 Hz/pt
number of scans: 601

freq. of 0 ppm: 176.083104 MHz
processed size: 16384 complex points
LB: 1.000 GF: 0.0000
